# A systematic review of population based epidemiological studies in Myasthenia Gravis

**DOI:** 10.1186/1471-2377-10-46

**Published:** 2010-06-18

**Authors:** Aisling S Carr, Chris R Cardwell, Peter O McCarron, John McConville

**Affiliations:** 1 Department of Neurology, Royal Victoria Hospital, Belfast, Northern Ireland, UK; 2 Department of Epidemiology and Medical Statistics, Queens University, Belfast, Northern Ireland, UK; 3 Department of Neurology, Ulster Hospital, Dundonald, Northern Ireland, UK

## Abstract

**Background:**

The aim was to collate all myasthenia gravis (MG) epidemiological studies including AChR MG and MuSK MG specific studies. To synthesize data on incidence rate (IR), prevalence rate (PR) and mortality rate (MR) of the condition and investigate the influence of environmental and technical factors on any trends or variation observed.

**Methods:**

Studies were identified using multiple sources and meta-analysis performed to calculate pooled estimates for IR, PR and MR.

**Results:**

55 studies performed between 1950 and 2007 were included, representing 1.7 billion population-years. For All MG estimated pooled IR (eIR): 5.3 per million person-years (C.I.:4.4, 6.1), range: 1.7 to 21.3; estimated pooled PR: 77.7 per million persons (C.I.:64.0, 94.3), range 15 to 179; MR range 0.1 to 0.9 per millions person-years. AChR MG eIR: 7.3 (C.I.:5.5, 7.8), range: 4.3 to 18.0; MuSK MG IR range: 0.1 to 0.32. However marked variation persisted between populations studied with similar methodology and in similar areas.

**Conclusions:**

We report marked variation in observed frequencies of MG. We show evidence of increasing frequency of MG with year of study and improved study quality. This probably reflects improved case ascertainment. But other factors must also influence disease onset resulting in the observed variation in IR across geographically and genetically similar populations.

## Background

Myasthenia gravis (MG) is an archetypal autoimmune disorder in which muscle weakness occurs as a result of impairment of neuromuscular transmission. It is likely to occur as the result of a number of disease entities that result in an indistinguishable clinical picture [1]. There are paraneoplastic forms (thymoma-associated) and non-paraneoplastic forms and the disorder is immunologically heterogeneous - for example serum antibodies can be detected to muscle acetylcholine receptors (AChR-Ab) [2] or the muscle-specific receptor tyrosine kinase (MuSK-Ab) [3], but not both in the same patient. From case series and epidemiological studies, a bimodal distribution of MG IR has been frequently described suggesting a hormonal or environmental influence on disease onset. MG occurs in both sexes, at all ages and in all races [4].

A large number of MG epidemiological studies have been performed worldwide over the last 60 years with marked variability in observed incidence and prevalence of the disease. Systematic review of MG epidemiology has been carried out in the past by Phillips [5]; but 28 further studies have been performed since and this review predates the discovery of MuSK-Ab MG. In his review, Phillips commented upon apparent increasing IR and PR with time but without similar change in MR, and proposed that these trends were due to improved diagnosis and a changing natural history of disease related to better treatment.

The aim of this review was to summarize the findings of all population-based epidemiological studies of myasthenia gravis (MG) paying attention to serological subtype-specific studies and age- and sex- specific incidence. We sought to establish if there is a consensus in IR, PR and MR of MG worldwide and investigate any trends or differences in observed rates over time and in populations.

## Methods

To identify all relevant studies, 4 medical databases (Pubmed, Medline, EMBASE, Cochrane library) and 2 proceedings databases (Zetoc, ISI proceedings) were searched using 21 search terms as keywords or MESH terms (Additional file 1 shows search strategy and terms used). English and non-English studies were included and non-English studies were translated when required. The bibliographies of all included studies were searched and the final list was discussed with experts in the field (Professor Angela Vincent, Neurosciences group, Oxford; Dr J McConville, Belfast). Good overlap between sources assured almost complete ascertainment of relevant studies (See additional file 1).

All population-based epidemiological studies of myasthenia gravis were included and case series without a defined denominator population were excluded. Inclusion criteria for MG cases in individual studies were broadly based on Osserman clinical criteria [6] with supportive evidence from anti-acetylchoinesterase responsiveness, neurophysiological and serological testing. There are no universally accepted and validated criteria for the diagnosis of MG therefore we accepted all cases as defined by the authors. The majority of studies considered all patients with a clinical diagnosis of MG together (henceforth referred to as All MG). A proportion of studies performed in the past 20 years examined serological MG subgroups separately: anti-acetylcholine receptor antibody positive MG (AChR-MG) and anti-muscle specific kinase antibody positive MG (MuSK-MG). There were no epidemiological studies on seronegative MG (SNMG).

Crude data (number of incident cases, number of prevalent cases, prevalent population, number of population years studied, and number of sources used for case ascertainment, inclusion and exclusion criteria, final-year of study and country of study) were extracted using a piloted questionnaire. IR, PR and MR and exact 95% confidence intervals (CI) based upon the Poisson distribution were calculated using STATA (Copyright 1996-2009 Stata Corp LP). Where the relevant crude data were available (9 studies), a standardised IR was calculated to WHO standard world population [7]. There was no significant difference between crude and standardised rates so crude rates are used throughout this review. Each included study was graded as 'High', 'Intermediate' and 'Low' quality according to number of ascertainment sources used and number of person years studied (details in additional file 1). Search strategy, study selection, data extraction and quality grading were performed by AC and independently conducted by JMcC with good agreement.

Meta-analysis was performed using the random effects model with 95% confidence intervals to the Poisson distribution. Heterogeneity was tested using Chi-squared test and measured using the I^2 ^statistic [8]. A value of I^2 ^< 25% was chosen to represent an appropriate level of homogeneity for calculation of a pooled estimate. Meta-regression analysis was used to investigate the association between rates and study characteristics such as year, latitude and quality of study. Publication bias was assessed using funnel plots.

## Results

From 4030 electronic hits, 114 papers were read in full and 55 selected for inclusion in the review (Additional file 2: Included studies; Additional file 3: Excluded studies).

The 55 studies [4,9-62] included 8033 cases from 1.7 × 10^9 ^person years studied; 47 studies examined ALL MG, 9 studies examined AChR MG and 2 studies examined MuSK MG. One study provided rates on All MG, AChR MG and MuSK MG [54] and two provided rates for All MG and AChR MG within the same populations [50,55]. Additional file 2 lists all included studies, descriptive statistics and quality grading.

**Figure 1:**
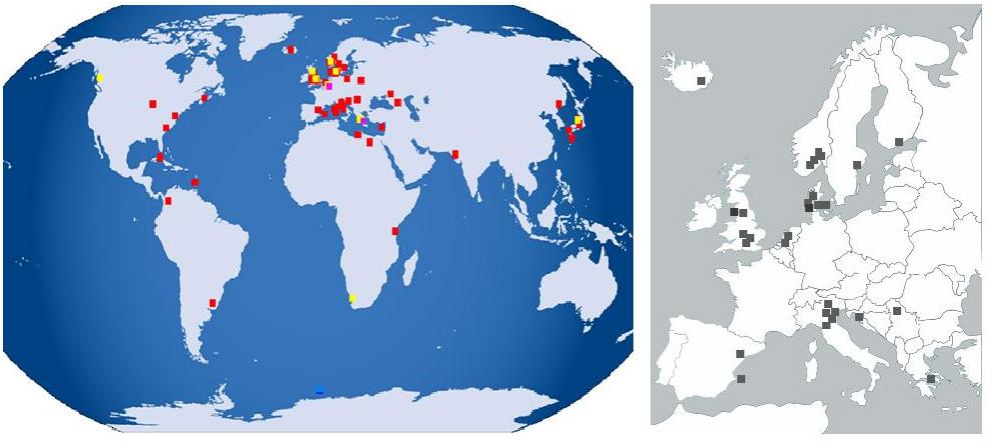
**Geographical distribution of epidemiological studies in myasthenia gravis**. Studies of all patients with autoimmune myasthenia gravis: RED. AChR MG specific studies: YELLOW. MuSK MG specific studies: PINK. Broad geographical distribution of included studies is shown with a preponderance of European studies.

The time period studied ranged from 1950 to 2007. There was wide geographical distribution of studies, with representation of all continents except Australia (Figure 1). 34/52 studies have been performed in Europe with particular contribution from Scandinavian countries (9 studies), northern Italy (5 studies) and the UK (6 studies).

### Incidence rates

**Figure 2:**
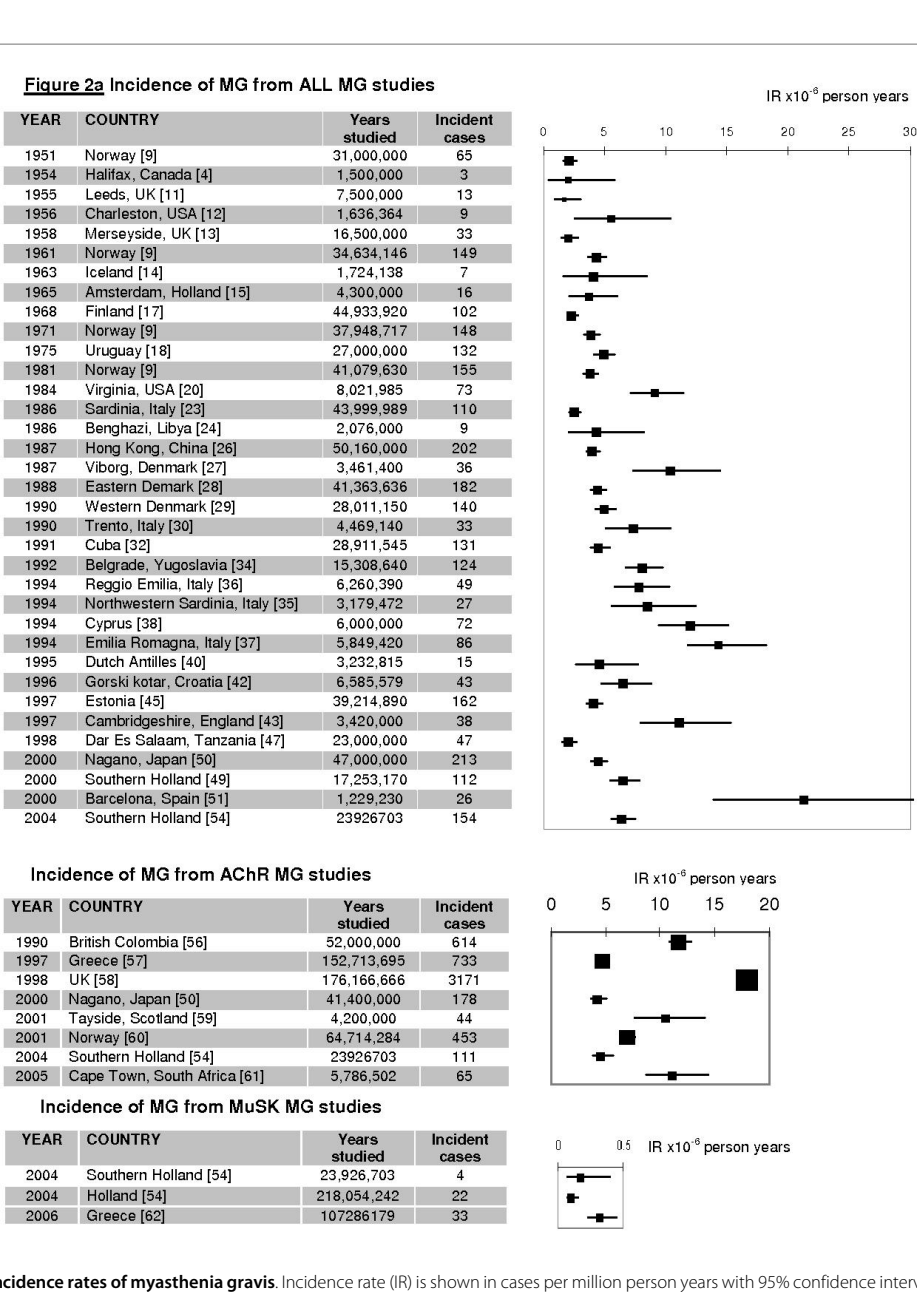
**Incidence rates of myasthenia gravis**. Incidence rate (IR) is shown in cases per million person years with 95% confidence intervals according to the Poisson distribution and weighting according to the random effects model. The variation between rates, or heterogeneity, leaves the estimated pooled IR difficult to interpret so it is better to summarise this data using ranges. All MG: estimated pooled IR (eIR) = 5.3 cases per million person years (C.I.: 4.41 - 6.1), range = 1.7 to 21.3; AChR MG: eIR = 7.3 (C.I.: 5.5, 7.8), range = 4.3 to 18.0; MuSK MG: range = 0.1-0.32.

For the All MG group 35 studies examined incidence. IR ranged from 1.7 to 21.3 cases per million person-years (Figure 2a). The forest plot illustrates a marked variation in IR between studies, even when outliers are excluded [51]. Pooled estimate IR (eIR) was calculated as 5.3 per million person-years (C.I.: 4.41 - 6.12). However the marked heterogeneity across studies, I^2 ^= 96% (C.I.: 95-98%) undermined the validity of the pooled estimate as a generalisable statistic.

Likewise, heterogeneity is seen in the AChR MG group (8 studies examined incidence, Figure 2b) with an I^2 ^= 100%. AChR MG IR ranged from 4.3 to 18.0 per million person-years with a pooled AChR MG eIR of 7.3 per million person-years (C.I.: 5.5, 7.8).

Only 2 epidemiological studies have been performed to date on MuSK MG, in Holland [54] and Greece [62]. In Holland IR: 0.1 per million person-years (C.I.: 0.07, 0.15), and in Greece IR: 0.32 per million person-years (C.I.: 0.00, 1.32).

The observed heterogeneity might be explained by a number of factors; either biological or technical. We investigated how the effects of year of study, geographical area of study and study quality upon observed frequency.

**Figure 3:**
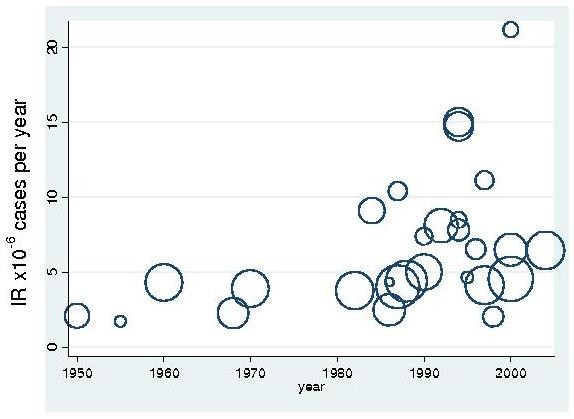
**Incidence rate and prevalence rates with time**. IR in cases per million person years and PR in cases per million persons are plotted against final year of study. The area each circle is proportional to the size of the study population.

Linear regression of IR against the final year of study suggests a significant correlation (Meta-regression p:0.0001, r^2^:0.12) equivalent to a 3% increase per year (Figure 3). In 1976 a version of the modern acetylcholine receptor antibody assay was first described [2]. There was no evidence of a reduction in heterogeneity when studies were grouped into those published before (I^2 ^= 91%, C.I.:86, 94%) and after this date (I^2 ^= 95%, C.I.:93, 96%). However, the average incidence rate after 1976 (eIR = 6.5 per million person-years, C.I.:5.3, 7.9) was significantly higher (t test: p = 0.0001) than before 1976 (eIR = 3.5 per million person-years, C.I.: 2.7, 4.4) corresponding to an approximate doubling (Rate ratio: 1.86, C.I.:1.22, 2.88).

There was no significant reduction in the heterogeneity of IR when studies were grouped by geographical area: Northern Europe I^2 ^= 94% (C.I.:91, 95%), Southern Europe I^2 ^= 97% (C.I.:96, 98%), North America and Canada I^2 ^= 67% (C.I.:2, 89%). The limited numbers of studies in Asia, Africa or Central/South American sub-groups prevented similar analysis. There was no evidence of a difference (p = 0.47) between the area-specific pooled estimate IR: Northern Europe (eIR = 4.6 per million person-years, C.I.: 3.8, 5.7), Southern Europe (eIR = 4.9 per million person-years, C.I.: 4.9, 16.3), North America and Canada (eIR = 5.3 per million person-years, C.I.: 2.8, 10.0), Central and South America (eIR = 4.32 per million person-years, C.I.:3.8, 4.8) and Asia (eIR = 4.7 per million person-years, C.I.: 4.2, 5.3).

There was a trend to decreasing heterogeneity with increasing study quality: High (I^2 ^= 76%, C.I.:49,89%), Moderate (I^2 ^= 89%, C.I.:85,92%) and Low (I^2 ^= 96%, C.I.:93, 97%). There was also evidence of a significant difference (p = 0.009) in the pooled estimate IR in the High quality studies (eIR = 9.4 per million person-years, C.I.:7.7, 11.4) and the pooled estimate IR in Intermediate and Low quality studies (eIR = 4.5 per million person-years, C.I.:3.8, 5.2).

### Age and sex specific incidence rates

**Figure 4:**
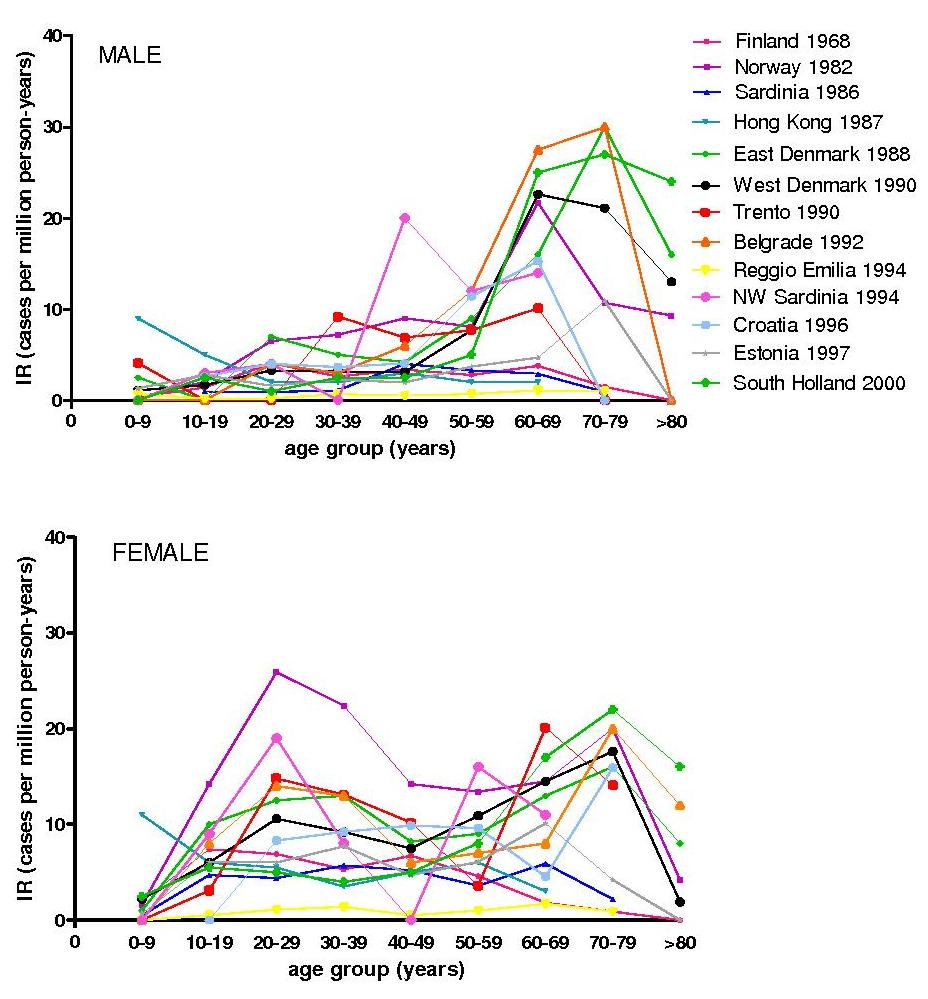
**Age and sex specific incidence rates**. Age and sex specific IR is shown in cases per million person years. An increasing frequency of disease with age is seen in all populations. However, only 5 out of 14 shown a bimodal distribution in IR with the peak in younger women not observed in the remaining 9 studies.

14 studies provided data required to allow analysis of incidence rate by age and sex (Figure 4). A bimodal distribution in IR in females was observed in 5 of 14 studies. IR in both sexes increased with age, peaking between 60 to 80 years in all but 2 studies, with apparent male predominance in the older age group. The Asian study stands out in that the proportion of childhood onset MG (onset < 15 years) appears to be higher in this population [26].

### Prevalence rates

**Figure 5:**
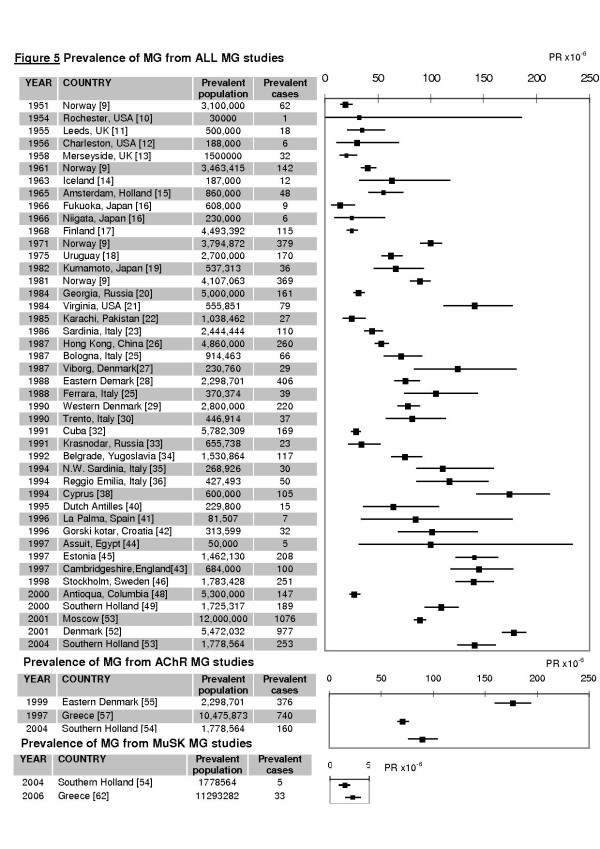
**Prevalence rates of myasthenia gravis**. Prevalence rate (PR) is shown in cases per million persons with 95% confidence intervals (95% C.I.) according to the Poisson distribution and weighting according to the random effects model. All MG ePR = 77.67 cases per million (C.I.: 63.98, 94.30), range = 15 to 179 cases per million; AChR MG: range = 70.6 to 163.5; MuSK range = 1.9-2.9.

For the All MG group 44 studies examined prevalence. The observed PR ranged from 15 to 179 per million (Figure 5). The estimated pooled PR is 77.7 cases per million (C.I.: 63.98, 94.30) but, as for IR there was marked heterogeneity with I^2 ^= 98% (C.I.: 97-98%) observed across studies limiting interpretation.

For the AChR MG group, 3 studies provided prevalence data. The prevalence of AChR MG ranged from 70.6 to 163.5 per million. The marked variation between rates is graphically depicted by the forest plot. The observed prevalence for MuSK MG in Southern Holland is1.9 per million (C.I.: 1.2, 2.6), representing 2% of prevalent MG cases in the region. MuSK-MG PR in Greece is higher at 2.9 per million (95% C.I.: 1.92, 3.92).

There was a linear trend to increasing PR with year of study (Meta-regression p = 0.0001, r^2^: 0.41). There is a doubling in PR after 1976; RR = 2.2 (C.I.: 1.36, 3.14). PR is on average 15-fold higher than IR for all studies (mean PR:IR ratio: 14.7, S.D.:6.0, range: 3.5 to 34.6), there is a trend to increasing PR:IR ratio with time (Linear regression p:0.0496, r^2^:0.12).

### Mortality rates

Mortality rates as deaths due to MG per million person years were examined (Additional file 2). Seven All MG studies and 1 AChR MG study provided this information. The MR ranges from 0.06 to 0.89 per million person-years. The MR for AChR MG in Greece lies within this range: 0.43 per million person-years (C.I.:0.34, 0.55).

No trend was observed for MR with year of study.

### Publication bias

Symmetrical funnel plots suggest absence of marked publication bias (Additional data file 1).

## Discussion

Meta-analysis of observational studies is complicated by intrinsic differences between the populations being studied and variation in methodological quality of studies included; nevertheless the heterogeneity across studies in this review was marked. The degree of dissimilarity in IR, PR and MR of MG across populations renders the calculated pooled estimates of limited generalizability and validity. Nonetheless crude estimates are possible using the combination of pooled estimates and the range of observed frequencies. All MG eIR: 5.3 per million person-years (C.I.: 4.41, 6.12), range: 1.7 - 21.3; ePR: 77.67 cases per million (C.I.: 63.98, 94.30), range: 15 - 179; MR range: 0.06 - 0.89 per million person-years. AChR MG eIR: 7.3 per million person-years (C.I.: 5.5, 7.8), range: 4.3 - 18.0; PR range: 70.6 - 163.5 per million. MuSK MG IR range: 0.1 - 0.32 per million person-years; PR range: 1.9 - 2.9 cases per million.

In keeping with Phillips' observations [5] an increasing trend in IR and PR with time was observed. However this appears to be bimodal rather than linear in pattern, with estimated rates for incidence and prevalence approximately doubling around the mid-1980 s. This may be best explained by the influence of anti-AChR antibody receptor assay upon ascertainment but improving epidemiological methodology must also play a role. Our data shows a doubling of the pooled eIR in the 'High' quality studies when compared with that from the 'Low' and 'Intermediate' quality studies. As all 'High' quality studies were performed since 1987 it may be more accurate to use this data to estimate All MG incidence rate: eIR = 9.4 cases per million person years (C.I.:7.7, 11.4), range 7.4-14.7 cases per million person years. Despite a reduction in IR heterogeneity in this group it remained significant suggesting that these technical issues only contribute to the variation seen and do not explain it completely.

Several observations suggest that biological factors are important. Age and sex specific incidences show prominent differences between populations, with a peak in the incidence of MG in young women being observed in five of fourteen studies only. Childhood MG (onset < 15 years) was higher in the one Asian study [26] examined by age and sex. MuSK MG was described in only two population-based studies. Evidence from case series suggests differing frequency of MuSK MG across populations, with decreasing frequency with distance from the equator [63]. This suggests that at least some of the observed heterogeneity might be due to differing frequencies of MG subgroups between populations. Differences in population genetics may go some way to explain the variability in rates. For example, different HLA associations have been described in MG subsets: early onset MG, late onset MG, MuSK-MG. These HLA associations are different in North American/European populations versus Asian populations [1].

The greatest heterogeneity was observed in the AChR MG group. It might have been expected that these studies represent a more homogenous patient group than the broader All MG category. Five of nine studies in the AChR group were carried out with identical methodology. These 5 studies [55-58,60] were performed using records of anti-AChR testing from national or regional immunology laboratories, the assumption being that the first positive titre for an individual correlates with disease onset. If this were true for all AChR MG patients then any differences observed between the IR in the studies should be due to differences in population genetics and environmental factors influencing frequency of disease. For example the Greek study [57] and the UK study [58] were performed with identical methodology over similar time periods so the 3 fold difference in frequency is notable: Greek IR 4.8 per million person years (C.I.: 4.5, 5.2), UK IR 18.0 per million person years (C.I.: 17.4, 18.6). However this does not take into account the differing levels of access to a neurologist and access to serological tests between populations.

PRs were on average 15 fold higher than IR across studies. This difference could be explained by good survival and low mortality associated with MG, however, an older age of symptom onset probably limits overall survival therefore influencing PR. We also saw a trend to increasing prevalence compared to incidence (PR: IR ratio) with year of study which may suggest improving care and survival and increasing life expectancy.

It is notable that no data were available on seronegative MG. Its epidemiology, therefore, remains undescribed.

## Conclusions

IR and PR of MG vary markedly between populations studied. Pooled incidence rates cannot readily be extrapolated to unstudied populations. The heterogeneity may be explained in part by methodological differences but the data also suggest that there are biological factors to account for differences. Detailed population-based data on serological and pathological sub-types of MG within complete populations are largely lacking.

Future studies should concentrate on accurate clinical case definition over adequate time periods in sufficiently sized populations. Factors influencing ascertainment such as equity of access to appropriate specialists in neurology and ophthalmology should be noted. National laboratory records of positive AChR or MuSK antibody titres used in isolation probably under-estimate rates so multiple sources of ascertainment should be used in order to optimise case identification. Serological sub-group classification is important and recently developed assays for the identification of MuSK MG and MG with antibodies to AChR clustered with rapsyn [64] will aid diagnosis and sub-group phenotype description.

## Competing interests

The authors declare that they have no competing interests.

## Authors' contributions

AC designed and performed the literature review, collected data from individual studies, participated in statistical analysis, data interpretation and was primary author of the manuscript. CC participated in study design and performed statistical analysis. PMcC participated in study design and drafting of the final manuscript. JMcC conceived the study, and participated in its design and coordination and helped to draft the manuscript. All authors read and approved the final manuscript.

## Pre-publication history

The pre-publication history for this paper can be accessed here:

http://www.biomedcentral.com/1471-2377/10/46/prepub

## Supplementary Material

Additional file 1 **MOOSE guidelines**. Description of performance of the study in accordance to MOOSE (Meta-analysis of observational studies) guidelines including details of literature search. Click here for file

Additional file 2 **All included studies**. All included studies are listed with reference (ref), IR: Incidence rate (cases per million person years), PR: Prevalence rate (cases per millions), MR: Mortality rate (deaths due to MG per million person years), 95% C.I., S.E.: standard error and quality grading (1: High, 2: Intermediate, 3: Low). Click here for file

Additional file 3 **All excluded studies**. Table (iii)a List of studies excluded on examination of full text (or abstract only from proceedings papers) with reason for exclusion. Proceedings papers (P) Table (iii)b List of studies excluded on the basis of title and/or abstract from initial database searches (Medline, EMBASE and first 250 hits from Pubmed only). Click here for file
