# Supplementary material for: A systematic review of population based epidemiological studies in Myasthenia Gravis
Source: BMC Neurol. 2010 Jun 18;10:46. doi: 10.1186/1471-2377-10-46 (PMC2905354; doi:10.1186/1471-2377-10-46)
Supplement: Additional file 1 — MOOSE guidelines. Description of performance of the study in accordance to MOOSE (Meta-analysis of observational studies) guidelines including details of literature search. [file 1471-2377-10-46-S1.DOC]

# **MOOSE Checklist**

Background

##### Problem definition

- What is the epidemiology of myasthenia gravis (MG)?
- From the existing literature, is there a worldwide consensus for incidence rate (IR), prevalence rate (PR) and mortality rate (MR) of this condition?
- Do IR, PR or MR of myasthenia gravis change over time, by geography or do other factors affect the observed rates?

##### Hypothesis statement

- We propose that differences observed in incidence, prevalence and mortality rate of MG across studies are likely to be due to differences in case ascertainment and study methodology but may be influenced by biological factors aswell.

##### Study outcomes

Individual study statistics examined included

- Incidence of MG: Number of new cases of myasthenia gravis per million person years studied
- Prevalence of MG: Number of cases of myasthenia gravis per million persons in a population at a defined date (prevalence day)
- Mortality rate: Number of deaths due to MG per millions person years studied
- Age and sex incidence was also studied where data was available

##### Type of exposure or intervention used

- No exposure or intervention was tested in the studies included in this review
- Cases of MG were studied and observed characteristics recorded

##### Type of study design used

- Population based epidemiological observational studies of myasthenia gravis

##### Study population

- Patients included in individual studies were diagnosed with myasthenia gravis according to accepted clinical criteria (based on Osserman criteria)
- Studies examining the AChR +ve serological subtype included patients based on a positive AChR antibody assay – these studies were considered separately in this review.
- Paraclinical investigations (Desmedt RNS criteria, Tensilon testing, SFEMG) were used to support diagnosis
- Final diagnosis was a clinical decision made (in the majority of studies) by an experienced neurologist

# Reporting of search strategy

##### Qualifications of searchers

- Dr Aisling Carr was trained in systematic methods of literature searching as part of her PhD studies, Queens university Belfast
- Search methodology was discussed with Alex McIlroy, Health Sciences librarian, Queens university Belfast, she has a Masters in Library and Information Studies, and has 2 years experience of assisting research/academic staff develop search strategies for systematic reviews

##### Search strategy

- Four medical literature databases searched between Jan 23^rd^ 2008 and March 31^st^ 2008.
- Two proceedings databases were used to search for abstracts of relevant studies
- The following search terms were used as keywords and/or mesh terms:

(“Epidemiological studies” OR “epidemiology” OR “prevalence” OR “incidence” OR “mortality” OR “sex characteristics” OR “sex distribution” OR “age distribution” OR “demographic” OR “demography” OR “population based”) AND ( “myasthenia” OR “myasthenia gravis” OR “anti-acetylcholine receptor antibody” OR “AChR” OR “MuSK” OR “anti-muscle specific kinase antibody” OR “seronegative MG” OR “seronegative myasthenia gravis” OR “congenital myasthenia” OR “congenital myasthenic syndrome”)

*Effort to include all available studies*

- The final list of included studies was discussed with experts in the field (Professor Angela Vincent, Neurosciences Group, John Radcliffe Hospital, Oxford, UK and Dr John McConville, Consultant Neurologist, Ulster Hospital, Belfast)
- Studies in all languages were included
- Authors were not contacted, as adequate information for the performance of this review was available from studies and abstracts.

*Databases and registries searched*

- MEDLINE (Jan week 3 2008 – April week 1 2008) = 176 hits (40 included)
- EMBASE (Jan week 3 2008 – April week 1 2008) = 380 hits (43 included)
- PUBMED (Jan week 3 2008 – April week 1 2008) = 1138 hits (46 included)
- COCHRANE (Jan week 3 2008 – April week 1 2008) = no hits
- ISI proceedings (Sept 2008) = 1981 hits (4 included)
- Zetec proceedings (Sept 2008) = 354 hits (3 included)
- Sources of included papers overlapped well suggesting near complete ascertainment of available studies. This is represented in the Venn diagram below: circles are to scale (number of included papers from that source) but overlapping is estimated

#
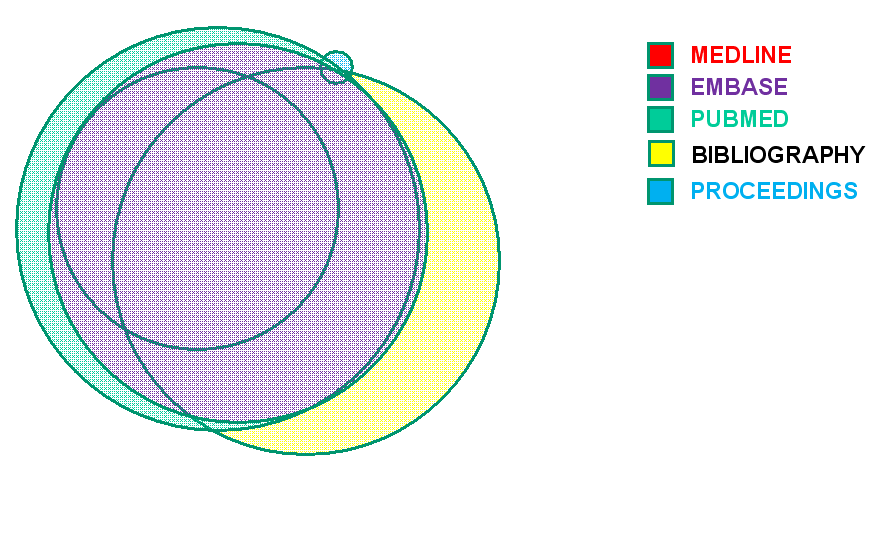


*Search software used*

- PubMed was accessed from the National Library of Medicine (free online)
- Medline and Embase are available on the OVID SP platform
- ISI proceedings is part of the Web of Knowledge
- Mimas at the University of Manchester on behalf of the British Library and JISC provides Zetoc
- Cochrane Library is available on the Wiley Interscience platform.

*Use of hand searching*

- The bibliography of all included studies and those of previous reviews on the subject were examined for further relevant studies

*List of citations located and those excluded*

- See Appendix 1 and 2
- Studies were excluded by title as deemed irrelevant (validated by AC and JMMcC)
- Inclusion and exclusion criteria were applied to abstracts and hits were out ruled in this way (validated by AC and JMMcC)
- 110 full text articles were read by AC and included or excluded according to criteria listed.
- 10 papers (4 excluded and 6 included) were randomly selected and blindly assessed by JMMcC with full agreement.

Inclusion and exclusion criteria

| **Inclusion criteria** | **Exclusion criteria** |
| --- | --- |
| Clearly defined denominator population | Case series |
| Observational epidemiological studies | Undefined denominator population |
| A clear clinical definition of autoimmune myasthenia gravis for inclusion of cases | Previously published data |

- Excluded papers are listed in Appendix 2 with reason for exclusion for all those read in full. Lists of hits excluded by title and/or abstract are listed in full for Medline and Embase searches and the first 200 hits from Pubmed.

##### Non-English articles

- French, Italian and Spanish articles were translated by Dr Aisling Carr
- Articles in Russian and German were translated by fluent speakers and the translations analysed and data extracted by AC
- Adequate data for the purposes of this review was available from the English abstracts of the 2 papers in Chinese and Japanese so these were not translated in full

##### Methods of handling abstracts and unpublished data

- We included proceedings papers and assessed them for eligibility according to our inclusion and exclusion criteria.
- 3 proceedings papers were included. The abstracts provided adequate information for the purposes of this review so the authors were not contacted
- As stated previously, the list of included studies was discussed with experts in the field and deemed to be complete. Beyond this and identification of proceedings papers we did not attempt to identify as yet unpublished data

##### Description of contact with authors

- Contact was not made with authors of the studies. Where data was adequate to calculate rates standardised to world populations this was performed, the standardised rates were not significantly different from crude rates so we did not contact authors of studies where this data was missing. Similarly we analysed incidence by age and sex where this data was provided but did not pursue it in all studies as this was a secondary aim of this review.
- On completion of the review the findings have been discussed with a number of authors of included studies.

# Reporting of methods

Description of relevance or appropriateness of studies assembled for assessing the hypothesis to be tested

- All studies provide observational epidemiological data on patients with confirmed diagnoses of myasthenia gravis
- There is good geographical representation by the studies: all continents are represented except for Australia. (Figure 1)
- Studied period spans from 1950 to 2007
- Study methodology is well documented
- This allows analysis of rates by geography, year and study quality in order to test the hypothesis of the influence of these factors on the observed rates

##### Rationale for selection and coding of data

- Studies were included and excluded as per criteria outlined above
- Study selection was performed by AC and validated by JMMcC with full agreement
- AC extracted data from each study according to a piloted proforma (below). PMcC supervised and validated the piloting process.
- Incidence rates, prevalence rates and mortality rate of MG and 95% confidence intervals were calculated according to the Poisson distribution by hand from crude data extracted; initially by AF and then exact rates were calculated by Stata statistical package by CC.
- Year was documented as final-year of study
- Where available, age and sex incidence figure were also recorded and age/sex specific incidence rates calculated.
- Rates were recorded per million (x10^-6^)

Data extraction proforma

| Title |  | | | | |
| --- | --- | --- | --- | --- | --- |
| **Reference** |  | | | | |
| **Authors** |  | | | | |
| **Country/Area** |  | | | | |
| **Inclusion criteria** |  | | | | |
| **Exclusion criteria** |  | | | | |
| **Sources** |  | | | | |
| **Population studied** |  | | | | |
| **No. of cases** |  | | | | |
| **Crude incidence** |  | **C.I.** |  | **S.E.** |  |
| **Standardised incidence** |  | **C.I.** |  | **S.E.** |  |
| **Crude prevalence** |  | **C.I.** |  | **S.E.** |  |
| **Standardised prevalence** |  | **C.I.** |  | **S.E.** |  |
| **Notes** |  | | | | |

##### Documentation of how data were classified/coded

- After data extraction and calculation of individual study estimates (IR, PR, MR as outlined above) by AC. 10 studies were selected at random (using a random numbers table) and data extraction validated by JMcC (blindly) with full agreement.

*Assessment of confounding*

- Not applicable

##### Assessment of study quality

- No validated quality scales were available for the assessment of epidemiological studies in MG
- Studies were graded High, Intermediate or Low quality according to the criteria listed below. Quality was judged to correlate with completeness of ascertainment as achieved by the use of multiple sources of case ascertainment over an adequate time period.
- AC performed quality assessment of all included studies and JMcC validated a random sample of 10 studies (selected using random numbers table), with full agreement.

### Criteria for assessment of study quality

High

- Cases identified from a variety of sources which represent the whole population (hospital and community)

## AND

- Sufficient population years studied (large population ± long study length ≥5 years)

# Intermediate

- >1 source used to identify cases but whole study population not represented (i.e. hospital sources only)

## AND/OR

- Limited population years studied (either small study population or study length < 5 years)

# Low

- Only 1 source used to identify cases (invalidated assumptions used)

## AND

- Limited population years studied (<5 years + small population)

*Assessment of heterogeneity*

- The hypothesis stated that any observed differences in rates might be related to variation in study methodology/quality more than actual difference in rates of disease in populations over time or by geography. This was tested by looking for a significant reduction in heterogeneity by sub-grouping studies in these ways
- Using Stata statistical package heterogeneity was assessed using Q and I^2^ statistics for IR and PR (the small number of studies providing mortality statistics precluded MR assessment of MR heterogeneity).
- Studies were then grouped according to decade of study and geographical area (Northern Europe, Southern Europe, North America, Central and South America, Africa and Asia) and according to study quality (high, intermediate and low) and heterogeneity calculated for each subgroup

# Description of statistical methods

- Incidence rates, prevalence rates and mortality rates and exact 95% confidence intervals were calculated (from STATA) and plotted in forest plots.
- Where required data was available from the original study (Finland 1968; Sardinia 1986; Western Denmark 1990; Northwest Sardinia 1994; Emilia Romagna, Italy 1994; Croatia 1996; Estonia 1997; Greece (AChR MG) 1997) standardised IR was calculated to 1985 world populations (as the mid year of all studies). There were no marked differences between crude and standardised rates so crude rates are used in this review.
- For incidence and mortality rates, meta-analysis models were applied by using the log incidence\mortality rates and corresponding standard errors. When the counts of cases\deaths were zero, a correction of 0.5 was added to the number of cases\deaths and person-years of follow-up. For prevalence rates, meta-analysis models were applied to the log odds and corresponding standard errors (Sutton AJ, Abrams KR, Jones DR, et al. Methods for meta-analysis in medical research. Chichester, United Kingdom: John Wiley & Sons, 2000.) When the count of cases was zero, a correction of 0.5 was added to the number of cases and the number of non-cases.
- Pooled estimates were calculated using fixed and random effects models from STATA.
- Heterogeneity was formally tested using a chi-squared test and measured using the I^2^ statistic (ref. BMJ. 2003 Sep 6;327(7414):557-60. Measuring inconsistency in meta-analyses. Higgins JP, Thompson SG, Deeks JJ, Altman DG.).
- Meta analysis by the random effects model was used throughout this review due to the marked heterogeneity observed between studies
- Due to massive heterogeneity a pooled statistic could not be calculated except for the High quality group of studies. The reduction in heterogeneity (although still not statistically significant) led us to attempt to calculate a pooled IR statistic for this sub-group using the random affects model. Reasons or excuses for avoiding meta-analysis in forest plots.Ioannidis JP, Patsopoulos NA, Rothstein HR. BMJ. 2008 Jun 21;336(7658):1413-5.
- Meta-regression was used to investigate the association between incidence rate (mortality rate and prevalence rate) and study characteristics such as year and latitude of study. (Ref. How should meta-regression analyses be undertaken and interpreted? Thompson SG, Higgins JP. Stat Med. 2002 Jun 15;21(11):1559-73)
- Funnel plots of incidence rates against standard error of log odds ratio (???or person years of follow-up) were calculated
- An alternative analysis that did not involve any corrections for zero counts of incidence (prevalence and mortality rates) was conducted by using a two-stage method (29) to summarize the incidence rate at the study level and to produce an average rate (and 95 percent confidence interval) based upon the mean and standard deviation of the study rates. This analysis produced similar results and is therefore not shown in this paper.

# Reporting of results

##### Graphic summarising of individual study estimates and pooled estimate

- Forest plots are drawn on the integer scale for ease of interpretation
- Massive heterogeneity precluded the calculation of a meaningful summary statistic/pooled estimate except in the High quality sub-group

##### Table giving descriptive information of all studies included and excluded

- Appendix 1 lists all papers included, with details of relevant data extracted and quality grading
- Appendix 2 lists all excluded papers with reasons for exclusion

##### Results of sensitivity testing

- Temporal, geographical and quality subgroup analysis was performed; effect on heterogeneity is discussed in the paper.

##### Indication of statistical uncertainty of findings

95% confidence intervals (according to the Poisson distribution) for all calculated rates are given

# Reporting of discussion

##### Quantification of publication bias

- Assessed using funnel plots, Begg’s and Eger’s tests (see below)

##### Justification of exclusion

- Papers were excluded on the basis of exclusion criteria listed. We did not systematically exclude any studies on the basis of language or study population size

##### Assessment of quality of included studies

- Studies were graded into High, Intermediate and Low quality according to the criteria listed above. Quality grading was performed by AC and validated by JMcC

# Reporting of conclusions

##### Consideration of alternative explanations for observed results

- See discussion

*Generalisation of these conclusions*

- See discussion

##### Guidelines for future research

- See discussion

##### Disclosure of funding source

- Association Francais contre les Myopathies funded this project. Clinical Research Fellowships from Ulster Hospital Dundonald and Royal Victoria Hospital Belfast, Northern Ireland funded AF as investigator.

Funnel plot of log IR against standard error log odds rate for ALL MG studies and AChR MG studies. The approximate symmetry of both plots suggest the absence of significant publication bias affecting studies included in this review.

0

.1

.2

.3

.4

1

5

10

20

IR per million person years (ALL MG)

Log se rate

IR per million person years (AChR MG)
