# Supplementary material for: A systematic review of population based epidemiological studies in Myasthenia Gravis
Source: BMC Neurol. 2010 Jun 18;10:46. doi: 10.1186/1471-2377-10-46 (PMC2905354; doi:10.1186/1471-2377-10-46)
Supplement: Additional file 2 — All included studies. All included studies are listed with reference (ref), IR: Incidence rate (cases per million person years), PR: Prevalence rate (cases per millions), MR: Mortality rate (deaths due to MG per million person years), 95% C.I., S.E.: standard error and quality grading (1: High, 2: Intermediate, 3: Low). [file 1471-2377-10-46-S2.DOC]

Table (ii)a All MG studies (not defined by serological subtype)

*Quality score: 1=High, 2=Intermediate, 3=Low; Prevalent POP: prevalent population; INCID. CASES= number of incident cases during study period; PREV.CASES= number of prevalent cases on prevalence day; CIR=Crude Incidence Rate (expressed per million population years:x10^-6^); SE=standard error; 95% CI= 95% confidence interval; CPR= Crude Prevalence Rate; CMR=Crude Mortality Rate; Square brackets=bibliography reference*

| YEAR | COUNTRY | *Ref.* | QUALITY | Prevalent POP | Years studied | INCID. CASES | PREV. CASES | CIR (*10^6) | SE (*10^6) | 95% CI (*10^6) | | CPR (*10^6) | SE (*10^6) | 95% CI (*10^6) | | DEAD CASES | CMR (*10^6) | SE (*10^6) | 95% CI (*10^6) | |
| --- | --- | --- | --- | --- | --- | --- | --- | --- | --- | --- | --- | --- | --- | --- | --- | --- | --- | --- | --- | --- |
| 1951 | Norway | [9] | 2 | 3,100,000 | 31,000,000 | 65 | 62 | 2.1 | 0.3 | 1.6 | 2.7 | 20 | 2.5 | 15.3 | 25.6 |  |  |  |  |  |
| 1954 | Halifax, Canada | [4] | 3 | 150,000 | 1,500,000 | 3 | 1 | 2 | 1.2 | 0.4 | 5.8 | 7 | 6.7 | 0.2 | 37.1 |  |  |  |  |  |
| 1954 | Rochester, USA | [10] | 3 | 30,000 | 300,000 | 1 | 1 | 3.3 | 3.3 | 0.1 | 18.6 | 33 | 33 | 0.8 | 185.7 |  |  |  |  |  |
| 1955 | Leeds, UK | [11] | 3 | 500,000 | 7,500,000 | 13 | 18 | 1.7 | 0.5 | 0.9 | 3 | 36 | 8.5 | 21.3 | 56.9 |  |  |  |  |  |
| 1956 | Charleston, USA | [12] | 2 | 188,000 | 1,636,364 | 9 | 6 | 5.5 | 1.8 | 2.5 | 10.4 | 32 | 13 | 11.7 | 69.5 |  |  |  |  |  |
| 1958 | Merseyside, UK | [13] | 3 | 1,500,000 | 16500000 | 33 | 32 | 2 | 0.3 | 1.4 | 2.8 | 21 | 3.8 | 14.6 | 30.1 |  |  |  |  |  |
| 1961 | Norway | [9] | 2 | 3,463,415 | 34,634,146 | 149 | 142 | 4.3 | 0.4 | 3.6 | 5.1 | 41 | 3.4 | 34.5 | 48.3 |  |  |  |  |  |
| 1963 | Iceland | [14] | 2 | 187,000 | 1,724,138 | 7 | 12 | 4.1 | 1.5 | 1.6 | 8.4 | 64 | 18.5 | 33.2 | 118.1 |  |  |  |  |  |
| 1965 | Amsterdam, Holland | [15] | 2 | 860,000 | 4,300,000 | 16 | 48 | 3.7 | 0.9 | 2.1 | 6 | 56 | 8.1 | 41.2 | 74 |  |  |  |  |  |
| 1966 | Fukuoka, Japan | [16] | 2 | 608,000 |  |  | 9 |  |  |  |  | 15 | 4.9 | 6.8 | 28.1 |  |  |  |  |  |
| 1966 | Niigata, Japan | [16] | 2 | 230,000 |  |  | 6 |  |  |  |  | 26 | 10.6 | 9.6 | 56.8 |  |  |  |  |  |
| 1968 | Finland | [17] | 2 | 4,493,392 | 44,933,920 | 102 | 115 | 2.3 | 0.2 | 1.9 | 2.8 | 26 | 2.4 | 21.1 | 30.7 | 26 | 0.58 | 0.11 | 0.4 | 0.85 |
| 1971 | Norway | [9] | 2 | 3,794,872 | 37,948,717 | 148 | 379 | 3.9 | 0.3 | 3.3 | 4.6 | 100 | 5.1 | 90.1 | 110.4 |  |  |  |  |  |
| 1975 | Uruguay | [18] | 2 | 2,700,000 | 27,000,000 | 132 | 170 | 4.9 | 0.4 | 4.1 | 5.8 | 63 | 4.8 | 53.9 | 73.2 |  |  |  |  |  |
| 1982 | Kumamoto, Japan | [19] | 2 | 537,313 |  |  | 36 |  |  |  |  | 67 | 11.2 | 46.9 | 92.8 |  |  |  |  |  |
| 1981 | Norway | [9] | 2 | 4,107,063 | 41,079,630 | 155 | 369 | 3.8 | 0.3 | 3.2 | 4.4 | 90 | 4.7 | 80.9 | 99.5 |  |  |  |  |  |
| 1984 | Virginia, USA | [20] | 1 | 555,851 | 8,021,985 | 73 | 79 | 9.1 | 1.1 | 7.1 | 11.4 | 142 | 16 | 112.5 | 177.1 |  |  |  |  |  |
| 1984 | Georgia, Russia | [21] | 3 | 5,000,000 |  |  | 161 |  |  |  |  | 32 | 2.5 | 27.4 | 37.6 |  |  |  |  |  |
| 1985 | Karachi, Pakistan | [22] | 2 | 1,038,462 |  |  | 27 |  |  |  |  | 26 | 5 | 17.1 | 37.8 |  |  |  |  |  |
| 1986 | Sardinia, Italy | [23] | 2 | 2,444,444 | 43,999,989 | 110 | 110 | 2.5 | 0.24 | 2.1 | 3.03 | 45 | 4.3 | 37 | 54.2 | 19 | 0.43 | 0.1 | 0.27 | 0.68 |
| 1986 | Benghazi, Libya | [24] | 2 | 519,000 | 2,076,000 | 9 |  | 4.3 | 1.5 | 2 | 8.2 |  |  |  |  |  |  |  |  |  |
| 1987 | Bologna, Italy | [25] | 1 | 914,463 |  |  | 66 |  |  |  |  | 72 | 8.9 | 55.8 | 91.8 |  |  |  |  |  |
| 1987 | Hong Kong, China | [26] | 2 | 4,860,000 | 50,160,000 | 202 | 260 | 4 | 0.3 | 3.5 | 4.6 | 54 | 3.3 | 47.2 | 60.4 | 7 | 0.14 | 0.05 | 0.07 | 0.29 |
| 1987 | Viborg, Denmark | [27] | 2 | 230,760 | 3,461,400 | 36 | 29 | 10.4 | 1.7 | 7.3 | 14.4 | 126 | 23.3 | 84.2 | 180.5 |  |  |  |  |  |
| 1988 | Ferrara, Italy | [25] | 1 | 370,374 |  |  | 39 |  |  |  |  | 105 | 16.9 | 74.9 | 143.9 |  |  |  |  |  |
| 1988 | Eastern Demark | [28] | 2 | 2,298,701 | 41,363,636 | 182 | 177 | 4.4 | 0.3 | 3.8 | 5.1 | 77 | 5.8 | 66.1 | 89.2 | 37 | 0.89 | 0.15 | 0.64 | 1.23 |
| 1989 | Western Denmark | [29] | 2 | 2,800,000 | 70,000,000 |  | 290 |  |  |  |  |  |  |  |  | 27 | 0.39 | 0.07 | 0.27 | 0.57 |
| 1990 | Trento, Italy | [30] | 1 | 446,914 | 4,469,140 | 33 | 37 | 7.4 | 1.3 | 5.1 | 10.4 | 83 | 13.6 | 58.3 | 114.1 |  |  |  |  |  |
| 1990 | Western Denmark | [31] | 2 | 2,800,000 | 28,011,150 | 140 | 220 | 5 | 0.4 | 4.2 | 5.9 | 79 | 5.3 | 68.5 | 89.7 |  |  |  |  |  |
| 1991 | Cuba | [32] | 2 | 5,782,309 | 28,911,545 | 131 | 169 | 4.5 | 0.4 | 3.8 | 5.4 | 30 | 2.3 | 25 | 34 |  |  |  |  |  |
| 1991 | Krasnodar, Russia | [33] | 3 | 655,738 |  |  | 23 |  |  |  |  | 35 | 7.3 | 22.2 | 52.6 |  |  |  |  |  |
| 1992 | Belgrade, Yugoslavia | [34] | 1 | 1,530,864 | 15,308,640 | 124 | 117 | 8.1 | 0.7 | 6.7 | 9.7 | 76 | 7.1 | 63.2 | 91.6 | 1 | 0.06 | 0.06 | 0 | 0.43 |
| 1994 | NW Sardinia, Italy | [35] | 1 | 268,926 | 3,179,472 | 27 | 30 | 8.5 | 1.6 | 5.6 | 12.4 | 112 | 20.4 | 86.8 | 159.2 |  |  |  |  |  |
| 1994 | Reggio Emilia, Italy | [36] | 1 | 427,493 | 6,260,390 | 49 | 50 | 7.8 | 1.1 | 5.8 | 10.3 | 118 | 16.5 | 86.7 | 154.2 | 4 | 0.64 | 0.32 | 0.24 | 1.7 |
| 1994 | Emilia Romagna, Italy | [37] | 1 | 2,924,710 | 5,849,420 | 86 |  | 14.7 | 1.6 | 11.8 | 18.2 |  |  |  |  |  |  |  |  |  |
| 1994 | Cyprus | [38] | 3 | 600,000 | 6,000,000 | 72 | 105 | 12 | 1.4 | 9.4 | 15.1 | 175 | 17.1 | 143.1 | 211.8 |  |  |  |  |  |
| 1995 | London, UK | [39] | 2 | 100,230 | 100,230 | 3 |  | 30 | 17.3 | 6.2 | 87.5 |  |  |  |  |  |  |  |  |  |
| 1995 | Dutch Antilles | [40] | 3 | 229,800 | 3,232,815 | 15 | 15 | 4.6 | 1.2 | 2.6 | 7.7 | 65 | 16.9 | 36.5 | 107.7 |  |  |  |  |  |
| 1996 | La Palma, Spain | [41] | 2 | 81,507 |  |  | 7 |  |  |  |  | 86 | 32.5 | 34.5 | 176.9 |  |  |  |  |  |
| 1996 | Gorski kotar, Croatia | [42] | 2 | 313,599 | 6,585,579 | 43 | 32 | 6.5 | 1 | 4.7 | 8.8 | 102 | 18 | 69.8 | 144 |  |  |  |  |  |
| 1997 | CambridgeshireEngland | [43] | 1 | 684,000 | 3,420,000 | 38 | 100 | 11.1 | 1.8 | 7.9 | 15.3 | 146 | 14.6 | 119 | 177.8 |  |  |  |  |  |
| 1997 | Assuit, Egypt | [44] | 2 | 50,000 |  |  | 5 |  |  |  |  | 100 | 44.7 | 32.5 | 233.4 |  |  |  |  |  |
| 1997 | Estonia | [45] | 2 | 1,462,130 | 39,214,890 | 162 | 208 | 4.1 | 0.3 | 3.5 | 4.8 | 142 | 9.9 | 123.6 | 163 |  |  |  |  |  |
| 1998 | Stockholm, Sweden | [46] | 2 | 1,783,428 |  |  | 251 |  |  |  |  | 141 | 8.9 | 123.9 | 159.3 |  |  |  |  |  |
| 1998 | Dar Es Salaam, Tanzania | [47] | 2 | 2,300,000 | 23,000,000 | 47 |  | 2 | 0.3 | 1.5 | 2.7 |  |  |  |  |  |  |  |  |  |
| 2000 | Antioqua, Columbia | [48] | 2 | 5,300,000 |  |  | 147 |  |  |  |  | 28 | 2.3 | 23.4 | 32.6 |  |  |  |  |  |
| 2000 | Southern Holland | [49] | 2 | 1,725,317 | 17,253,170 | 112 | 189 | 6.5 | 0.6 | 5.4 | 7.8 | 110 | 8 | 94.5 | 126.3 |  |  |  |  |  |
| 2000 | Nagano, Japan | [50] | 2 | 2,350,000 | 47,000,000 | 213 |  | 4.5 | 0.3 | 3.9 | 5.2 |  |  |  |  |  |  |  |  |  |
| 2000 | Barcelona, Spain | [51] | 3 | 122,923 | 1,229,230 | 26 |  | 21.3 | 4.2 | 13.9 | 31.2 |  |  |  |  |  |  |  |  |  |
| 2001 | Denmark | [52] | 3 | 5,472,032 |  |  | 977 |  |  |  |  | 179 | 5.7 | 167.5 | 190.1 |  |  |  |  |  |
| 2001 | Moscow, USSR | [53] | 2 | 12,000,000 |  |  | 1076 |  |  |  |  | 90 | 2.7 | 84.4 | 95.2 |  |  |  |  |  |
| 2004 | Southern Holland | [54] | 2 | 1,778,564 | 23,926,703 | 154 | 253 | 6.4 | 0.5 | 5.5 | 7.5 | 142 | 8.9 | 125.3 | 160.9 |  |  |  |  |  |

Table (ii)b All included AChR-MG studies

|  | YEAR | COUNTRY | QUALITY | Prevalent POP | Years studied | INCID. CASES | PREV. CASES | CIR (*10^6) | SE (*10^6) | CI (*10^6) | | CPR (*10^6) | SE (*10^6) | CI (*10^6) | | DEAD CASES | CMR (*10^6) | SE (*10^6) | CI (*10^6) | |
| --- | --- | --- | --- | --- | --- | --- | --- | --- | --- | --- | --- | --- | --- | --- | --- | --- | --- | --- | --- | --- |
| [55] | 1988 | Eastern Denmark | 2 | 2,298,701 | 41,363,636 |  | 376 |  |  |  |  | 163.5 | 8.4 | 147 | 180 |  |  |  |  |  |
| [56] | 1990 | British Colombia, Canada | 2 | 4,000,000 | 52,000,000 | 614 |  | 11.8 | 0.5 | 10.9 | 12.8 |  |  |  |  |  |  |  |  |  |
| [57] | 1997 | Greece | 3 | 10,475,873 | 152,713,695 | 733 | 740 | 4.8 | 0.2 | 4.5 | 5.2 | 70.6 | 2.6 | 65.5 | 75.7 | 66 | 0.43 | 0.05 | 0.34 | 0.55 |
| [58] | 1998 | UK | 3 | 58,722,222 | 176,166,666 | 3171 |  | 18 | 0.3 | 17.4 | 18.6 |  |  |  |  |  |  |  |  |  |
| [50] | 2000 | Nagano, Japan | 2 | 2,300,000 | 41,400,000 | 178 |  | 4.3 | 0.3 | 3.7 | 5 |  |  |  |  |  |  |  |  |  |
| [59] | 2001 | Tayside, Scotland | 3 | 600,000 | 4,200,000 | 44 |  | 10.5 | 1.6 | 7.6 | 14.1 |  |  |  |  |  |  |  |  |  |
| [60] | 2001 | Norway | 2 | 5,392,857 | 64,714,284 | 453 |  | 7 | 0.3 | 6.4 | 7.7 |  |  |  |  |  |  |  |  |  |
| [54] | 2004 | Southern Holland | 2 | 1,778,564 | 23,926,703 | 111 | 160 | 4.6 | 0.4 | 3.8 | 5.6 | 89.9 | 7.1 | 76 | 103.8 |  |  |  |  |  |
| [61] | 2005 | Cape Town, South Africa | 2 | 2,893,251 | 5,786,502 | 65 |  | 11.2 | 1.4 | 8.7 | 14.3 |  |  |  |  |  |  |  |  |  |

Table (ii)cAll included MuSK studies

|  | YEAR | COUNTRY | QUALITY | Prevalent POP | Years studied | INCID. CASES | PREV. CASES | CIR (*10^6) | SE (*10^6) | CI (*10^6) | | CPR (*10^6) | SE (*10^6) | CI (*10^6) | | CMR due (*10^6) | SE (*10^6) | CI (*10^6) | |
| --- | --- | --- | --- | --- | --- | --- | --- | --- | --- | --- | --- | --- | --- | --- | --- | --- | --- | --- | --- |
| [54] | 2004 | Southern Holland | 2 | 1,778,564 | 23,926,703 | 4 | 5 | 0.17 | 0.08 | 0.07 | 0.41 | 1.9 | 0.34 | 1.22 | 2.59 |  |  |  |  |
| [54] | 2004 | Holland | 2 | 16,258,032 | 218,054,242 | 22 | 31 | 0.1 | 0.02 | 0.07 | 0.152 |  |  |  |  |  |  |  |  |
| [62] | 2006 | Greece | 2 | 11293282 | 1072866179 | 33 | 33 | 0.32 |  |  |  | 2,92 | 0.5 | 1.92 | 3.92 |  |  |  |  |
