# Supplementary material for: A systematic review of population based epidemiological studies in Myasthenia Gravis
Source: BMC Neurol. 2010 Jun 18;10:46. doi: 10.1186/1471-2377-10-46 (PMC2905354; doi:10.1186/1471-2377-10-46)
Supplement: Additional file 3 — All excluded studies. Table (iii)a List of studies excluded on examination of full text (or abstract only from proceedings papers) with reason for exclusion. Proceedings papers (P) Table (iii)b List of studies excluded on the basis of title and/or abstract from initial database searches (Medline, EMBASE and first 250 hits from Pubmed only). [file 1471-2377-10-46-S3.DOC]

**Additional file 3 Excluded studies and reasons for exclusion**

Table (ii)a List of studies excluded on examination of full text (or abstract only from proceedings papers) with reason for exclusion.

Proceedings papers (P)

| Year | Study area | **Reason for exclusion** | *Reference* |
| --- | --- | --- | --- |
| 1950 (P) | Boston, USA | - Case series - Denominator population not defined | Trans. Am. Neurol. Assoc. 1953:3(78^th^ meeting) 153-5 |
| 1952 (P) | USA | - Case series - Previously published data | Trans Am Neurol Assoc 1953:3(78^th^ meeting) |
| 1955 | France | - Case series - Denominator population not defined | Sem. Hop. Paris. 14/02/1957 |
| 1962 | USA | - Case series - Denominator population not defined | Advances in Neurology 1978 (19)545-551 |
| 1967 | Senegal | - Case series - Denominator population not defined | Bull. Soc. Med. Afr. Noire. Lanque fr. 1969tXIV (2) 269-273 |
| 1969 | Kenya | - Case series - Denominator population not defined | Tropical Med. 1969, 73(5):680-1 |
| 1969 | Finland | - Denominator population not defined | Acta neurol. Scandinav. 56, 356-388, 1977 |
| 1973 | N/A | - Review - Previously published data | Epidemiology of Neurolgic and Sense organs. 1973:144-52 |
| 1974 | Ethiopia | - Case series - Previously published data | Trop. Geogr. Med., 32(1980)231-236 |
| 1975 | Japan | - Denominator population not defined | International Journal if Neurology 1980(14)1:87-96 |
| 1977 | Finalnd | - Review - Previously published data | Journal of Neurological Sciences, 1989, 89: 37-48 |
| 1978 | N/A | - Review - Previously published data | Advances in Neurology. Vol.19. 1978 |
| 1979 (P) | Asia | - Review - Previously published data | International Symposium on Myasthenia Gravis, 1979 |
| 1980 | Norway | - Review - Previously published data | Tidsskr Nor Loegeforen 1992:112:1574-8 |
| 1980 | Spain | - Denominator population not defined | Anales Espanoles de Pediatria, 1988:29 (Suppl. 33) 74-77 |
| 1980 | N/A | - Review - Previously published data | Clin. Neurol. Neurosurg. 1981. Vol.83-3 |
| 1982 | Kumamoto, Japan | - Previously published data | Clin. Neurol, 23: 838-841, 1983 |
| 1983 | Kumamoto, Japan | - Previously published data | Neuroepidemiology 6;120-129 (1987) |
| 1984 | Norway | - Previously published data | Acta Neurol Scand:80;4:290-95(1989) |
| 1987 | N/A | - Review - Previously published data | Monogr. Allergy 1987:21:246-251 |
| 1993 | Eastern Denmark | - Previously published data | Neurology 2002; 59;92-98 |
| 1993 | Iran | - Congenital myasthenic syndromes, not autoimmune MG | American Journal of Medical Genetics (1995) 58:32-37 |
| 1994 | Yugoslavia | - Previously published data | Sprski Archiv Za Celokupno Lekaistve 2005:22(7):453-456 |
| 1994 | N/A | - Review - Previously published data | Neurologic Clinics of North America 12(2) 1994:263-271 |
| 1996 | USA | - Review - Previously published data | Clinical Immunology and Immunopathology 1997; 84(3)223-234 |
| 1996 | N/A | - Review - Previously published data | Neurology 1996;47;1233-1238 |
| 1997 | Greece | - Previously published data | Neurology 54:1202-3(2000) |
| 1997 | Estonia | - Previously published data | Eurpoean Journal of Neurology 2008. 15:246-252 |
| 1999 | N/A | - Review - Previously published data | Arch Neurol. 1999(56) 25-27 |
| 1999 | Mozambique | - Denominator population not defined. - MG not defined clinically | Rev Neurol 2000;30(12): 1135-1140 |
| 2000 | Minnesota, USA | - Denominator population not defined | Muscle Nerve 36:651-658, 2007 |
| 2000 | Nagano, Japan | - Previously published data | Internal Medicine 44:572-577, 2005 |
| 2001 | Norway | - Denominator population not defined | JNNP 2006; 77:203-207 |
| 2001 | Hong Kong | - Population limited to under 19 years | J Child Neurol. 2003:18:217-219 |
| 2001 (P) | Milan, Italy | - Case series - Denominator population not defined | Ann. N.Y. Acad. Sci. 998:413-423 (2003) |
| 2001 | Lithuania | - Denominator population not defined | Medicina (2002) Vol.38, 6:611-616 |
| 2001 (P) | Argentina | - Previous presented data | Journal of Neuology Vol. 254: 153, Suppl 3 (2007) |
| 2003 | USA | - Previously published data | Autoimmunity Reviews 2(2003) 119-125 |
| 2003 (P) | N/A | - Review - Previously published data | Ann.N.Y.Acad.Sci. 998:407-412 (2003) |
| 2003 (P) | N/A | - Review | Ann.N.Y.Acad.Sci. 998: 413-421 (2003) |
| 2003 (P) | N/A | - Review | Ann.N.Y.Acad.Sci. 998: 424-231 (2003) |
| 2004 (P) | Multi-centre | - Case series | Ann. Neurol. 56:S63. Suppl. 8 (2004) |
| 2004 (P) | N/A | - Review | Journal of Neuroimmunology. Vol. 154, SpIss:1:110, Abstract: 365 (2004) |
| 2005 (P) | Korea | - Case series, denominator population not defined | Journal of the Neurological Sciences. Vol. 238: S175. Suppl.1 (2005) |
| 2005 | HuBei province, China | - Case series, denominator population not defined | JNNP 2007; 78; 386-390 |
| 2005 (P) | Poland | - Case series, denominator population not defined | Europ. Journal Neurology. Vol14 :262, Suppl.1 (2008) |
| 2006 (P) | Worldwide | - Case series | Journal of Neuroimmunology . Vol.178:233, Suppl.1 (2006) |
| 2006 (P) | N/A | - Review - Previously published data | Journal of Neurology 2006 253(Suppl. 5), V/2-V/8 |
| 2007 | Wales | - Previously published data | Neuroepidemiology 2007;28:65-78 |
| 2007 (P) | N/A | - Review | Ann. N.Y.Acad.Sci. 1132: 71-75 (2008) |
| 2007 (P) | N/A | - Review | Ann.N.Y.Acac.Sci. 1132: 238-242 (2008) |
| 2008 (P) | USA | - Case series, denominator population not defined | Ann. Neurol. 64:S6-S7. Suppl.12 (2008) |

Table (iii)b List of studies excluded on the basis of title and/or abstract from initial database searches

MEDLINE

Evoli A. Minicuci GM. Vitaliani R. Battaglia A. Della Marca G. Lauriola L. Fattorossi A. Paraneoplastic diseases associated with thymoma. [Journal Article] Journal of Neurology. 254(6):756-62, 2007 Jun.
UI: 17325820

Authors Full Name
Evoli, Amelia. Minicuci, Giacomo Maria. Vitaliani, Roberta. Battaglia, Alessandra. Della Marca, Giacomo. Lauriola, Libero. Fattorossi, Andrea.

Antoine JC. Camdessanche JP. [Paraneoplastic neurological syndromes]. [Review] [25 refs] [French] [Comparative Study. English Abstract. Journal Article. Review] Presse Medicale. 36(10 Pt 2):1418-26, 2007 Oct.
UI: 17399944

Authors Full Name
Antoine, Jean-Christophe. Camdessanche, Jean-Philippe.

Deymeer F. Gungor-Tuncer O. Yilmaz V. Parman Y. Serdaroglu P. Ozdemir C. Vincent A. Saruhan-Direskeneli G. Clinical comparison of anti-MuSK- vs anti-AChR-positive and seronegative myasthenia gravis. [Comparative Study. Journal Article. Research Support, Non-U.S. Gov't] Neurology. 68(8):609-11, 2007 Feb 20.
UI: 17310034

Authors Full Name
Deymeer, F. Gungor-Tuncer, O. Yilmaz, V. Parman, Y. Serdaroglu, P. Ozdemir, C. Vincent, A. Saruhan-Direskeneli, G

Ashraf VV. Taly AB. Veerendrakumar M. Rao S. Myasthenia gravis in children: a longitudinal study. [Comparative Study. Journal Article] Acta Neurologica Scandinavica. 114(2):119-23, 2006 Aug.
UI: 16867035

Authors Full Name
Ashraf, V V. Taly, A B. Veerendrakumar, M. Rao, S.

Niks EH. Kuks JB. Roep BO. Haasnoot GW. Verduijn W. Ballieux BE. De Baets MH. Vincent A. Verschuuren JJ. Strong association of MuSK antibody-positive myasthenia gravis and HLA-DR14-DQ5. [Clinical Trial. Journal Article. Multicenter Study. Research Support, Non-U.S. Gov't] Neurology. 66(11):1772-4, 2006 Jun 13.
UI: 16769963

Authors Full Name
Niks, E H. Kuks, J B M. Roep, B O. Haasnoot, G W. Verduijn, W. Ballieux, B E P B. De Baets, M H. Vincent, A. Verschuuren, J J G M.

Jitpimolmard S. Taimkao S. Chotmongkol V. Sawanyawisuth K. Vincent A. Newsom-Davis J. Acetylcholine receptor antibody in Thai generalized myasthenia gravis patients. [Journal Article] Journal of the Medical Association of Thailand. 89(1):68-71, 2006 Jan.
UI: 16583584

Authors Full Name
Jitpimolmard, Suthipun. Taimkao, Somsak. Chotmongkol, Verajit. Sawanyawisuth, Kittisak. Vincent, Angela. Newsom-Davis, John.

Sthoeger Z. Neiman A. Elbirt D. Zinger H. Magen E. Burstein R. Eitan S. Abarbanel J. Mozes E. High prevalence of systemic lupus erythematosus in 78 myasthenia gravis patients: a clinical and serologic study. [Journal Article] American Journal of the Medical Sciences. 331(1):4-9, 2006 Jan.
UI: 16415656

Authors Full Name
Sthoeger, Zev. Neiman, Alexander. Elbirt, Daniel. Zinger, Hiedy. Magen, Eli. Burstein, Rimona. Eitan, Sara. Abarbanel, Jakov. Mozes, Edna

Lavrnic D. Losen M. Vujic A. De Baets M. Hajdukovic LJ. Stojanovic V. Trikic R. Djukic P. Apostolski S. The features of myasthenia gravis with autoantibodies to MuSK. [Journal Article] Journal of Neurology, Neurosurgery & Psychiatry. 76(8):1099-102, 2005 Aug.
UI: 16024887

Authors Full Name
Lavrnic, D. Losen, M. Vujic, A. De Baets, M. Hajdukovic, L J. Stojanovic, V. Trikic, R. Djukic, P. Apostolski, S.

Romi F. Aarli JA. Gilhus NE. Seronegative myasthenia gravis: disease severity and prognosis. [Clinical Trial. Comparative Study. Journal Article] European Journal of Neurology. 12(6):413-8, 2005 Jun.
UI: 15885043

Authors Full Name
Romi, F. Aarli, J A. Gilhus, N E.

Morar B. Gresham D. Angelicheva D. Tournev I. Gooding R. Guergueltcheva V. Schmidt C. Abicht A. Lochmuller H. Tordai A. Kalmar L. Nagy M. Karcagi V. Jeanpierre M. Herczegfalvi A. Beeson D. Venkataraman V. Warwick Carter K. Reeve J. de Pablo R. Kucinskas V. Kalaydjieva L. Mutation history of the roma/gypsies. [Journal Article. Research Support, Non-U.S. Gov't] American Journal of Human Genetics. 75(4):596-609, 2004 Oct.
UI: 15322984

Isbister CM. Mackenzie PJ. Anderson D. Wade NK. Oger J. Co-occurrence of multiple sclerosis and myasthenia gravis in British Columbia. [Journal Article. Research Support, Non-U.S. Gov't] Multiple Sclerosis. 9(6):550-3, 2003 Dec.
UI: 14664466

Posner JB. Immunology of paraneoplastic syndromes: overview. [Review] [34 refs] [Journal Article. Review] Annals of the New York Academy of Sciences. 998:178-86, 2003 Sep.
UI: 14592873

Authors Full Name
Posner, Jerome B.

Evoli A. Tonali PA. Padua L. Monaco ML. Scuderi F. Batocchi AP. Marino M. Bartoccioni E. Clinical correlates with anti-MuSK antibodies in generalized seronegative myasthenia gravis. [Case Reports. Journal Article] Brain. 126(Pt 10):2304-11, 2003 Oct.
UI: 12821509

Kittiwatanapaisan W. Gauthier DK. Williams AM. Oh SJ. Fatigue in Myasthenia Gravis patients. [Journal Article. Research Support, Non-U.S. Gov't. Validation Studies] Journal of Neuroscience Nursing. 35(2):87-93, 106, 2003 Apr.
UI: 12795035

Raksadawan N. Kankirawatana P. Balankura K. Prateepratana P. Sangruchi T. Atchaneeyasakul LO. Childhood onset myasthenia gravis. [Journal Article] Journal of the Medical Association of Thailand. 85 Suppl 2:S769-77, 2002 Aug.
UI: 12403259

Haliloglu G. Anlar B. Aysun S. Topcu M. Topaloglu H. Turanli G. Yalnizoglu D. Gender prevalence in childhood multiple sclerosis and myasthenia gravis. [Journal Article] Journal of Child Neurology. 17(5):390-2, 2002 May.
UI: 12150589

Vega-Garces WJ. Aguilera-Pacheco O. Nunez-Gil H. Luis-Gonzalez S. [Myasthenia gravis in patients over 50 years of age]. [Spanish] [English Abstract. Journal Article] Revista de Neurologia. 28(10):948-51, 1999 May 16-31.
UI: 10416228

Frago G. Gracia F. Chang E. Andrade Alegre R. [Myasthenia gravis in Santo Tomas Hospital 1990-1997]. [Spanish] [English Abstract. Journal Article] Revista Medica de Panama. 23(2):15-9, 1998 Sep.
UI: 11214554

Authors Full Name
Frago, G. Gracia, F. Chang, E. Andrade Alegre, R.

Anlar B. Ozdirim E. Renda Y. Yalaz K. Aysun S. Topcu M. Topaloglu H. Myasthenia gravis in childhood. [Journal Article] Acta Paediatrica. 85(7):838-42, 1996 Jul.
UI: 8819551

el-Zunni S. Prakash PS. Saitti M. Busnaina IA. Myasthenia gravis (MG): a preliminary report. [Case Reports. Journal Article] Central African Journal of Medicine. 42(3):77-80, 1996 Mar.
UI: 8653775

Rey RD. Buzzi AE. Astudillo MA. Diaz G. Losavio A. Muchnik S. Sanz OP. Sica RE. [Diagnosis and treatment of myasthenia gravis. Experience 1974-1992]. [Spanish] [Comparative Study. English Abstract. Journal Article] Medicina. 55(1):11-20, 1995.
UI: 7565029

Christensen PB. Jensen TS. Tsiropoulos I. Sorensen T. Kjaer M. Hojer-Pedersen E. Rasmussen MJ. Lehfeldt E. Associated autoimmune diseases in myasthenia gravis. A population-based study.[see comment]. [Journal Article] Acta Neurologica Scandinavica. 91(3):192-5, 1995 Mar.
UI: 7793234

Tola MR. Caniatti LM. Casetta I. Granieri E. Conighi C. Quatrale R. Monetti VC. Paolino E. Govoni V. Pascarella R. et al. Immunogenetic heterogeneity and associated autoimmune disorders in myasthenia gravis: a population-based survey in the province of Ferrara, northern Italy. [Journal Article. Research Support, Non-U.S. Gov't] Acta Neurologica Scandinavica. 90(5):318-23, 1994 Nov.
UI: 7887131

March GA Jr. Johnson LN. Ocular myasthenia gravis. [Review] [33 refs] [Journal Article. Research Support, Non-U.S. Gov't. Review] Journal of the National Medical Association. 85(9):681-4, 1993 Sep.
UI: 8120928

Authors Full Name
March, G A Jr. Johnson, L N.

Aarli JA. Gilhus NE. Matre R. Myasthenia gravis with thymoma is not associated with an increased incidence of non-muscle autoimmune disorders. [Comparative Study. Journal Article] Autoimmunity. 11(3):159-62, 1992.
UI: 1571478

Authors Full Name
Aarli, J A. Gilhus, N E. Matre, R

Mantegazza R. Beghi E. Pareyson D. Antozzi C. Peluchetti D. Sghirlanzoni A. Cosi V. Lombardi M. Piccolo G. Tonali P. et al. A multicentre follow-up study of 1152 patients with myasthenia gravis in Italy. [Clinical Trial. Journal Article. Multicenter Study. Research Support, Non-U.S. Gov't] Journal of Neurology. 237(6):339-44, 1990 Oct.
UI: 2277266

Authors Full Name
Mantegazza, R. Beghi, E. Pareyson, D. Antozzi, C. Peluchetti, D. Sghirlanzoni, A. Cosi, V. Lombardi, M. Piccolo, G. Tonali, P. et al.

Perlo VP. Poskanzer DC. Schwab RS. Viets HR. Osserman KE. Genkins G. Myasthenia gravis: evaluation of treatment in 1,355 patients. [Journal Article] Neurology. 16(5):431-9, 1966 May.
UI: 5949058

Authors Full Name
Perlo, V P. Poskanzer, D C. Schwab, R S. Viets, H R. Osserman, K E. Genkins, G.

Kanazawa M. Shimohata T. Tanaka K. Nishizawa M. Clinical features of patients with myasthenia gravis associated with autoimmune diseases. [Journal Article] European Journal of Neurology. 14(12):1403-4, 2007 Dec.
UI: 17941854

Authors Full Name
Kanazawa, M. Shimohata, T. Tanaka, K. Nishizawa, M.

(no citing articles)

Tsinzerling N. Lefvert AK. Matell G. Pirskanen-Matell R. Myasthenia gravis: a long term follow-up study of Swedish patients with specific reference to thymic histology. [Journal Article] Journal of Neurology, Neurosurgery & Psychiatry. 78(10):1109-12, 2007 Oct.
UI: 17353257

Authors Full Name
Tsinzerling, Natalie. Lefvert, Ann-Kari. Matell, Georg. Pirskanen-Matell, Ritva.

(no citing articles)

Barbaud A. Carlander B. Pages M. [Late onset forms of myasthenia gravis. Comparison with early-onset myasthenia gravis]. [French] [English Abstract. Journal Article] Revue Neurologique. 162(10):990-6, 2006 Oct.
UI: 17028567

Authors Full Name
Barbaud, A. Carlander, B. Pages, M.

(no citing articles)

Toth C. McDonald D. Oger J. Brownell K. Acetylcholine receptor antibodies in myasthenia gravis are associated with greater risk of diabetes and thyroid disease. [Comparative Study. Journal Article] Acta Neurologica Scandinavica. 114(2):124-32, 2006 Aug.
UI: 16867036

Authors Full Name
Toth, C. McDonald, D. Oger, J. Brownell, K.

(no relevant citing articles)

Aarli JA. Romi F. Skeie GO. Gilhus NE. Myasthenia gravis in individuals over 40. [Comparative Study. Journal Article] Annals of the New York Academy of Sciences. 998:424-31, 2003 Sep.
UI: 14592910

Authors Full Name
Aarli, Johan A. Romi, Frederik. Skeie, Geir Olve. Gilhus, Nils Erik.

Kollar S. Dioszeghy P. Hallay J. Kovacs G. [Surgical aspects in the treatment of myasthenia gravis]. [Hungarian] [English Abstract. Journal Article] Magyar Sebeszet. 55(4):233-6, 2002 Aug.
UI: 12236078

Somnier FE. Engel PJ. The occurrence of anti-titin antibodies and thymomas: a population survey of MG 1970-1999. [Journal Article. Research Support, Non-U.S. Gov't] Neurology. 59(1):92-8, 2002 Jul 9.
UI: 12105313

Authors Full Name
Somnier, Finn E. Engel, Peter J H.

Bol P. [Myasthenia gravis]. [Review] [4 refs] [Dutch] [Journal Article. Review] Nederlands Tijdschrift voor Tandheelkunde. 108(10):416-7, 2001 Oct.
UI: 11680078

Authors Full Name

Schon F. Drayson M. Thompson RA. Myasthenia gravis and elderly people. [Journal Article] Age & Ageing. 25(1):56-8, 1996 Jan.
UI: 8670530

Authors Full Name
Schon, F. Drayson, M. Thompson, R A

Andrews PI. Massey JM. Howard JF Jr. Sanders DB. Race, sex, and puberty influence onset, severity, and outcome in juvenile myasthenia gravis. [Journal Article] Neurology. 44(7):1208-14, 1994 Jul.
UI: 8035917

Authors Full Name
Andrews, P I. Massey, J M. Howard, J F Jr. Sanders, D B.

Thompson PN. van der Werf JH. Heesterbeek JA. van Arendonk JA. The CHRNE 470del20 mutation causing congenital myasthenic syndrome in South African Brahman cattle: prevalence, origin, and association with performance traits. [Journal Article] Journal of Animal Science. 85(3):604-9, 2007 Mar.
UI: 17121978

Authors Full Name
Thompson, P N. van der Werf, J H J. Heesterbeek, J A P. van Arendonk, J A M.

Przybylik-Mazurek E. Kotlinowska B. Kasztelnik M. Stefanska A. Huszno B. [Autoimmunological and allergic disorders with Hashimoto and Graves disease]. [Polish] [English Abstract. Journal Article] Przeglad Lekarski. 63(9):719-22, 2006.
UI: 17479856

Authors Full Name
Przybylik-Mazurek, Elwira. Kotlinowska, Barbara. Kasztelnik, Magdalena. Stefanska, Agnieszka. Huszno, Bohdan.

Rawoot A. Little F. Heckmann JM. Risk of malignancy in myasthenia gravis patients exposed to azathioprine therapy for a median period of 3 years. [Letter. Research Support, Non-U.S. Gov't] South African Medical Journal. Suid-Afrikaanse Tydskrif Vir Geneeskunde. 96(12):1249-51, 2006 Dec.
UI: 17252153

Authors Full Name
Rawoot, A. Little, F. Heckmann, J M.

Nicolle MW. Rask S. Koopman WJ. George CF. Adams J. Wiebe S. Sleep apnea in patients with myasthenia gravis. [Comparative Study. Journal Article. Research Support, Non-U.S. Gov't] Neurology. 67(1):140-2, 2006 Jul 11.
UI: 16832094

Authors Full Name
Nicolle, M W. Rask, S. Koopman, W J. George, C F P. Adams, J. Wiebe, S.

Brann JH. Fadool DA. Vomeronasal sensory neurons from Sternotherus odoratus (stinkpot/musk turtle) respond to chemosignals via the phospholipase C system. [Journal Article. Research Support, N.I.H., Extramural] Journal of Experimental Biology. 209(Pt 10):1914-27, 2006 May.
UI: 16651557

Authors Full Name
Brann, Jessica H. Fadool, Debra A.

Durand F. Camdessanche JP. Jomir L. Antoine JC. Cathebras P. [Myasthenia in elderly patients: a series of 23 cases]. [French] [English Abstract. Journal Article] Revue de Medecine Interne. 26(12):924-30, 2005 Dec.
UI: 16229927

Authors Full Name
Durand, F. Camdessanche, J-P. Jomir, L. Antoine, J-C. Cathebras, P.

Chuang WY. Strobel P. Gold R. Nix W. Schalke B. Kiefer R. Opitz A. Klinker E. Muller-Hermelink HK. Marx A. A CTLA4high genotype is associated with myasthenia gravis in thymoma patients. [Comparative Study. Journal Article. Research Support, Non-U.S. Gov't] Annals of Neurology. 58(4):644-8, 2005 Oct.
UI: 16178018

Authors Full Name
Chuang, Wen-Yu. Strobel, Philipp. Gold, Ralf. Nix, Wilfred. Schalke, Berthold. Kiefer, Reinhard. Opitz, Andreas. Klinker, Erdwine. Muller-Hermelink, Hans K. Marx, Alexander.

Zisis C. Rontogianni D. Tzavara C. Stefanaki K. Chatzimichalis A. Loutsidis A. Iliadis K. Kontaxis A. Dosios T. Bellenis I. Prognostic factors in thymic epithelial tumors undergoing complete resection. [Journal Article] Annals of Thoracic Surgery. 80(3):1056-62, 2005 Sep.
UI: 16122486

Authors Full Name
Zisis, Charalambos. Rontogianni, Dimitra. Tzavara, Chara. Stefanaki, Kalliopi. Chatzimichalis, Antonios. Loutsidis, Antonios. Iliadis, Kosmas. Kontaxis, Argirios. Dosios, Theodosios. Bellenis, Ion.

Huang CS. Hsu HS. Huang BS. Lee HC. Kao KP. Hsu WH. Huang MH. Factors influencing the outcome of transsternal thymectomy for myasthenia gravis. [Journal Article] Acta Neurologica Scandinavica. 112(2):108-14, 2005 Aug.
UI: 16008537

Authors Full Name
Huang, C-S. Hsu, H-S. Huang, B-S. Lee, H-C. Kao, K-P. Hsu, W-H. Huang, M-H.

Suzuki S. Shimoda M. Kawamura M. Sato H. Nogawa S. Tanaka K. Suzuki N. Kuwana M. Myasthenia gravis accompanied by alopecia areata: clinical and immunogenetic aspects. [Journal Article. Research Support, Non-U.S. Gov't] European Journal of Neurology. 12(7):566-70, 2005 Jul.
UI: 15958099

Authors Full Name
Suzuki, S. Shimoda, M. Kawamura, M. Sato, H. Nogawa, S. Tanaka, K. Suzuki, N. Kuwana, M.

Nagayasu T. Yamayoshi T. Matsumoto K. Ide N. Hashizume S. Nomura M. Muraoka M. Tagawa T. Akamine S. Oka T. Beneficial effects of plasmapheresis before thymectomy on the outcome in myasthenia gravis. [Comparative Study. Evaluation Studies. Journal Article] Japanese Journal of Thoracic & Cardiovascular Surgery. 53(1):2-7, 2005 Jan.
UI: 15724495

Authors Full Name
Nagayasu, Takeshi. Yamayoshi, Takatomo. Matsumoto, Keitaro. Ide, Noboru. Hashizume, Satoshi. Nomura, Masahito. Muraoka, Masashi. Tagawa, Tsutomu. Akamine, Shinji. Oka, Tadayuki.

Panda S. Goyal V. Behari M. Singh S. Srivastava T. Myasthenic crisis: a retrospective study.[see comment]. [Journal Article] Neurology India. 52(4):453-6, 2004 Dec.
UI: 15626832

Authors Full Name
Panda, S. Goyal, Vinay. Behari, M. Singh, S. Srivastava, T.

Kawaguchi N. Kuwabara S. Nemoto Y. Fukutake T. Satomura Y. Arimura K. Osame M. Hattori T. The Study Group for Myasthenia Gravis in Japan. Treatment and outcome of myasthenia gravis: retrospective multi-center analysis of 470 Japanese patients, 1999-2000. [Comparative Study. Journal Article. Multicenter Study. Research Support, Non-U.S. Gov't] Journal of the Neurological Sciences. 224(1-2):43-7, 2004 Sep 15.
UI: 15450770

Authors Full Name
Kawaguchi, Naoki. Kuwabara, Satoshi. Nemoto, Yuko. Fukutake, Toshio. Satomura, Yoichi. Arimura, Kimiyoshi. Osame, Mitsuhiro. Hattori, Takamichi. The Study Group for Myasthenia Gravis in Japan.

Donmez B. Ozakbas S. Oktem MA. Gedizlioglu M. Coker I. Genc A. Idiman E. HLA genotypes in Turkish patients with myasthenia gravis: comparison with multiple sclerosis patients on the basis of clinical subtypes and demographic features. [Comparative Study. Journal Article] Human Immunology. 65(7):752-7, 2004 Jul.
UI: 15301866

Wirtz PW. van Dijk JG. van Doorn PA. van Engelen BG. van der Kooi AJ. Kuks JB. Twijnstra A. de Visser M. Visser LH. Wokke JH. Wintzen AR. Verschuuren JJ. The epidemiology of the Lambert-Eaton myasthenic syndrome in the Netherlands. [Journal Article. Multicenter Study. Research Support, Non-U.S. Gov't] Neurology. 63(2):397-8, 2004 Jul 27.
UI: 15277653

Huang CM. Yang YH. Chiang BL. Different familial association patterns of autoimmune diseases between juvenile-onset systemic lupus erythematosus and juvenile rheumatoid arthritis. [Journal Article] Journal of Microbiology, Immunology & Infection. 37(2):88-94, 2004 Apr.
UI: 15181489

Levin N. Karussis D. Abramsky O. Parkinson's disease associated with myasthenia gravis. A report of 4 cases. [Case Reports. Letter. Research Support, Non-U.S. Gov't] Journal of Neurology. 250(6):766-7, 2003 Jun.
UI: 12862037

Authors Full

Varelas PN. Chua HC. Natterman J. Barmadia L. Zimmerman P. Yahia A. Ulatowski J. Bhardwaj A. Williams MA. Hanley DF. Ventilatory care in myasthenia gravis crisis: assessing the baseline adverse event rate.[see comment]. [Journal Article] Critical Care Medicine. 30(12):2663-8, 2002 Dec.
UI: 12483056

Cordero Escobar I. Benitez Tang SM. Parisi Lopez N. [Use of mivacurium chloride during transsternal thymectomy in myasthenic patient]. [Spanish] [English Abstract. Journal Article] Revista Espanola de Anestesiologia y Reanimacion. 49(7):360-4, 2002 Aug-Sep.
UI: 12455116

Rao KS. Maikhuri RK. Nautiyal S. Saxena KG. Crop damage and livestock depredation by wildlife: a case study from Nanda Devi Biosphere Reserve, India. [Journal Article. Research Support, Non-U.S. Gov't] Journal of Environmental Management. 66(3):317-27, 2002 Nov.
UI: 12448409

Shimizu M. Ohuchi M. Ohmi M. Nakame T. [Management of myasthenia gravis in association with thymoma]. [Japanese] [English Abstract. Journal Article] Kyobu Geka - Japanese Journal of Thoracic Surgery. 55(11):981-5, 2002 Oct.
UI: 12391697

Kollar S. Dioszeghy P. Hallay J. Kovacs G. [Surgical aspects in the treatment of myasthenia gravis]. [Hungarian] [English Abstract. Journal Article] Magyar Sebeszet. 55(4):233-6, 2002 Aug.
UI: 12236078

Reingold SC. Prevalence estimates for MS in the United States and evidence of an increasing trend for women.[comment]. [Comment. Letter] Neurology. 59(2):294; author reply 294-5, 2002 Jul 23.
UI: 12136080

Authors Full Name
Reingold, Stephen C.

Ratanakorn D. Vejjajiva A. Long-term follow-up of myasthenia gravis patients with hyperthyroidism. [Journal Article. Research Support, Non-U.S. Gov't] Acta Neurologica Scandinavica. 106(2):93-8, 2002 Aug.
UI: 12100368

Fadool DA. Wachowiak M. Brann JH. Patch-clamp analysis of voltage-activated and chemically activated currents in the vomeronasal organ of Sternotherus odoratus (stinkpot/musk turtle). [Journal Article. Research Support, Non-U.S. Gov't. Research Support, U.S. Gov't, P.H.S.] Journal of Experimental Biology. 204(Pt 24):4199-212, 2001 Dec

Ohno T. Kitoh J. Tanaka S. Nishimura M. Namikawa T. Diabetic cataract of the musk shrew (Suncus murinus, Insectivora) exhibiting spontaneous non-insulin dependent diabetes mellitus (NIDDM). [Journal Article] Experimental Animals. 50(5):431-3, 2001 Oct.
UI: 11769547

Kas J. Kiss D. Simon V. Svastics E. Major L. Szobor A. Decade-long experience with surgical therapy of myasthenia gravis: early complications of 324 transsternal thymectomies.[see comment]. [Journal Article] Annals of Thoracic Surgery. 72(5):1691-7, 2001 Nov.
UI: 11722066

Franciotta D. Cuccia M. Dondi E. Piccolo G. Cosi V. Polymorphic markers in MHC class II/III region: a study on Italian patients with myasthenia gravis. [Journal Article. Research Support, Non-U.S. Gov't] Journal of the Neurological Sciences. 190(1-2):11-6, 2001 Sep 15.
UI: 11574100

Authors Full Name
Franciotta, D. Cuccia, M. Dondi, E. Piccolo, G. Cosi, V.

Roberts PF. Venuta F. Rendina E. De Giacomo T. Coloni GF. Follette DM. Richman DP. Benfield JR. Thymectomy in the treatment of ocular myasthenia gravis.[see comment]. [Journal Article] Journal of Thoracic & Cardiovascular Surgery. 122(3):562-8, 2001 Sep.
UI: 11547310

Yeh JH. Chen WH. Chiu HC. Double filtration plasmapheresis in the treatment of myasthenic crisis--analysis of prognostic factors and efficacy. [Journal Article. Research Support, Non-U.S. Gov't] Acta Neurologica Scandinavica. 104(2):78-82, 2001 Aug.
UI: 11493222

Berke O. von Keyserlingk M. [Increase in the prevalence of Echinococcus multilocularis infection in red foxes in Lower Saxony.]. [German] [English Abstract. Journal Article] DTW - Deutsche Tierarztliche Wochenschrift. 108(5):201-5, 2001 May.
UI: 11417378

Murphy FA. Tucker K. Fadool DA. Sexual dimorphism and developmental expression of signal-transduction machinery in the vomeronasal organ. [Comparative Study. Journal Article. Research Support, Non-U.S. Gov't. Research Support, U.S. Gov't, P.H.S.] Journal of Comparative Neurology. 432(1):61-74, 2001 Mar 26.

Poulas K. Tzartos SJ. The gender gap in autoimmune disease.[comment]. [Comment. Letter] Lancet. 357(9251):234, 2001 Jan 20.
UI: 11213129

Meiering CD. Linial ML. Historical perspective of foamy virus epidemiology and infection. [Review] [130 refs] [Journal Article. Review] Clinical Microbiology Reviews. 14(1):165-76, 2001 Jan.
UI: 11148008

Veney SL. Rissman EF. Freeman LM. Perinatal organization of a sexually dimorphic aromatase enzyme-containing immunoreactive nucleus. [Journal Article. Research Support, U.S. Gov't, P.H.S.] Neuroreport. 11(15):3409-12, 2000 Oct 20.

Goto K. Ohashi H. Takakura A. Itoh T. Current status of Helicobacter contamination of laboratory mice, rats, gerbils, and house musk shrews in Japan. [Journal Article. Research Support, Non-U.S. Gov't] Current Microbiology. 41(3):161-6, 2000 Sep.
UI: 10915200

Ebukuro S. Wakana S. Hioki K. Nomura T. Selective breeding of house musk shrew (Suncus murinus) lines in relation to emesis induced by veratrine sulfate. [Comparative Study. Journal Article. Research Support, Non-U.S. Gov't] Comparative Medicine. 50(3):281-3, 2000 Jun.
UI: 10894492

Shelton GD. Ho M. Kass PH. Risk factors for acquired myasthenia gravis in cats: 105 cases (1986-1998). [Comparative Study. Journal Article] Journal of the American Veterinary Medical Association. 216(1):55-7, 2000 Jan 1.
UI: 10638319

Saoudi A. Bernard I. Hoedemaekers A. Cautain B. Martinez K. Druet P. De Baets M. Guery JC. Experimental autoimmune myasthenia gravis may occur in the context of a polarized Th1- or Th2-type immune response in rats. [Comparative Study. Journal Article. Research Support, Non-U.S. Gov't] Journal of Immunology. 162(12):7189-97, 1999 Jun 15.

Yim AP. Kay RL. Izzat MB. Ng SK. Video-assisted thoracoscopic thymectomy for myasthenia gravis. [Journal Article] Seminars in Thoracic & Cardiovascular Surgery. 11(1):65-73, 1999 Jan.
UI: 9930715

Authors Full Name
Yim, A P. Kay, R L. Izzat, M B. Ng, S K.

Lima-Landman MT. Lapa AJ. Gender does not influence neuromuscular properties in dimorphic skeletal muscles of the toad. [Journal Article] Comparative Biochemistry & Physiology. Part A, Molecular & Integrative Physiology. 121(2):119-26, 1998 Oct.
UI: 9883574

Authors Full Name
Lima-Landman, M T. Lapa, A J.

Freeman LM. Arora T. Rissman EF. Neonatal androgen affects copulatory behavior in the female musk shrew. [Journal Article. Research Support, U.S. Gov't, P.H.S.] Hormones & Behavior. 34(3):231-8, 1998 Dec.
UI: 9878272

Rissman EF. Li X. Sex differences in mammalian and chicken-II gonadotropin-releasing hormone immunoreactivity in musk shrew brain. [Journal Article. Research Support, U.S. Gov't, P.H.S.] General & Comparative Endocrinology. 112(3):346-55, 1998 Dec.
UI: 9843640

Gill CJ. Wersinger SR. Veney SL. Rissman EF. Induction of fos-like immunoreactivity in musk shrews after mating. [Journal Article. Research Support, U.S. Gov't, P.H.S.] Brain Research. 811(1-2):21-8, 1998 Nov 16.
UI: 9804874

Casanova-Estruch B. [Analysis of long-term results of thymectomy in control of myasthenia gravis]. [Spanish] [Comparative Study. English Abstract. Journal Article] Revista de Neurologia. 26(152):567-72, 1998 Apr.
UI: 9796007

Matsuzaki Y. Tomita M. Onitsuka T. Shibata K. Influence of age on extended thymectomy as a treatment for myasthenia gravis. [Comparative Study. Journal Article] Annals of Thoracic & Cardiovascular Surgery. 4(4):192-5, 1998 Aug.
UI: 9738119

Veney SL. Rissman EF. Co-localization of estrogen receptor and aromatase enzyme immunoreactivities in adult musk shrew brain. [Journal Article. Research Support, U.S. Gov't, Non-P.H.S.. Research Support, U.S. Gov't, P.H.S.] Hormones & Behavior. 33(3):151-62, 1998 Jun.
UI: 9698499

Grohar-Murray ME. Becker A. Reilly S. Ricci M. Self-care actions to manage fatigue among myasthenia gravis patients. [Journal Article] Journal of Neuroscience Nursing. 30(3):191-9, 1998 Jun.
UI: 9689611

Authors Full Name
Grohar-Murray, M E. Becker, A. Reilly, S. Ricci, M.

Baggi F. Antozzi C. Andreetta F. Confalonieri P. Ciusani E. Begovich AB. Erlich HA. Cornelio F. Mantegazza R. Identification of a novel HLA class II association with DQB1*0502 in an Italian myasthenic population. [Journal Article] Annals of the New York Academy of Sciences. 841:355-9, 1998 May 13.
UI: 9668258

Knieling J. Weiss H. Faller H. Lang H. Schalke B. Toyka K. [Follow-up of myasthenia gravis. Results of a longitudinal study of the significance of psychosocial predictors]. [German] [English Abstract. Journal Article] Nervenarzt. 69(2):137-44, 1998 Feb.
UI: 9551458

Lucchinetti CF. Kimmel DW. Lennon VA. Paraneoplastic and oncologic profiles of patients seropositive for type 1 antineuronal nuclear autoantibodies. [Journal Article. Research Support, U.S. Gov't, P.H.S.] Neurology. 50(3):652-7, 1998 Mar.
UI: 9521251

Jongen JL. van Doorn PA. van der Meche FG. High-dose intravenous immunoglobulin therapy for myasthenia gravis. [Journal Article] Journal of Neurology. 245(1):26-31, 1998 Jan.
UI: 9457625

Authors Full Name
Jongen, J L. van Doorn, P A. van der Meche, F G.

Sokolov VE. Prikhod'ko VI. [The taxonomy of the musk deer (Artiodactyla, Mammalia)]. [Russian] [English Abstract. Journal Article. Research Support, Non-U.S. Gov't] Izvestiia Akademii Nauk. Seriia Biologicheskaia / Rossiiskaia Akademiia Nauk. (6):677-87, 1997 Nov-Dec.
UI: 9518057

Shelton GD. Schule A. Kass PH. Risk factors for acquired myasthenia gravis in dogs: 1,154 cases (1991-1995). [Journal Article] Journal of the American Veterinary Medical Association. 211(11):1428-31, 1997 Dec 1.
UI: 9394894

Gaynor AR. Shofer FS. Washabau RJ. Risk factors for acquired megaesophagus in dogs. [Comparative Study. Journal Article] Journal of the American Veterinary Medical Association. 211(11):1406-12, 1997 Dec 1.
UI: 9394890

Sayers BM. Fliedner TM. The critique of DALYs: a counter-reply. [Journal Article] Bulletin of the World Health Organization. 75(4):383-4, 1997.
UI: 9342898

Authors Full Name
Sayers, B M. Fliedner, T M

Murray CJ. Lopez AD. The utility of DALYs for public health policy and research: a reply. [Comparative Study. Journal Article] Bulletin of the World Health Organization. 75(4):377-81, 1997.
UI: 9342897

Mason WP. Graus F. Lang B. Honnorat J. Delattre JY. Valldeoriola F. Antoine JC. Rosenblum MK. Rosenfeld MR. Newsom-Davis J. Posner JB. Dalmau J. Small-cell lung cancer, paraneoplastic cerebellar degeneration and the Lambert-Eaton myasthenic syndrome. [Comparative Study. Journal Article. Research Support, Non-U.S. Gov't. Research Support, U.S. Gov't, P.H.S.] Brain. 120 ( Pt 8):1279-300, 1997 Aug.
UI: 9278623

Authors Full Name

Berrouschot J. Baumann I. Kalischewski P. Sterker M. Schneider D. Therapy of myasthenic crisis.[see comment]. [Review] [51 refs] [Journal Article. Review] Critical Care Medicine. 25(7):1228-35, 1997 Jul.
UI: 9233752

Authors Full Name

Sommer N. Sigg B. Melms A. Weller M. Schepelmann K. Herzau V. Dichgans J. Ocular myasthenia gravis: response to long-term immunosuppressive

Beekman R. Kuks JB. Oosterhuis HJ. Myasthenia gravis: diagnosis and follow-up of 100 consecutive patients. [Journal Article] Journal of Neurology. 244(2):112-8, 1997 Feb.
UI: 9120493

Khoo SW. Tay YK. Tham SN. Photodermatoses in a Singapore skin referral centre. [Journal Article] Clinical & Experimental Dermatology. 21(4):263-8, 1996 Jul.
UI: 8959895

Lee WS. Heo DS. Bang YJ. Lee KS. Ahn JS. Jung CW. Han SK. Sung SW. Kim JH. Shim YS. Park CI. Kim NK. Prognostic factors of patients with thymoma. [Journal Article. Research Support, Non-U.S. Gov't] Korean Journal of Internal Medicine. 11(1):40-9, 1996 Jan.
UI: 8882475

Rissman EF. Harada N. Roselli CE. Effect of vorozole, an aromatase enzyme inhibitor, on sexual behavior, aromatase activity and neural immunoreactivity. [Journal Article. Research Support, U.S. Gov't, Non-P.H.S.] Journal of Neuroendocrinology. 8(3):199-210, 1996 Mar.
UI: 8730654

Authors Full Name
Rissman, E F. Harada, N. Roselli, C E.

Hjelmstrom P. Giscombe R. Lefvert AK. Pirskanen R. Kockum I. Landin-Olsson M. Sanjeevi CB. Polymorphic amino acid domains of the HLA-DQ molecule are associated with disease heterogeneity in myasthenia gravis. [Journal Article. Research Support, Non-U.S. Gov't] Journal of Neuroimmunology. 65(2):125-31, 1996 Apr.

Hjelmstrom P. Giscombe R. Lefvert AK. Pirskanen R. Kockum I. Landin-Olsson M. Sanjeevi CB. Different HLA-DQ are positively and negatively associated in Swedish patients with myasthenia gravis. [Journal Article. Research Support, Non-U.S. Gov't] Autoimmunity. 22(1):59-65, 1995.
UI: 8882423

Yamagata T. Ohishi K. Faruque MO. Masangkay JS. Ba-Loc C. Vu-Binh D. Mansjoer SS. Ikeda H. Namikawa T. Genetic variation and geographic distribution on the mitochondrial DNA in local populations of the musk shrew, Suncus murinus. [Journal Article. Research Support, Non-U.S. Gov't] Japanese Journal of Genetics. 70(3):321-37, 1995 Jun.

Ishikawa A. Yamagata T. Namikawa T. Relationships between morphometric and mitochondrial DNA differentiation in laboratory strains of musk shrews (Suncus murinus). [Journal Article] Japanese Journal of Genetics. 70(1):57-74, 1995 Feb.

Barnes PR. Kanabar DJ. Brueton L. Newsom-Davis J. Huson SM. Mann NP. Hilton-Jones D. Recurrent congenital arthrogryposis leading to a diagnosis of myasthenia gravis in an initially asymptomatic mother. [Case Reports. Journal Article] Neuromuscular Disorders. 5(1):59-65, 1995 Jan.
UI: 7719143

Horiki T. Inoko H. Moriuchi J. Ichikawa Y. Arimori S. Combinations of HLA-DPB1 and HLA-DQB1 alleles determine susceptibility to early-onset myasthenia gravis in Japan. [Comparative Study. Journal Article. Research Support, Non-U.S. Gov't] Autoimmunity. 19(1):49-54, 1994.
UI: 7749041

Andonopoulos AP. Terzis E. Tsibri E. Papasteriades CA. Papapetropoulos T. D-penicillamine induced myasthenia gravis in rheumatoid arthritis: an unpredictable common occurrence?. [Journal Article] Clinical Rheumatology. 13(4):586-8, 1994 Dec.
UI: 7697959

Sanders DB. Lambert-Eaton myasthenic syndrome: pathogenesis and treatment. [Review] [26 refs] [Journal Article. Review] Seminars in Neurology. 14(2):111-7, 1994 Jun.
UI: 7984825

Martinez-Castrillo JC. Orensanz LM. Jimenez-Escrig A. Somoza E. Cut off selection in anti-acetylcholine receptor antibody determination.[comment]. [Comment. Letter] Journal of Neurology, Neurosurgery & Psychiatry. 57(8):1018, 1994 Aug.

Ruiz-Neto PP. Halpern H. Cremonesi E. Rapid inhalation induction with halothane-nitrous oxide for myasthenic patients. [Comparative Study. Journal Article. Research Support, Non-U.S. Gov't] Canadian Journal of Anaesthesia. 41(2):102-6, 1994 Feb

Godeluck B. Duplantier JM. Ba K. Trape JF. A longitudinal survey of Borrelia crocidurae prevalence in rodents and insectivores in Senegal. [Journal Article] American Journal of Tropical Medicine & Hygiene. 50(2):165-8, 1994 Feb.

Balo-Banga JM. Pinter E. Nemeth G. [Incidence of SLE in a patient thymectomized for myasthenia gravis]. [Hungarian] [Case Reports. English Abstract. Journal Article] Orvosi Hetilap. 134(25):1369-72, 1993 Jun 20.
UI: 8332357

Paradis CM. Friedman S. Lazar RM. Kula RW. Anxiety disorders in a neuromuscular clinic. [Journal Article. Research Support, U.S. Gov't, P.H.S.] American Journal of Psychiatry. 150(7):1102-4, 1993 Jul.
UI: 8317584

Baraka A. Anesthesia and myasthenia gravis.[see comment]. [Review] [61 refs] [Journal Article. Review] Middle East Journal of Anesthesiology. 12(1):9-35, 1993 Feb.
UI: 8316155

Graus YM. De Baets MH. Myasthenia gravis: an autoimmune response against the acetylcholine receptor. [Review] [277 refs] [Journal Article. Review] Immunologic Research. 12(1):78-100, 1993.
UI: 7685805

Authors Full Name
Graus, Y M. De Baets, M H.

Patrick C. Organ-specific autoimmune diseases. [Review] [15 refs] [Journal Article. Review] Immunology Series. 58:423-36, 1993.
UI: 8424986

Authors Full Name
Patrick, C.

Bhettay EM. Kalla AA. Coexistence of myasthenia gravis and seropositive juvenile chronic arthritis. [Case Reports. Journal Article] Clinical & Experimental Rheumatology. 10(5):499-501, 1992 Sep-Oct.
UI: 1458704

Bhettay EM. Kalla AA. Coexistence of myasthenia gravis and seropositive juvenile chronic arthritis. [Case Reports. Journal Article] Clinical & Experimental Rheumatology. 10(5):499-501, 1992 Sep-Oct.
UI: 1458704

Wang LS. Huang MH. Lin TS. Huang BS. Chien KY. Malignant thymoma. [Journal Article] Cancer. 70(2):443-50, 1992 Jul 15.
UI: 1617594

Van Kempen GT. Molenaar PC. Effect of estradiol and progesterone on muscle weight and acetylcholine receptors in "myasthenic" rats. [Journal Article. Research Support, Non-U.S. Gov't] Journal of Neural Transmission - General Section. 87(3):193-7, 1992.
UI: 1581018

Tzartos SJ. Cung MT. Demange P. Loutrari H. Mamalaki A. Marraud M. Papadouli I. Sakarellos C. Tsikaris V. The main immunogenic region (MIR) of the nicotinic acetylcholine receptor and the anti-MIR antibodies. [Review] [157 refs] [Journal Article. Research Support, Non-U.S. Gov't. Review] Molecular Neurobiology. 5(1):1-29, 1991.

Beghi E. Antozzi C. Batocchi AP. Cornelio F. Cosi V. Evoli A. Lombardi M. Mantegazza R. Monticelli ML. Piccolo G. et al. Prognosis of myasthenia gravis: a multicenter follow-up study of 844 patients. [Clinical Trial. Comparative Study. Journal Article. Multicenter Study] Journal of the Neurological Sciences. 106(2):213-20, 1991 Dec.

Gomm SA. Thatcher N. Barber PV. Cumming WJ. A clinicopathological study of the paraneoplastic neuromuscular syndromes associated with lung cancer. [Journal Article. Research Support, Non-U.S. Gov't] Quarterly Journal of Medicine. 75(278):577-95, 1990 Jun.
UI: 2171009

Murray KB. Myasthenia gravis. [Case Reports. Journal Article] Kansas Medicine. 91(6):181-3, 1990 Jun.
UI: 2398638

Authors Full Name
Murray, K B.

Clongsusuek P. Muntarbhorn K. Thanaviratananich S. Myasthenia gravis poses a diagnostic problem for the otolaryngologist: a closer look at symptoms may alert the possibility of myasthenia gravis. [Journal Article] Auris, Nasus, Larynx. 17(1):55-60, 1990.
UI: 2390034

Cosi V. Citterio A. Pasquino C. A study of hand preference in myasthenia gravis. [Journal Article] Cortex. 24(4):573-7, 1988 Dec.
UI: 3219871

O'Brien PC. A nonparametric test for association with censored data. [Journal Article] Biometrics. 34(2):243-50, 1978 Jun.
UI: 667272

Castleman B. The pathology of the thymus gland in myasthenia gravis. [Journal Article] Annals of the New York Academy of Sciences. 135(1):496-505, 1966 Jan 26.
UI: 5221360

Authors Full Name

Meyer E. Psychological disturbances in myasthenia gravis: a predictive study. [Journal Article] Annals of the New York Academy of Sciences. 135(1):417-23, 1966 Jan 26.
UI: 5221353

Authors Full Name
Meyer, E.

Glaser GH. Crisis, precrisis and drug resistance in myasthenia gravis. [Journal Article] Annals of the New York Academy of Sciences. 135(1):335-49, 1966 Jan 26.
UI: 5221348

Authors Full Name
Glaser, G H.

JENKINS LC. CHANG J. SAXTON GD. MYASTHENIA GRAVIS: ANESTHETIC AND SURGICAL MANAGEMENT OF THE PATIENT UNDERGOING THYMECTOMY. [Journal Article] Canadian Medical Association Journal. 93:198-203, 1965 Jul 31.
UI: 14323663

SCHWAB RS. WILKINS EW Jr. HEAD JM. PONTOPPIDAN H. VIETS HR. THYMECTOMY IN MYASTHENIA GRAVIS. [Journal Article] JAMA. 187:850-1, 1964 Mar 14.
UI: 14094327.

# EMBASE

Kanazawa M., Shimohata T., Tanaka K., Nishizawa M. Clinical features of patients with myasthenia gravis associated with autoimmune diseases. [Journal: Article] European Journal of Neurology. 14(12)(pp 1403-1404), 2007. Date of Publication: Dec 2007.

Steck A.J. Use of intravenous immunoglobulin in neurological disorders. [Journal: Review] Revue Medicale Suisse. 1(17)(pp 1167-1170), 2005. Date of Publication: 27 Apr 2005.

Murthy J.M.K., Meena A.K., Chowdary G.V.S., Naryanan J.T. Myasthenic crisis: Clinical features, complications and mortality. [Journal: Article] Neurology India. 53(1)(pp 37-40), 2005. Date of Publication: Jan 2005

Aarli J.A., Romi F., Skeie G.O., Gilhus N.E. Myasthenia gravis in individuals over 40. [Journal: Conference Paper] Annals of the New York Academy of Sciences. 998(pp 424-431), 2003. Date of Publication: 2003.

Xu J., Yang M., Li B. Myasthenia gravis: Clinical study in 2 385 patients. [Journal: Article] Chinese Journal of Neurology. 32(6)(pp 347-350), 1999. Date of Publication: 1999.

Bartley G.B. The epidemiologic characteristics and clinical course of ophthalmopathy associated with autoimmune thyroid disease in Olmstead County, Minnesota. [Journal: Conference Paper] Transactions of the American Ophthalmological Society. 92(pp 477-588), 1994. Date of Publication: 1994.

Konishi T., Ohta K., Shigemoto K., Ohta M. Anti-alkaline phosphatase antibody positive myasthenia gravis. [Journal: Article] Journal of the Neurological Sciences. 263(1-2)(pp 89-93), 2007. Date of Publication: 15 Dec 2007.

Zeher M., Szegedi G. Types of autoimmune disorders. Classification. [Journal: Article] Orvosi Hetilap. 148(SUPPL. 1)(pp 21-24), 2007. Date of Publication: 08 Apr 2007.

Lipsky P.E., Radbruch A. Current Topics in Microbiology and Immunology: Preface. [Book Series: Editorial] Current Concepts in Autoimmunity and Chronic InflammationCurrent Topics in Microbiology and Immunology. 305(pp V-VI), 2006. Date of Publication: 2006.

Goldacre M.J., Wotton C.J., Seagroatt V., Yeates D. Immune-related disease before and after vasectomy: An epidemiological database study. [Journal: Article] Human Reproduction. 22(5)(pp 1273-1278), 2007. Date of Publication: May 2007.

Wolfe G.I., Trivedi J.R., Oh S.J. Clinical review of muscle-specific tyrosine kinase-antibody positive myasthenia gravis. [Journal: Review] Journal of Clinical Neuromuscular Disease. 8(4)(pp 217-224), 2007. Date of Publication: Jun 2007.

Ludvigsson J.F., Olsson T., Ekbom A., Montgomery S.M. A population-based study of coeliac disease, neurodegenerative and neuroinflammatory diseases. [Journal: Article] Alimentary Pharmacology and Therapeutics. 25(11)(pp 1317-1327), 2007. Date of Publication: Jun 2007.

Arrowsmith J.E. The neuromuscular junction. [Journal: Review] Surgery. 25(3)(pp 105-111), 2007. Date of Publication: Mar 2007.

Zhang X., Yang M., Xu J., Zhang M., Lang B., Wang W., Vincent A. Clinical and serological study of myasthenia gravis in HuBei Province, China. [Journal: Article] Journal of Neurology, Neurosurgery and Psychiatry. 78(4)(pp 386-390), 2007. Date of Publication: Apr 2007.

Barbaud A., Carlander B., Pages M. Late onset forms of myasthenia gravis. Comparison with early-onset myasthenia gravis. [Journal: Conference Paper] Revue Neurologique. 162(10)(pp 990-996), 2006. Date of Publication: Oct 2006.

Toth C., McDonald D., Oger J., Brownell K. Acetylcholine receptor antibodies in myasthenia gravis are associated with greater risk of diabetes and thyroid disease. [Journal: Article] Acta Neurologica Scandinavica. 114(2)(pp 124-132), 2006. Date of Publication: Aug 2006.

Darnell R.B., Posner J.B. Paraneoplastic Syndromes Affecting the Nervous System. [Journal: Article] Seminars in Oncology. 33(3)(pp 270-298), 2006. Date of Publication: Jun 2006.

Konishi T. Recent topics in myasthenia gravis. [Journal: Article] Japan Medical Association Journal. 49(3)(pp 135-137), 2006. Date of Publication: Mar 2006.

Newsom-Davis J. Neuromuscular junction channelopathies: A brief overview. [Journal: Review] Acta Neurologica Belgica. 105(4)(pp 181-186), 2005. Date of Publication: Dec 2005.

Schmidt S. N., Salinas C. M.E., Erazo T. R. Myastenia gravis in paediatrics. [Journal: Review] Revista Chilena de Pediatria. 76(3)(pp 291-298), 2005. Date of Publication: Jun 2005.

Motomura M., Shiraishi H., Yoshimura T., Tsujihata M. Myasthenia gravis/Lambert-Eaton myasthenic syndrome. [Journal: Review] Neuro-Ophthalmology Japan. 22(3)(pp 313-325), 2005. Date of Publication: 2005.

.

Lavrnic D., Losen M., Vujic A., De Baets M., Hajdukovic L.J., Stojanovic V., Trikic R., Djukic P., Apostolski S. The features of myasthenia gravis with autoantibodies to MuSK. [Journal: Article] Journal of Neurology, Neurosurgery and Psychiatry. 76(8)(pp 1099-1102), 2005. Date of Publication: Aug 2005

Fairweather D., Rose N.R. Women and autoimmune diseases. [Journal: Conference Paper] Emerging Infectious Diseases. 10(11)(pp 2005-2011), 2004. Date of Publication: Nov 2004.

Kawaguchi N., Kuwabara S., Nemoto Y., Fukutake T., Satomura Y., Arimura K., Osame M., Hattori T. Treatment and outcome of myasthenia gravis: Retrospective multi-center analysis of 470 Japanese patients, 1999-2000. [Journal: Article] Journal of the Neurological Sciences. 224(1-2)(pp 43-47), 2004. Date of Publication: 15 Sep 2004.

Kalaydjieva L., Gresham D., Calafell F. Genetic studies of the Roma (Gypsies): A review. [Journal: Review] BMC Medical Genetics. 2, 2001. Date of Publication: 02 Apr 2001.

Zafeiriou D.I., Pitt M., De Sousa C. Clinical and neurophysiological characteristics of congenital myasthenic syndromes presenting in early infancy. [Journal: Article] Brain and Development. 26(1)(pp 47-52), 2004. Date of Publication: Jan 2004.

Liu W., Liu G., Fan Z., Gai X. Myasthenia gravis in pediatric and elderly patients. [Journal: Article] Chinese Medical Journal. 116(10)(pp 1578-1581), 2003. Date of Publication: Oct 2003

Vincent A., Bowen J., Newsom-Davis J., McConville J. Seronegative generalised myasthenia gravis: Clinical features, antibodies, and their targets. [Journal: Review] Lancet Neurology. 2(2)(pp 99-106), 2003. Date of Publication: 01 Feb 2003.

Evoli A., Tonali P.A., Padua L., Lo Monaco M., Scuderi F., Batocchi A.P., Marino M., Bartoccioni E. Clinical correlates with anti-MuSK antibodies in generalized seronegative myasthenia gravis. [Journal: Article] Brain. 126(10)(pp 2304-2311), 2003. Date of Publication: Oct 2003.

Haliloglu G., Anlar B., Aysun S., Topcu M., Topaloglu H., Turanli G., Yalnizoglu D. Gender prevalence in childhood multiple sclerosis and myasthenia gravis. [Journal: Article] Journal of Child Neurology. 17(5)(pp 390-392), 2002. Date of Publication: May 2002.

Karcagi V., Tournev I., Schmidt C., Herczegflvi A., Guergueltcheva V., Litvinenko I., Song I.-H., Abicht A., Lochmuller H. Congenital myasthenic syndrome in South-Eastern European Roma (Gypsies). [Journal: Article] Acta Myologica. 20(DEC.)(pp 231-237), 2001. Date of Publication: 2001.

Fink J.N., Wallis W.E., Haydock D.A. Myasthenia gravis with thymoma is more common in the Maori and Pacific Island populations in New Zealand. [Journal: Article] Internal Medicine Journal. 31(4)(pp 206-210), 2001. Date of Publication: 2001.

Vega-Garces W.J. Myasthenia gravis in patients over 50 years old. [Journal: Article] Revista de Neurologia. 28(10)(pp 948-951), 1999. Date of Publication: 31 May

Schon F., Drayson M., Thompson R.A. Myasthenia gravis and elderly people. [Journal: Article] Age and Ageing. 25(1)(pp 56-58), 1996. Date of Publication: 1996.

# . EMBASE

Malik K.J., Lee M.S., Park D.J.J., Harrison A.R. Lash ptosis in congenital and acquired blepharoptosis. [Journal: Article] Archives of Ophthalmology. 125(12)(pp 1613-1615), 2007. Date of Publication: Dec 2007.

High W.A. Nephrogenic systemic fibrosis and gadolinium-based contrast agents. [Journal: Review] Expert Review of Dermatology. 2(5)(pp 593-605), 2007. Date of Publication: Oct 2007

Toyka K.V., Gold R. Treatment of myasthenia gravis. [Journal: Review] Schweizer Archiv fur Neurologie und Psychiatrie. 158(7)(pp 309-321), 2007. Date of Publication: Nov 2007

Reddy A.K., Foroozan R., Arat Y.O., Edmond J.C., Yen M.T. Ptosis in Young Soft Contact Lens Wearers. [Journal: Letter] Ophthalmology. 114(12)(pp 2370-2370.e1), 2007. Date of Publication: Dec 2007

Vrabec J.T., Isaacson B., Van Hook J.W. Bell's palsy and pregnancy. [Journal: Article] Otolaryngology - Head and Neck Surgery. 137(6)(pp 858-861), 2007. Date of Publication: Dec 2007.

Aghajanzadeh M., Khoshrang H., Mohammadzadeh A., Roudbari S.A., Ghayeghran A.R. Thymectomy for myasthenia gravis: Prognostic factors in 70 patients. [Journal: Article] Asian Cardiovascular and Thoracic Annals. 15(5)(pp 371-375), 2007. Date of Publication: Oct 2007.

Damadoglu E., Salturk C., Takir H.B., Ertugrul M., Atasalihi A., Yilmaz A. Mediastinal thymolipoma: An analysis of 10 cases. [Journal: Article] Respirology. 12(6)(pp 924-927), 2007. Date of Publication: Nov 2007.

Roig J., Arnau J.M., Vallano A., Rello J. New developments in therapeutic agents for Legionnaires' disease. [Journal: Article] Anti-Infective Agents in Medicinal Chemistry. 6(4)(pp 228-242), 2007. Date of Publication: Oct 2007.

Briemberg H.R. Neuromuscular diseases in pregnancy. [Journal: Review] Seminars in Neurology. 27(5)(pp 460-466), 2007. Date of Publication: Nov 2007.

Stephenson K., Tschaburn C.M., Vasu S., Rashba E.J. When, how, and why should sinus rhythm be restored in patients with persistent atrial fibrillation?. [Journal: Review] Current Treatment Options in Cardiovascular Medicine. 9(5)(pp 372-378), 2007. Date of Publication: Oct 2007

Lode H.M. Managing community-acquired pneumonia: A European perspective. [Journal: Review] Respiratory Medicine. 101(9)(pp 1864-1873), 2007. Date of Publication: Sep 2007.

Chan K.H., Cheung R.T.F., Mak W., Ho S.L. Nonthymoma early-onset- and late-onset-generalized myasthenia gravis-A retrospective hospital-based study. [Journal: Article] Clinical Neurology and Neurosurgery. 109(8)(pp 686-691), 2007. Date of Publication: Oct 2007.

Alemany-Rodriguez M.J., Aladro Y., Amela-Peris R., Perez-Vieitez M.C., Reyes-Yanez M.P., Deniz-Naranjo M.C., Sanchez-Garcia F. Autoimmune diseases and multiple sclerosis. [Journal: Article] Revista de Neurologia. 40(10)(pp 594-597), 2005. Date of Publication: May 2005.

Szczepiorkowski Z.M., Bandarenko N., Kim H.C., Linenberger M.L., Marques M.B., Sarode R., Schwartz J., Shaz B.H., Weinstein R., Wirk A., Winters J.L. Guidelines on the use of therapeutic apheresis in clinical practice-evidence-based approach from the Apheresis Applications Committee of the American Society for Apheresis. [Journal: Review] Journal of Clinical Apheresis. 22(3)(pp 106-175), 2007. Date of Publication: Jun 2007.

Chirapapaisan N., Tanormrod S., Chuenkongkaew W. Factors associated with insensitivity to pyridostigmine therapy in Thai patiens with ocular myasthenia gravis. [Journal: Article] Asian Pacific Journal of Allergy and Immunology. 25(1)(pp 13-16), 2007. Date of Publication: Mar 2007

de los Angeles Avaria M., Kleinsteuber K., Novoa F., Faundez P., Carvallo P. Myotonic Dystrophy in a Female with Myasthenia Gravis. [Journal: Article] Pediatric Neurology. 36(6)(pp 421-423), 2007. Date of Publication: Jun 2007

Garcia-Carrasco M., Escarcega R.O., Fuentes-Alexandro S., Riebeling C., Cervera R. Therapeutic options in autoimmune myasthenia gravis. [Journal: Review] Autoimmunity Reviews. 6(6)(pp 373-378), 2007. Date of Publication: Jun 2007.

Botta S., Roveto S., Rimoldi D. Seric 21-hydroxilase antibodies in patients with anti-microsomal fraction antibodies. Autoimmune polyendocrine syndrome. [Journal: Article] Medicina. 67(2)(pp 143-146), 2007. Date of Publication: 2007.

Ramagopalan S.V., Dyment D.A., Valdar W., Herrera B.M., Criscuoli M., Yee I.M.L., Sadovnick A.D., Ebers G.C. Autoimmune disease in families with multiple sclerosis: a population-based study. [Journal: Article] Lancet Neurology. 6(7)(pp 604-610), 2007. Date of Publication: Jul 2007.

Bodemer C. Photosensibility and systemic disease in children. [Journal: Review] Annales de Dermatologie et de Venereologie. 134(5 C2)(pp 4S45-4S49), 2007. Date of Publication: May 2007

Dunand M., Lalive P.H., Vokatch N., Kuntzer T. Myasthenia gravis: Treatments and remissions. [Journal: Review] Revue Medicale Suisse. 3(110)(pp 1185-1190), 2007. Date of Publication: 09 May 2007.

Vingerhoets F., Landis T., Burkhard P.R. Neurology of the peripheral nervous system. [Journal: Editorial] Revue Medicale Suisse. 3(110)(pp 1155-1156), 2007. Date of Publication: 09 May 2007.

Kulaksizoglu I.B. Mood and anxiety disorders in patients with myasthenia gravis: Aetiology, diagnosis and treatment. [Journal: Review] CNS Drugs. 21(6)(pp 473-481), 2007. Date of Publication: 2007.

Gurcan H.M., Ahmed A.R. Efficacy of various intravenous immunoglobulin therapy protocols in autoimmune and chronic inflammatory disorders. [Journal: Article] Annals of Pharmacotherapy. 41(5)(pp 812-823), 2007. Date of Publication: May 2007.

Lin M.W., Kirkpatrick P.E., Riminton D.S. How intravenous immunoglobulin is used in clinical practice: Audits of two Sydney teaching hospitals. [Journal: Article] Internal Medicine Journal. 37(5)(pp 308-314), 2007. Date of Publication: May 2007.

Stewart S.B., Robertson K.R., Johnson K.M., Howard Jr. J.F. The prevalence of depression in myasthenia gravis. [Journal: Article] Journal of Clinical Neuromuscular Disease. 8(3)(pp 111-115), 2007. Date of Publication: Mar 2007.

Aprotinin associated with increased risk of death during the 5 years following CABG surgery. [Journal: Short Survey] Formulary. 42(3)(pp 149), 2007. Date of Publication: Mar 2007.

Engel A.G. The Therapy of Congenital Myasthenic Syndromes. [Journal: Article] Neurotherapeutics. 4(2)(pp 252-257), 2007. Date of Publication: Apr 2007

Zafarullah M., Tayyab A.A., Sahi T.-U.-N., Kanju M.I. Silicone band used as brow suspension in ptosis surgery. [Journal: Article] Medical Forum Monthly. 16(3)(pp 11-14), 2005. Date of Publication: Mar 2005

Hemminki K., Li X., Sundquist K. Familial risks for diseases of myoneural junction and muscle in siblings based on hospitalizations and deaths in Sweden. [Journal: Article] Twin Research and Human Genetics. 9(4)(pp 573-579), 2006. Date of Publication: Aug 2006.

Kraus J., Teismann I., Kellinghaus C., Duning T., Ringelstein E.B., Nabavi D.G., Schabitz W.-R. Temporal coincidence between AMAN type of GBS and myasthenia gravis [8]. [Journal: Letter] Journal of Neurology. 254(2)(pp 264-265), 2007. Date of Publication: Feb 2007.

Liu W.-B., Men L.-N., Chen Z.-G., Luo H.-H., Huang R.-X. Long-term efficacy of enlarged thymectomy in treatment of myasthenia gravis and relevant influencing factors; study of 410 cases. [Journal: Article] National Medical Journal of China. 86(45)(pp 3182-3185), 2006. Date of Publication: 05 Dec 2006

Cakar F., Werner P., Augustin F., Schmid T., Wolf-Magele A., Sieb M., Bodner J. A comparison of outcomes after robotic open extended thymectomy for myasthenia gravis. [Journal: Article] European Journal of Cardio-thoracic Surgery. 31(3)(pp 501-505), 2007. Date of Publication: Mar 2007.

Aghamohammadi A., Pouladi N., Parvaneh N., Yeganeh M., Movahedi M., Gharagolou M., Pourpak Z., Rezaei N., Salavati A., Abdollahzade S., Moin M. Mortality and morbidity in common variable immunodeficiency. [Journal: Article] Journal of Tropical Pediatrics. 53(1)(pp 32-38), 2007. Date of Publication: Feb 2007.

Amarenco P., Nadjar M. Telemedicine for improving emergent management of acute cerebrovascular syndromes. [Journal: Article] International Journal of Stroke. 2(1)(pp 47-50), 2007. Date of Publication: Feb 2007.

Grimaud L., Viallet F., Gayraud D., Lejeune A. Late onset myasthenia revealed by a falling head. [Journal: Article] Revue de Geriatrie. 31(10)(pp 813-816), 2006. Date of Publication: Dec 2006.

Nguyen N.Q., Chapman M.J., Fraser R.J., Bryant L.K., Holloway R.H. Erythromycin is more effective than metoclopramide in the treatment of feed intolerance in critical illness. [Journal: Article] Critical Care Medicine. 35(2)(pp 483-489), 2007. Date of Publication: Feb 2007.

Wan Y., Wei Q., Hu J., Jin X., Zhang Z., Zhen H., Liu J. Levels, tissue distribution, and age-related accumulation of synthetic musk fragrances in Chinese sturgeon (Acipenser sinensis): Comparison to organochlorines. [Journal: Article] Environmental Science and Technology. 41(2)(pp 424-430), 2007. Date of Publication: 15 Jan 2007

Desport J.-C., Couratier P., Devalois B. Nutrition in neuromuscular diseases. [Journal: Review] Cahiers de Nutrition et de Dietetique. 41(6)(pp 364-370), 2006. Date of Publication: Dec 2006

Buxeraud J. Norfloxacin. [Journal: Short Survey] Actualites Pharmaceutiques. (458)(pp 31-33), 2006. Date of Publication: Dec 2006.

Gleicher N., Weiner R., Vietzke M. The impact of abnormal autoimmune function on reproduction: Maternal and fetal consequences. [Journal: Article] Journal of Autoimmunity. 27(3)(pp 161-165), 2006. Date of Publication: Nov 2006

Singh N., Reau N. Extrahepatic manifestations of HCV infection: A brief review and update. [Journal: Review] Current Hepatitis Reports. 5(4)(pp 133-141), 2006. Date of Publication: Oct 2006.

Pichat P., Bergis O.E., Terranova J.-P., Urani A., Duarte C., Santucci V., Gueudet C., Voltz C., Steinberg R., Stemmelin J., Oury-Donat F., Avenet P., Griebel G., Scatton B. SSR180711, a novel selective alpha7 nicotinic receptor partial agonist: (II) efficacy in experimental models predictive of activity against cognitive symptoms of schizophrenia. [Journal: Article] Neuropsychopharmacology. 32(1)(pp 17-34), 2007. Date of Publication: 01 Jan 2007.

Bruns A., Meyer O. Neuropsychiatric manifestations of systemic lupus erythematosus. [Journal: Short Survey] Joint Bone Spine. 73(6)(pp 639-645), 2006. Date of Publication: Dec 2006.

Gong Y., Klingenberg S.L., Gluud C. Systematic review and meta-analysis: D-Penicillamine vs. placebo/no intervention in patients with primary biliary cirrhosis - Cochrane Hepato-Biliary Group. [Journal: Review

Triplitt C. Drug interactions of medications commonly used in diabetes. [Journal: Review] Diabetes Spectrum. 19(4)(pp 202-211), 2006. Date of Publication: Sep 2006.

Hill N.S. Neuromuscular disease in respiratory and critical care medicine. [Journal: Conference Paper] Respiratory Care. 51(9)(pp 1065-1071), 2006. Date of Publication: Sep 2006.

Mehta S. Neuromuscular disease causing acute respiratory failure. [Journal: Conference Paper] Respiratory Care. 51(9)(pp 1016-1021), 2006. Date of Publication: Sep 2006.

Ishii W., Matsuda M., Hanyuda M., Momose M., Nakayama J., Ehara T., Ikeda S.-i. Comparison of the histological and immunohistochemical features of the thymus in young- and elderly-onset myasthenia gravis without thymoma. [Journal: Article] Journal of Clinical Neuroscience. 14(2)(pp 110-115), 2007. Date of Publication: Feb 2007.

Bae J.S., Go S.M., Kim B.J. Clinical predictors of steroid-induced exacerbation in myasthenia gravis. [Journal: Article] Journal of Clinical Neuroscience. 13(10)(pp 1006-1010), 2006. Date of Publication: Dec 2006.

Picard C., Parrot A., Mayaud C., Cadranel J. Immune mediated intra-alveolar haemorrhage in the adult. [Journal: Review] Revue des Maladies Respiratoires. 23(HS1)(pp 3S61-3S73), 2006. Date of Publication: Feb 2006.

Dhand U.K., Dhand R. Sleep disorders in neuromuscular diseases. [Journal: Review] Current Opinion in Pulmonary Medicine. 12(6)(pp 402-408), 2006. Date of Publication: Nov 2006.

Gooding M.P., Newton T.J., Bartsch M.R., Hornbuckle K.C. Toxicity of synthetic musks to early life stages of the freshwater mussel Lampsilis cardium. [Journal: Article] Archives of Environmental Contamination and Toxicology. 51(4)(pp 549-558), 2006. Date of Publication: Nov 2006

Campbell W.W. Statin myopathy: The iceberg or its tip?. [Journal: Editorial] Muscle and Nerve. 34(4)(pp 387-390), 2006. Date of Publication: Oct 2006

Wyndaele J.-J. Solifenacin: A new drug for the treatment of overactive bladder and detrusor overactivity. [Journal: Article] Therapy. 3(4)(pp 485-494), 2006. Date of Publication: Jul 2006.

Gotkine M., Fellig Y., Abramsky O. Occurrence of CNS demyelinating disease in patients with myasthenia gravis. [Journal: Article] Neurology. 67(5)(pp 881-883), 2006. Date of Publication: Sep 2006

Nicolle M.W., Rask S., Koopman W.J., George C.F.P., Adams J., Wiebe S. Sleep apnea in patients with myasthenia gravis. [Journal: Article] Neurology. 67(1)(pp 140-142), 2006.

Engel A.G. Light on limb-girdle myasthenia. [Journal: Note] Brain. 129(8)(pp 1938-1939), 2006. Date of Publication: Aug 2006.

Seeman P., Siskova D. Autosomal recessive ethnical diseases of Czech Roma. [Journal: Review] Casopis Lekaru Ceskych. 145(7)(pp 557-560), 2006. Date of Publication: 2006

Rubin J.W. Invited commentary. [Journal: Article] Annals of Thoracic Surgery. 82(3)(pp 1007-1008), 2006. Date of Publication: Sep 2006.

Tomulescu V., Ion V., Kosa A., Sgarbura O., Popescu I. Thoracoscopic Thymectomy Mid-Term Results. [Journal: Article] Annals of Thoracic Surgery. 82(3)(pp 1003-1007), 2006. Date of Publication: Sep 2006.

Taruffi F., Pantalone D., Santoni S., Paolucci R., Andreoli F. Video-thoracoscopic thymectomy. [Journal: Article] Chirurgia. 19(2)(pp 149-155), 2006. Date of Publication: Apr 2006.

Mok C.C., To C.H., Mak A. Neuropsychiatric damage in southern Chinese patients with systemic lupus erythematosus. [Journal: Article] Medicine. 85(4)(pp 221-228), 2006. Date of Publication: Jul 2006

Zenteno J.F.T. Can we consider thymectomy before pregnancy in female patients with myasthenia gravis?. [Journal: Letter] European Journal of Cardio-thoracic Surgery. 30(2)(pp 411-412), 2006. Date of Publication: Aug 2006.

Menon A., Madhukar M., Sreedhar A. Is myasthenia gravis more benign in the Indian population? [5]. [Journal: Letter] Neurology India. 54(2)(pp 217-218), 2006. Date of Publication: 01 Apr 2006.

Kister I., Gulati S., Boz C., Bergamaschi R., Piccolo G., Oger J., Swerdlow M.L. Neuromyelitis optica in patients with myasthenia gravis who underwent thymectomy. [Journal: Article] Archives of Neurology. 63(6)(pp 851-856), 2006. Date of Publication: 2006.

Santos Lasaosa S. Myasthenia gravis: Descriptive analysis of our series. [Journal: Article] Salud (i) Sciencia. 14(3)(pp 136-138), 2006. Date of Publication: May 2006.

Rofail S., Barras M.A. Are patients on long-term corticosteroids receiving bone loss therapy?. [Journal: Article] Journal of Pharmacy Practice and Research. 36(1)(pp 29-31), 2006. Date of Publication: Mar 2006

Shrivastava C.P., Devgarha S., Ahlawat V. Mediastinal tumors: A clinicopathological analysis. [Journal: Article] Asian Cardiovascular and Thoracic Annals. 14(2)(pp 102-104), 2006. Date of Publication: Jun 2006.

Giometto B., Vincent A. Paraneoplastic neurological syndromes. State of the art. [Journal: Conference Paper] Journal of Neuroimmunology. 174(1-2)(pp 192-204), 2006. Date of Publication: May 2006

Owe J.F., Daltveit A.K., Gilhus N.E. Does myasthenia gravis provide protection against cancer?. [Journal: Conference Paper] Acta Neurologica Scandinavica. 113(SUPPL. 183)(pp 33-36), 2006. Date of Publication: May 2006.

Yeh J.-H., Chen W.-H., Huang K.-M., Chiu H.-C. Prethymectomy plasmapheresis in myasthenia gravis. [Journal: Article] Journal of Clinical Apheresis. 20(4)(pp 217-221), 2005. Date of Publication: Dec 2005.

Bressler B. Another tool in the epidemiologist's toolbox. [Journal: Note] Inflammatory Bowel Diseases. 12(3)(pp 245-246), 2006. Date of Publication: Mar 2006.

Morrison G.A., Lang C., Huda S. Botulism in a pregnant intravenous drug abuser. [Journal: Review] Anaesthesia. 61(1)(pp 57-60), 2006. Date of Publication: Jan 2006.

Jitpimolmard S., Taimkao S., Chotmongkol V., Sawanyawisuth K., Vincent A., Newsom-Davis J. Acetylcholine receptor antibody in Thai generalized myasthenia gravis patients. [Journal: Article] Journal of the Medical Association of Thailand. 89(1)(pp 68-71), 2006. Date of Publication: Jan 2006.

Sthoeger Z., Neiman A., Elbirt D., Zinger H., Magen E., Burstein R., Eitan S., Abarbanel J., Mozes E. High prevalence of systemic lupus erythematosus in 78 myasthenia gravis patients: A clinical and serologic study. [Journal: Article] American Journal of the Medical Sciences. 331(1)(pp 4-9), 2006. Date of Publication: Jan 2006.

Etienne M., Weimer L.H. Immune-mediated autonomic neuropathies. [Journal: Review] Current Neurology and Neuroscience Reports. 6(1)(pp 57-64), 2006. Date of Publication: Jan 2006.

Uetrecht J. Role of animal models in the study of drug-induced hypersensitivity reactions. [Journal: Review] AAPS Journal. 7(4), 2005. Article Number: 89. Date of Publication: 13 Jan 2006.

Sarzi-Puttini P., Atzeni F., Capsoni F., Lubrano E., Doria A. Drug-induced lupus erythematosus. [Journal: Review] Autoimmunity. 38(7)(pp 507-518), 2005. Date of Publication: Nov 2005.

Lacomis D. Myasthenic crisis. [Journal: Review] Neurocritical Care. 3(3)(pp 189-194), 2005. Date of Publication: Dec 2005.

Kattach H., Anastasiadis K., Cleuziou J., Buckley C., Shine B., Pillai R., Ratnatunga C. Transsternal thymectomy for myasthenia gravis: Surgical outcome. [Journal: Note] Annals of Thoracic Surgery. 81(1)(pp 305-308), 2006. Date of Publication: Jan 2006

Durand F., Camdessanche J.-P., Jomir L., Antoine J.-C., Cathebras P. Myasthenia in elderly patients: A series of 23 cases. [Journal: Article] Revue de Medecine Interne. 26(12)(pp 924-930), 2005. Date of Publication: Dec 2005.

Emmerich J. Current state and perspective on medical treatment of critical leg ischemia: Gene and cell therapy. [Journal: Review] International Journal of Lower Extremity Wounds. 4(4)(pp 234-241), 2005. Date of Publication: Dec 2005.

Green D.M. Weakness in the ICU: Guillain-Barre syndrome, myasthenia gravis, critical illness polyneuropathy/myopathy. [Journal: Article] Neurologist. 11(6)(pp 338-347), 2005. Date of Publication: Nov 2005.

Mega R., Coelho F., Pimentel T., Ruiribeiro, De Matos N., Araujo A. Thymus surgery in a general surgery department. [Journal: Article] Acta Medica Portuguesa. 18(4)(pp 267-270), 2005. Date of Publication: Jul 2005.

Komine K., Kohno Y. Histopathological examination of thymic gland - With special reference to myasthenia gravis. [Journal: Article] Tokyo Jikeikai Medical Journal. 120(2)(pp 81-90), 2005. Date of Publication: 15 Mar 2005.

Shahrizaila N., Pacheco O.A., Vidal D.G., Miyares F.R., Wills A.J. Thymectomy in myasthenia gravis: Comparison of outcome in Santiago, Cuba and Nottingham, UK. [Journal: Article] Journal of Neurology. 252(10)(pp 1262-1266), 2005. Date of Publication: Oct 2005.

Horowitz B.Z. Botulinum Toxin. [Journal: Article] Critical Care Clinics. 21(4)(pp 825-839), 2005. Date of Publication: Oct 2005.

Passalacqua S., Staffolani E., Busnach G., Roccatello D., Pasquali S., Cappelli P., Liuzzo G. The Italian Registry for therapeutic apheresis. A report from the Apheresis Study Group of the Italian Society of Nephrology. [Journal: Conference Paper] Journal of Clinical Apheresis. 20(2)(pp 101-106), 2005. Date of Publication: 2005.

Roppolo L.P., Walters K. Airway management in neurological emergencies. [Journal: Review] Neurocritical Care. 1(4)(pp 405-414), 2004. Date of Publication: 2004.

Stickler D.E., Massey J.M., Sanders D.B. MuSK-antibody positive myasthenia gravis: Clinical and electrodiagnostic patterns. [Journal: Review] Clinical Neurophysiology. 116(9)(pp 2065-2068), 2005. Date of Publication: Sep 2005.

Hanly J.G., Harrison M.J. Management of neuropsychiatric lupus. [Journal: Review] Best Practice and Research in Clinical Rheumatology. 19(5 SPEC. ISS.)(pp 799-821), 2005. Date of Publication: Oct 2005.

Mazzorali E., Moshous D., Forino C., De Martiis D., Offer C., Lanfranchi A., Giliani S., Imberti L., Pasic S., Ugazio A.G., Porta F., Notarangelo L.D. Hematopoietic stem cell transplantation in Omenn syndrome: A single-center experience. [Journal: Article] Bone Marrow Transplantation. 36(2)(pp 107-114), 2005. Date of Publication: Jul 2005.

Ben Simon G.J., McCann J.D. Benign essential blepharospasm. [Journal: Review] International Ophthalmology Clinics. 45(3)(pp 49-75), 2005. Date of Publication: Jun 2005.

Ratajczak H.V. Drug-induced hypersensitivity: Role in drug development. [Journal: Review] Toxicological Reviews. 23(4)(pp 265-280), 2004. Date of Publication: 2004.

Huang C.-S., Hsu H.-S., Huang B.-S., Lee H.-C., Kao K.-P., Hsu W.-H., Huang M.-H. Factors influencing the outcome of transsternal thymectomy for myasthenia gravis. [Journal: Review] Acta Neurologica Scandinavica. 112(2)(pp 108-114), 2005. Date of Publication: Aug 2005.

Beeson D., Hantai D., Lochmuller H., Engel A.G. 126th International Workshop: Congenital Myasthenic Syndromes, 24-26 September 2004, Naarden, the Netherlands. [Journal: Conference Paper] Neuromuscular Disorders. 15(7)(pp 498-512), 2005. Date of Publication: Jul 2005.

Zhang X., Qiao J., Lu C.-Z. Serum ryanodine receptor antibody on the assessment of clinical symptoms in patients with myasthenia gravis. [Journal: Article] Chinese Journal of Clinical Rehabilitation. 9(5)(pp 215-217), 2005. Date of Publication: Feb 2005.

Nobile-Orazio E. Treatment of dysimmune neuropathies. [Journal: Review] Journal of Neurology. 252(4)(pp 385-395), 2005. Date of Publication: Apr 2005.

Shah S., Vervan M. Use of i.v. immune globulin and occurrence of associated acute renal failure and thrombosis. [Journal: Review] American Journal of Health-System Pharmacy. 62(7)(pp 720-725), 2005. Date of Publication: 01 Apr 2005

Hutter H.-P., Wallner P., Moshammer H., Hartl W., Sattelberger R., Lorbeer G., Kundi M. Blood concentrations of polycyclic musks in healthy young adults. [Journal: Article] Chemosphere. 59(4)(pp 487-492), 2005. Date of Publication: Apr 2005.

Orlikowski D., Prigent H., Raphael J.-C., Sharshar T. Acute respiratory failure in Guillain-Barre syndrome and myasthenia gravis. [Journal: Article] Reanimation. 14(2)(pp 118-125), 2005. Date of Publication: Mar 2005.

Walsh R.J., Amato A.A. Toxic myopathies. [Journal: Review] Neurologic Clinics. 23(2)(pp 397-428), 2005. Date of Publication: May 2005.

Strommen J.A., Johns J.S., Kim C.-T., Williams F.H., Weiss L.D., Weiss J.M., Rashbaum I.G. Neuromuscular rehabilitation and electrodiagnosis. 3. Diseases of muscles and neuromuscular junction. [Journal: Article] Archives of Physical Medicine and Rehabilitation. 86(3 SUPPL. 2)(pp S18-S27), 2005. Date of Publication: Mar 2005.

Pit'ha J., Dolezil D., Novakova I., Simkova L. First experience with mycophenolate mofetil therapy for refractory myasthenia gravis. [Journal: Article] Ceska a Slovenska Neurologie a Neurochirurgie. 68(1)(pp 4-8), 2005. Date of Publication: 2005.

Nagayasu T., Yamayoshi T., Matsumoto K., Ide N., Hashizume S., Nomura M., Muraoka M., Tagawa T., Akamine S., Oka T. Beneficial effects of plasmapheresis before thymectomy on the outcome in myasthenia gravis. [Journal: Article] Japanese Journal of Thoracic and Cardiovascular Surgery. 53(1)(pp 2-7), 2005. Date of Publication: Jan 2005.

Sieradzan K.A. Wound botulism. [Journal: Article] Practical Neurology. 5(1)(pp 46-51), 2005. Date of Publication: Feb 2005.

White R.D. Hyperthyroidism: Current standards of care. [Journal: Review] Consultant. 44(8)(pp 1085-1090), 2004. Date of Publication: Jul 2004.

Qureshi A.I., Choundry M.A., Mohammad Y., Chua H.C., Yahia A.M., Ulatowski J.A., Krendel D.A., Leshner R.T. Respiratory failure as a first presentation of myasthenia gravis. [Journal: Review] Medical Science Monitor. 10(12)(pp CR684-CR689), 2004. Date of Publication: Dec 2004.

Govindaiah R.C., Sanders G.M. Is hoarseness a problem in your patients with asthma?. [Journal: Note] Journal of Respiratory Diseases. 25(11)(pp 462-463), 2004. Date of Publication: Nov 2004.

Pavic M., Sve P., Malcus C., Sarrot-Reynault F., Peyramond D., Debourdeau P., Andriamanantena D., Bouhour D., Philippe N., Rousset H., Broussolle C. Common variable immunodeficiency with autoimmune manifestations: Study of nine cases; Interest of a peripheral B-cell compartment analysis in seven patients. [Journal: Article] Revue de Medecine Interne. 26(2)(pp 95-102), 2005. Date of Publication: Feb 2005

Glinjongol C., Paiboonpol S. Outcome after transsternal radical thymectomy for myasthenia gravis: 14-year review at Ratchaburi Hospital. [Journal: Review] Journal of the Medical Association of Thailand. 87(11)(pp 1304-1310), 2004. Date of Publication: Nov 2004.

File Jr. T.M. Telithromycin new product overview. [Journal: Review] Journal of Allergy and Clinical Immunology. 115(2)(pp S2-S13), 2005. Date of Publication: Feb 2005.

Vincent A.C., McConville J., Newsom-Davis J., Lindstrom J.M., Engel A.G. Is "seronegative" MG explained by autoantibodies to MuSK? [1] (multiple letters). [Journal: Letter] Neurology. 64(2)(pp 399), 2005. Date of Publication: 25 Jan 2005.

Panda S., Goyal V., Behari M., Singh S., Srivastava T. Myasthenic crisis: A retrospective study. [Journal: Article] Neurology India. 52(4)(pp 453-456), 2004. Date of Publication: Dec 2004.

Oshima M., Deitiker P.R., Mosier D.R., Smith R.G., Atassi M.Z. Responses in vitro of peripheral blood lymphocytes from patients with myasthenia gravis to stimulation with human acetylcholine receptor alpha-chain peptides: Analysis in relation to age, thymic abnormality, and ethnicity. [Journal: Article] Human Immunology. 66(1)(pp 32-42), 2005. Date of Publication: Jan 2005.

Thanvi B.R., Lo T.C.N. Update on myasthenia gravis. [Journal: Review] Postgraduate Medical Journal. 80(950)(pp 690-700), 2004. Date of Publication: Dec 2004.

Aztiria E., Gotti C., Domenici L. alpha7 but not alpha4 AChR subunit expression is regulated by light in developing primary visual cortex. [Journal: Article] Journal of Comparative Neurology. 480(4)(pp 378-391), 2004. Date of Publication: 20 Dec 2004

Mulhall B.P., Younossi Z.M. Nonalcoholic steatohepatitis. [Journal: Article] Current Treatment Options in Gastroenterology. 7(6)(pp 423-430), 2004. Date of Publication: Dec 2004

Liozon E., Loustaud-Ratti V., Soria P., Bezanahary H., Fauchais A.-L., Nadalon S., Rhaiem K., Ly K., Vidal E. Disease associations in 250 patients with temporal (giant cell) arteritis. [Journal: Article] Presse Medicale. 33(19 I)(pp 1304-1312), 2004. Date of Publication: 06 Nov 2004.

Hassan A., Masood F. Wilson's disease: A review. [Journal: Review] Journal of the Pakistan Medical Association. 54(9)(pp 479-484), 2004. Date of Publication: Sep 2004

Murai H., Osoegawa M., Ochi H., Kira J.-I. High frequency of allergic conjunctivitis in myasthenia gravis without thymoma. [Journal: Article] Journal of the Neurological Sciences. 225(1-2)(pp 27-31), 2004. Date of Publication: 15 Oct 2004.

Barwick R. History of thymoma and yellow fever vaccination [2]. [Journal: Letter] Lancet. 364(9438)(pp 936), 2004. Date of Publication: 11 Sep 2004.

Donmez B., Ozakbas S., Oktem M.A., Gedizlioglu M., Coker I., Genc A., Idiman E. HLA genotypes in Turkish patients with myasthenia gravis: Comparison with multiple sclerosis patients on the basis of clinical subtypes and demographic features. [Journal: Article] Human Immunology. 65(7)(pp 752-757), 2004. Date of Publication: Jul 2004.

Zielinski M., Kuzdzal J., Staniec B., Harazda M., Nabialek T., Pankowski J., Szlubowski A., Medon J. Safety for preoperative use of steroids for transsternal thymectomy in myasthenia gravis. [Journal: Conference Paper] European Journal of Cardio-thoracic Surgery. 26(2)(pp 407-411), 2004. Date of Publication: Aug 2004.

Zielinski M., Kuzdzal J., Szlubowski A., Soja J. Transcervical-subxiphoid-videothoracoscopic "maximal" thymectomy - Operative technique and early results. [Journal: Article] Annals of Thoracic Surgery. 78(2)(pp 404-409), 2004. Date of Publication: Aug 2004.

Wirtz P.W., Van Dijk J.G., Van Doorn P.A., Van Engelen B.G.M., Van Der Kooi A.J., Kuks J.B., Twijnstra A., De Visser M., Visser L.H., Wokke J.H., Wintzen A.R., Verschuuren J.J. The epidemiology of the Lambert-Eaton myasthenic syndrome in the Netherlands. [Journal: Article] Neurology. 63(2)(pp 397-398), 2004. Date of Publication: 27 Jul 2004.

Carandina-Maffeis R., Nucci A., Marques Jr. J.F.C., Roveri E.G., Pfeilsticker B.H.M., Garibaldi S.G., De Deus-Silva L. Plasmapheresis in the treatment of myasthenia gravis: Retrospective study of 26 patients. [Journal: Article] Arquivos de Neuro-Psiquiatria. 62(2 B)(pp 391-395), 2004. Date of Publication: Jun 2004

Kadoi Y., Hinohara H., Kunimoto F., Niijima A., Saito S., Goto F. Is the degree of sensitivity to nondepolarizing muscle relaxants related to requirements for postoperative ventilation in patients with myasthenia gravis?. [Journal: Article] Anaesthesia and Intensive Care. 32(3)(pp 346-350), 2004. Date of Publication: Jun 2004.

Arai M., Ukigai H., Miyata H. A case of transient left ventricular ballooning ("Takotsubo"- shaped cardiomyopathy) developed during plasmapheresis for treatment of myasthenic crisis. [Journal: Article] Clinical Neurology. 44(3)(pp 207-210), 2004. Date of Publication: Mar 2004

Huang C.-M., Yang Y.-H., Chiang B.-L. Different familial association patterns of autoimmune diseases between juvenile-onset systemic lupus erythematosus and juvenile rheumatoid arthritis. [Journal: Article] Journal of Microbiology, Immunology and Infection. 37(2)(pp 88-94), 2004. Date of Publication: Apr 2004.

Juel V.C. Myasthenia gravis: Management of myasthenic crisis and perioperative care. [Journal: Review] Seminars in Neurology. 24(1)(pp 75-81), 2004. Date of Publication: Mar 2004.

Jaretzki III A., Steinglass K.M., Sonett J.R. Thymectomy in the management of myasthenia gravis. [Journal: Review] Seminars in Neurology. 24(1)(pp 49-62), 2004. Date of Publication: Mar 2004.

Campbell M.L. Terminal dyspnea and respiratory distress. [Journal: Review] Critical Care Clinics. 20(3)(pp 403-417), 2004. Date of Publication: Jul 2004.

Clark W., Jobanputra P., Barton P., Burls A. The clinical and cost-effectiveness of anakinra for the treatment of rheumatoid arthritis in adults: A systematic review and economic analysis. [Journal: Review] Health Technology Assessment. 8(18)(pp iii-75), 2004. Date of Publication: May 2004.

Guo X.-H., Wu W.-P., Zhang Y.-H., Shi H.-Y., Zhu K. Clinical study on the relationship between the pathology of thymoma and myasthenia gravis. [Journal: Article] Chinese Journal of Clinical Rehabilitation. 7(16)(pp 2332-2333), 2003. Date of Publication: Jul 2003.

Zielinski M., Kuzdzal J., Staniec B., Harazda M., Nabialek T., Pankowski J., Szlubowski A., Narski M. Extended rethymectomy in the treatment of refractory myasthenia gravis: Original video-assisted technique of resternotomy and results of the treatment in 21 patients. [Journal: Conference Paper] Interactive

Friedburg D., Pau H. Opthalmology with children. 13. External eye muscles. [Journal: Article] Padiatrische Praxis. 64(4)(pp 615-630), 2004. Date of Publication: May 2004.

Corbin D. Barbados. [Journal: Note] Practical Neurology. 4(2)(pp 120-121), 2004. Date of Publication: Apr 2004.

Guglin M., Campellone J.V., Heintz K., Parrillo J.E. Cardiac disease in myasthenia gravis: A literature review. [Journal: Review] Journal of Clinical Neuromuscular Disease. 4(4)(pp 199-203), 2003. Date of Publication: Jun 2003

Koc F., Yerdelen D., Sarica Y. Myasthenia gravis and invasive thymoma with multiple intracranial metastases. [Journal: Article] Journal of Clinical Neuromuscular Disease. 4(4)(pp 171-173), 2003. Date of Publication: Jun 2003.

Oliveira E., Michel A., Smolley L. The pulmonary consultation in the perioperative management of patients with neurologic diseases. [Journal: Review] Neurologic Clinics. 22(2)(pp 277-291), 2004. Date of Publication: May 2004.

.

Farag N.H., Mills P.J. A randomized-controlled trial of the effects of a traditional herbal supplement on sleep onset insomia. [Journal: Article] Complementary Therapies in Medicine. 11(4)(pp 223-225), 2003. Date of Publication: Dec 2003.

Singh R.S., Behera S.K., Saji R., Dhaliwal R.S. Thymectomy for myasthenia gravis: 12-year experience. [Journal: Article] Asian Cardiovascular and Thoracic Annals. 11(4)(pp 299-303), 2003. Date of Publication: Dec 2003.

Utzinger J., Keiser J. Schistosomiasis and soil-transmitted helminthiasis: Common drugs for treatment and control. [Journal: Review] Expert Opinion on Pharmacotherapy. 5(2)(pp 263-285), 2004. Date of Publication: Feb 2004.

Happe S. Excessive Daytime Sleepiness and Sleep Disturbances in Patients with Neurological Diseases: Epidemiology and Management. [Journal: Review] Drugs. 63(24)(pp 2725-2737), 2003. Date of Publication: 2003.

Vincent A., Clover L., Buckley C., Grimley Evans J., Rothwell P.M. Evidence of underdiagnosis of myasthenia gravis in older people. [Journal: Article] Journal of Neurology Neurosurgery and Psychiatry. 74(8)(pp 1105-1108), 2003. Date of Publication: 01 Aug 2003.

Guidetti D., Sabadini R., Bondavalli M., Cavalletti S., Lodesani M., Mantegazza R., Cosi V F., Solime Epidemiological study of myasthenia gravis in the province of Reggio Emilia, Italy. [Journal: Article] European Journal of Epidemiology. 14(4)(pp 381-387), 1998. Date of Publication: 1998.

Christensen P.B., Jensen T.S., Tsiropoulos I., Sorensen T., Kjaer M., Hojer-Pedersen E., Rasmussen M.J.K., Lehfeldt E. Mortality and survival in myasthenia gravis: A Danish population based study. [Journal: Article] Journal of Neurology Neurosurgery and Psychiatry. 64(1)(pp 78-83), 1998. Date of Publication: Jan 1998.

### PUBMED (first 250 hits listed only)

# [Gastrointestinal stromal tumors: a demographic, morphologic and immunohistochemical study.](http://www.ncbi.nlm.nih.gov/pubmed/18227570?ordinalpos=52&itool=EntrezSystem2.PEntrez.Pubmed.Pubmed_ResultsPanel.Pubmed_DefaultReportPanel.Pubmed_RVDocSum)

Rauf F, Bhurgri Y, Pervez S.

Indian J Gastroenterol. 2007 Sep-Oct;26(5):214-6.

PMID: 18227570 [PubMed - indexed for MEDLINE]

# [Clinical and pathological aspects of thymic epithelial tumors.](http://www.ncbi.nlm.nih.gov/pubmed/18213465?ordinalpos=53&itool=EntrezSystem2.PEntrez.Pubmed.Pubmed_ResultsPanel.Pubmed_DefaultReportPanel.Pubmed_RVDocSum)

Okumura M, Shiono H, Minami M, Inoue M, Utsumi T, Kadota Y, Sawa Y.

Gen Thorac Cardiovasc Surg. 2008 Jan;56(1):10-6. Epub 2008 Jan 22. Review.

PMID: 18213465 [PubMed - indexed for MEDLINE]

# [Noninvasive ventilation in myasthenic crisis.](http://www.ncbi.nlm.nih.gov/pubmed/18195139?ordinalpos=54&itool=EntrezSystem2.PEntrez.Pubmed.Pubmed_ResultsPanel.Pubmed_DefaultReportPanel.Pubmed_RVDocSum)

Seneviratne J, Mandrekar J, Wijdicks EF, Rabinstein AA.

Arch Neurol. 2008 Jan;65(1):54-8.

PMID: 18195139 [PubMed - indexed for MEDLINE]

# [Musculoskeletal complications of neuromuscular disease in children.](http://www.ncbi.nlm.nih.gov/pubmed/18194756?ordinalpos=55&itool=EntrezSystem2.PEntrez.Pubmed.Pubmed_ResultsPanel.Pubmed_DefaultReportPanel.Pubmed_RVDocSum)

Driscoll SW, Skinner J.

Phys Med Rehabil Clin N Am. 2008 Feb;19(1):163-94, viii. Review.

PMID: 18194756 [PubMed - indexed for MEDLINE]

# [Myasthenia gravis crisis.](http://www.ncbi.nlm.nih.gov/pubmed/18176295?ordinalpos=57&itool=EntrezSystem2.PEntrez.Pubmed.Pubmed_ResultsPanel.Pubmed_DefaultReportPanel.Pubmed_RVDocSum)

Bershad EM, Feen ES, Suarez JI.

South Med J. 2008 Jan;101(1):63-9. Review.

PMID: 18176295 [PubMed - indexed for MEDLINE]

[Related Articles](http://www.ncbi.nlm.nih.gov/sites/entrez?db=pubmed&cmd=link&linkname=pubmed_pubmed&uid=18176295&ordinalpos=57:)

# [Novel prognostic groups in thymic epithelial tumors: assessment of risk and therapeutic strategy selection.](http://www.ncbi.nlm.nih.gov/pubmed/18164843?ordinalpos=58&itool=EntrezSystem2.PEntrez.Pubmed.Pubmed_ResultsPanel.Pubmed_DefaultReportPanel.Pubmed_RVDocSum)

D'Angelillo RM, Trodella L, Ramella S, Cellini N, Balducci M, Mantini G, Cellini F, Ciresa M, Fiore M, Evoli A, Sterzi S, Russo P, Grozio A, Cesario A, Granone P.

Int J Radiat Oncol Biol Phys. 2008 Jun 1;71(2):420-7. Epub 2007 Dec 31.

PMID: 18164843 [PubMed - indexed for MEDLINE]

# [[Long-term results of surgical treatment of thymomas at the patients with generalized myasthenia]](http://www.ncbi.nlm.nih.gov/pubmed/18163050?ordinalpos=59&itool=EntrezSystem2.PEntrez.Pubmed.Pubmed_ResultsPanel.Pubmed_DefaultReportPanel.Pubmed_RVDocSum)

Shevchenko IuL, Vetshev PS, Sanadze AG, Ippolitov LI, Sidnev DV, Abdalova OV, Fat'ianova AS.

Khirurgiia (Mosk). 2007;(10):36-43. Russian.

PMID: 18163050 [PubMed - indexed for MEDLINE]

# [Cancer incidence among women and girls environmentally and occupationally exposed to blue asbestos at Wittenoom, Western Australia.](http://www.ncbi.nlm.nih.gov/pubmed/18098288?ordinalpos=60&itool=EntrezSystem2.PEntrez.Pubmed.Pubmed_ResultsPanel.Pubmed_DefaultReportPanel.Pubmed_RVDocSum)

Reid A, Heyworth J, de Klerk NH, Musk B.

Int J Cancer. 2008 May 15;122(10):2337-44.

# [Electrophysiological signs and the prevalence of adverse effects of acetylcholinesterase inhibitors in patients with myasthenia gravis.](http://www.ncbi.nlm.nih.gov/pubmed/18069667?ordinalpos=61&itool=EntrezSystem2.PEntrez.Pubmed.Pubmed_ResultsPanel.Pubmed_DefaultReportPanel.Pubmed_RVDocSum)

Punga AR, Sawada M, Stålberg EV.

Muscle Nerve. 2008 Mar;37(3):300-7.

PMID: 18069667 [PubMed - indexed for MEDLINE]

# [Lifetime course of myasthenia gravis.](http://www.ncbi.nlm.nih.gov/pubmed/18059039?ordinalpos=62&itool=EntrezSystem2.PEntrez.Pubmed.Pubmed_ResultsPanel.Pubmed_DefaultReportPanel.Pubmed_RVDocSum)

Grob D, Brunner N, Namba T, Pagala M.

Muscle Nerve. 2008 Feb;37(2):141-9. Review.

PMID: 18059039 [PubMed - indexed for MEDLINE]

[Related Articles](http://www.ncbi.nlm.nih.gov/sites/entrez?db=pubmed&cmd=link&linkname=pubmed_pubmed&uid=18059039&ordinalpos=62:)

# [[Surgery of mediastinal tumors]](http://www.ncbi.nlm.nih.gov/pubmed/18058077?ordinalpos=63&itool=EntrezSystem2.PEntrez.Pubmed.Pubmed_ResultsPanel.Pubmed_DefaultReportPanel.Pubmed_RVDocSum)

Stremmel C, Passlick B.

Chirurg. 2008 Jan;79(1):9-10, 12-7. Review. German.

PMID: 18058077 [PubMed - indexed for MEDLINE]

[Related Articles](http://www.ncbi.nlm.nih.gov/sites/entrez?db=pubmed&cmd=link&linkname=pubmed_pubmed&uid=18058077&ordinalpos=63:)

# [Mortality of former crocidolite (blue asbestos) miners and millers at Wittenoom.](http://www.ncbi.nlm.nih.gov/pubmed/18045848?ordinalpos=64&itool=EntrezSystem2.PEntrez.Pubmed.Pubmed_ResultsPanel.Pubmed_DefaultReportPanel.Pubmed_RVDocSum)

Musk AW, de Klerk NH, Reid A, Ambrosini GL, Fritschi L, Olsen NJ, Merler E, Hobbs MS, Berry G.

Occup Environ Med. 2008 Aug;65(8):541-3. Epub 2007 Nov 28.

PMID: 18045848 [PubMed - indexed for MEDLINE]

[Related Articles](http://www.ncbi.nlm.nih.gov/sites/entrez?db=pubmed&cmd=link&linkname=pubmed_pubmed&uid=18045848&ordinalpos=64:)

# [Clinical neurophysiology of fatigue.](http://www.ncbi.nlm.nih.gov/pubmed/18039594?ordinalpos=65&itool=EntrezSystem2.PEntrez.Pubmed.Pubmed_ResultsPanel.Pubmed_DefaultReportPanel.Pubmed_RVDocSum)

Zwarts MJ, Bleijenberg G, van Engelen BG.

Clin Neurophysiol. 2008 Jan;119(1):2-10. Epub 2007 Nov 26. Review.

PMID: 18039594 [PubMed - indexed for MEDLINE]

# [[Relevance of plasma exchange in the treatment of myasthenia gravis: study of 11 cases]](http://www.ncbi.nlm.nih.gov/pubmed/18035453?ordinalpos=66&itool=EntrezSystem2.PEntrez.Pubmed.Pubmed_ResultsPanel.Pubmed_DefaultReportPanel.Pubmed_RVDocSum)

Miladi MI, Feki I, Kammoun H, Elleuch H, Triki C, Gargouri J, Mhiri C.

Rev Med Interne. 2008 Feb;29(2):87-93. Epub 2007 Oct 25. French.

PMID: 18035453 [PubMed - indexed for MEDLINE]

[Related Articles](http://www.ncbi.nlm.nih.gov/sites/entrez?db=pubmed&cmd=link&linkname=pubmed_pubmed&uid=18035453&ordinalpos=66:)[Related Articles](http://www.ncbi.nlm.nih.gov/sites/entrez?db=pubmed&cmd=link&linkname=pubmed_pubmed&uid=18000579&ordinalpos=67:)

# [Myasthenia gravis.](http://www.ncbi.nlm.nih.gov/pubmed/17986328?ordinalpos=68&itool=EntrezSystem2.PEntrez.Pubmed.Pubmed_ResultsPanel.Pubmed_DefaultReportPanel.Pubmed_RVDocSum)

Juel VC, Massey JM.

Orphanet J Rare Dis. 2007 Nov 6;2:44. Review.

PMID: 17986328 [PubMed - indexed for MEDLINE]

[Related Articles](http://www.ncbi.nlm.nih.gov/sites/entrez?db=pubmed&cmd=link&linkname=pubmed_pubmed&uid=17986328&ordinalpos=68:) [Free article in PMC](http://www.ncbi.nlm.nih.gov/pubmed/17986328?ordinalpos=68&itool=EntrezSystem2.PEntrez.Pubmed.Pubmed_ResultsPanel.Pubmed_DefaultReportPanel.Pubmed_RVDocSum&log$=freepmc)

# [Mediastinal thymolipoma: an analysis of 10 cases.](http://www.ncbi.nlm.nih.gov/pubmed/17986126?ordinalpos=69&itool=EntrezSystem2.PEntrez.Pubmed.Pubmed_ResultsPanel.Pubmed_DefaultReportPanel.Pubmed_RVDocSum)

Damadoglu E, Salturk C, Takir HB, Ertugrul M, Yilmaz A, Atasalihi A, Yilmaz A.

Respirology. 2007 Nov;12(6):924-7.

PMID: 17986126 [PubMed - indexed for MEDLINE]

# [Pain in hereditary neuromuscular disorders and myasthenia gravis: a national survey of frequency, characteristics, and impact.](http://www.ncbi.nlm.nih.gov/pubmed/17981001?ordinalpos=70&itool=EntrezSystem2.PEntrez.Pubmed.Pubmed_ResultsPanel.Pubmed_DefaultReportPanel.Pubmed_RVDocSum)

Guy-Coichard C, Nguyen DT, Delorme T, Boureau F.

J Pain Symptom Manage. 2008 Jan;35(1):40-50. Epub 2007 Nov 5.

PMID: 17981001 [PubMed - indexed for MEDLINE]

# [Looking for the best management of myasthenia gravis at the Tg.Mureş Clinic of Anesthesia and Intensive Care.](http://www.ncbi.nlm.nih.gov/pubmed/17966935?ordinalpos=71&itool=EntrezSystem2.PEntrez.Pubmed.Pubmed_ResultsPanel.Pubmed_DefaultReportPanel.Pubmed_RVDocSum)

Copotoiu SM, Copotoiu C, Bud V, Molnar C, Azamfirei L, Ghitescu I, Măruşteri M, Dogaru M, Brânzaniuc K.

Chirurgia (Bucur). 2007 Jul-Aug;102(4):401-5.

PMID: 17966935 [PubMed - indexed for MEDLINE]

# [[Primary cysts and tumors of the mediastinum]](http://www.ncbi.nlm.nih.gov/pubmed/17962885?ordinalpos=72&itool=EntrezSystem2.PEntrez.Pubmed.Pubmed_ResultsPanel.Pubmed_DefaultReportPanel.Pubmed_RVDocSum)

Bastos P, Magalhães A, Fernandes G, Cruz MR, Saleiro S, Gonçalves L, Piñon M.

Rev Port Pneumol. 2007 Sep-Oct;13(5):659-73. Portuguese.

PMID: 17962885 [PubMed - indexed for MEDLINE]

# [A new mouse model of autoimmune ocular myasthenia gravis.](http://www.ncbi.nlm.nih.gov/pubmed/17962462?ordinalpos=73&itool=EntrezSystem2.PEntrez.Pubmed.Pubmed_ResultsPanel.Pubmed_DefaultReportPanel.Pubmed_RVDocSum)

Yang H, Wu B, Tüzün E, Saini SS, Li J, Allman W, Higgs S, Xiao TL, Christadoss P.

Invest Ophthalmol Vis Sci. 2007 Nov;48(11):5101-11.

PMID: 17962462 [PubMed - indexed for MEDLINE]

# [Clinical features of patients with myasthenia gravis associated with autoimmune diseases.](http://www.ncbi.nlm.nih.gov/pubmed/17941854?ordinalpos=74&itool=EntrezSystem2.PEntrez.Pubmed.Pubmed_ResultsPanel.Pubmed_DefaultReportPanel.Pubmed_RVDocSum)

Kanazawa M, Shimohata T, Tanaka K, Nishizawa M.

Eur J Neurol. 2007 Dec;14(12):1403-4. Epub 2007 Oct 17.

PMID: 17941854 [PubMed - indexed for MEDLINE]

# [Neuromuscular diseases in pregnancy.](http://www.ncbi.nlm.nih.gov/pubmed/17940925?ordinalpos=75&itool=EntrezSystem2.PEntrez.Pubmed.Pubmed_ResultsPanel.Pubmed_DefaultReportPanel.Pubmed_RVDocSum)

Briemberg HR.

Semin Neurol. 2007 Nov;27(5):460-6. Review.

PMID: 17940925 [PubMed - indexed for MEDLINE]

# [[Plasma exchange and immunoadsorption]](http://www.ncbi.nlm.nih.gov/pubmed/17939280?ordinalpos=76&itool=EntrezSystem2.PEntrez.Pubmed.Pubmed_ResultsPanel.Pubmed_DefaultReportPanel.Pubmed_RVDocSum)

Dittrich E, Schmaldienst S, Derfler K.

Wien Klin Wochenschr. 2007;119(5-6 Suppl 1):39-53; quiz 54. Review. German. No abstract available.

PMID: 17939280 [PubMed - indexed for MEDLINE]

# [Outcome of myasthenia gravis mothers and their infants.](http://www.ncbi.nlm.nih.gov/pubmed/17912986?ordinalpos=77&itool=EntrezSystem2.PEntrez.Pubmed.Pubmed_ResultsPanel.Pubmed_DefaultReportPanel.Pubmed_RVDocSum)

Cheng I, Lin CH, Lin MI, Lee JS, Chiu HC, Mu SC.

Acta Paediatr Taiwan. 2007 May-Jun;48(3):141-5.

PMID: 17912986 [PubMed - indexed for MEDLINE]

[Related Articles](http://www.ncbi.nlm.nih.gov/sites/entrez?db=pubmed&cmd=link&linkname=pubmed_pubmed&uid=17912986&ordinalpos=77:)

# [Thymectomy for myasthenia gravis: prognostic factors in 70 patients.](http://www.ncbi.nlm.nih.gov/pubmed/17911062?ordinalpos=78&itool=EntrezSystem2.PEntrez.Pubmed.Pubmed_ResultsPanel.Pubmed_DefaultReportPanel.Pubmed_RVDocSum)

Aghajanzadeh M, Khoshrang H, Mohammadzadeh A, Roudbari SA, Ghayeghran AR.

Asian Cardiovasc Thorac Ann. 2007 Oct;15(5):371-5.

PMID: 17911062 [PubMed - indexed for MEDLINE]

[Related Articles](http://www.ncbi.nlm.nih.gov/sites/entrez?db=pubmed&cmd=link&linkname=pubmed_pubmed&uid=17911062&ordinalpos=78:)

# [Neuromuscular disease as the cause of late clubfoot relapses: report of 4 cases.](http://www.ncbi.nlm.nih.gov/pubmed/17907435?ordinalpos=79&itool=EntrezSystem2.PEntrez.Pubmed.Pubmed_ResultsPanel.Pubmed_DefaultReportPanel.Pubmed_RVDocSum)

Lovell ME, Morcuende JA.

Iowa Orthop J. 2007;27:82-4.

PMID: 17907435 [PubMed - indexed for MEDLINE]

[Related Articles](http://www.ncbi.nlm.nih.gov/sites/entrez?db=pubmed&cmd=link&linkname=pubmed_pubmed&uid=17907435&ordinalpos=79:) [Free article in PMC](http://www.ncbi.nlm.nih.gov/pubmed/17907435?ordinalpos=79&itool=EntrezSystem2.PEntrez.Pubmed.Pubmed_ResultsPanel.Pubmed_DefaultReportPanel.Pubmed_RVDocSum&log$=freepmc)

# [[Thymoma--incidence, classification and therapy]](http://www.ncbi.nlm.nih.gov/pubmed/17899505?ordinalpos=80&itool=EntrezSystem2.PEntrez.Pubmed.Pubmed_ResultsPanel.Pubmed_DefaultReportPanel.Pubmed_RVDocSum)

Stremmel C, Dango S, Thiemann U, Kayser G, Passlick B.

Dtsch Med Wochenschr. 2007 Oct;132(40):2090-5. Review. German.

PMID: 17899505 [PubMed - indexed for MEDLINE]

# [Polymorphisms in the cathepsin L2 (CTSL2) gene show association with type 1 diabetes and early-onset myasthenia gravis.](http://www.ncbi.nlm.nih.gov/pubmed/17869649?ordinalpos=81&itool=EntrezSystem2.PEntrez.Pubmed.Pubmed_ResultsPanel.Pubmed_DefaultReportPanel.Pubmed_RVDocSum)

Viken MK, Sollid HD, Joner G, Dahl-Jørgensen K, Rønningen KS, Undlien DE, Flatø B, Selvaag AM, Førre Ø, Kvien TK, Thorsby E, Melms A, Tolosa E, Lie BA.

Hum Immunol. 2007 Sep;68(9):748-55. Epub 2007 Jun 28.

PMID: 17869649 [PubMed - indexed for MEDLINE]

# [Transfusion related acute lung injury (TRALI) after plasma exchange in myasthenic crisis.](http://www.ncbi.nlm.nih.gov/pubmed/17805491?ordinalpos=82&itool=EntrezSystem2.PEntrez.Pubmed.Pubmed_ResultsPanel.Pubmed_DefaultReportPanel.Pubmed_RVDocSum)

Mateen FJ, Gastineau D.

Neurocrit Care. 2008;8(2):280-2.

PMID: 17805491 [PubMed - indexed for MEDLINE]

# [A severe outbreak of contagious ecthyma (orf) in a free-ranging musk ox (Ovibos moschatus) population in Norway.](http://www.ncbi.nlm.nih.gov/pubmed/17768017?ordinalpos=83&itool=EntrezSystem2.PEntrez.Pubmed.Pubmed_ResultsPanel.Pubmed_DefaultReportPanel.Pubmed_RVDocSum)

Vikøren T, Lillehaug A, Akerstedt J, Bretten T, Haugum M, Tryland M.

Vet Microbiol. 2008 Feb 5;127(1-2):10-20. Epub 2007 Aug 2.

PMID: 17768017 [PubMed - indexed for MEDLINE]

# [Adjuvant radiotherapy for thymic epithelial tumor: treatment results and prognostic factors.](http://www.ncbi.nlm.nih.gov/pubmed/17762439?ordinalpos=84&itool=EntrezSystem2.PEntrez.Pubmed.Pubmed_ResultsPanel.Pubmed_DefaultReportPanel.Pubmed_RVDocSum)

Kundel Y, Yellin A, Popovtzer A, Pfeffer R, Symon Z, Simansky DA, Oberman B, Sadezki S, Brenner B, Catane R, Levitt ML.

Am J Clin Oncol. 2007 Aug;30(4):389-94.

PMID: 17762439 [PubMed - indexed for MEDLINE]

# [Myasthenia gravis in South Africans: racial differences in clinical manifestations.](http://www.ncbi.nlm.nih.gov/pubmed/17720497?ordinalpos=85&itool=EntrezSystem2.PEntrez.Pubmed.Pubmed_ResultsPanel.Pubmed_DefaultReportPanel.Pubmed_RVDocSum)

Heckmann JM, Owen EP, Little F.

Neuromuscul Disord. 2007 Dec;17(11-12):929-34. Epub 2007 Aug 27.

PMID: 17720497 [PubMed - indexed for MEDLINE]

[Related Articles](http://www.ncbi.nlm.nih.gov/sites/entrez?db=pubmed&cmd=link&linkname=pubmed_pubmed&uid=17720497&ordinalpos=85:)

# [Clinical and experimental features of MuSK antibody positive MG in Japan.](http://www.ncbi.nlm.nih.gov/pubmed/17718696?ordinalpos=86&itool=EntrezSystem2.PEntrez.Pubmed.Pubmed_ResultsPanel.Pubmed_DefaultReportPanel.Pubmed_RVDocSum)

Ohta K, Shigemoto K, Fujinami A, Maruyama N, Konishi T, Ohta M.

Eur J Neurol. 2007 Sep;14(9):1029-34.

PMID: 17718696 [PubMed - indexed for MEDLINE]

[Related Articles](http://www.ncbi.nlm.nih.gov/sites/entrez?db=pubmed&cmd=link&linkname=pubmed_pubmed&uid=17718696&ordinalpos=86:)

# [Early prediction of response to chemotherapy and survival in malignant pleural mesothelioma using a novel semiautomated 3-dimensional volume-based analysis of serial 18F-FDG PET scans.](http://www.ncbi.nlm.nih.gov/pubmed/17704250?ordinalpos=87&itool=EntrezSystem2.PEntrez.Pubmed.Pubmed_ResultsPanel.Pubmed_DefaultReportPanel.Pubmed_RVDocSum)

Francis RJ, Byrne MJ, van der Schaaf AA, Boucek JA, Nowak AK, Phillips M, Price R, Patrikeos AP, Musk AW, Millward MJ.

J Nucl Med. 2007 Sep;48(9):1449-58. Epub 2007 Aug 17.

PMID: 17704250 [PubMed - indexed for MEDLINE]

[Related Articles](http://www.ncbi.nlm.nih.gov/sites/entrez?db=pubmed&cmd=link&linkname=pubmed_pubmed&uid=17704250&ordinalpos=87:)

# [Neurological care and risk of hospital mortality for patients with myasthenia gravis in England.](http://www.ncbi.nlm.nih.gov/pubmed/17698503?ordinalpos=88&itool=EntrezSystem2.PEntrez.Pubmed.Pubmed_ResultsPanel.Pubmed_DefaultReportPanel.Pubmed_RVDocSum)

Hill M, Ben-Shlomo Y.

J Neurol Neurosurg Psychiatry. 2008 Apr;79(4):421-5. Epub 2007 Aug 13.

PMID: 17698503 [PubMed - indexed for MEDLINE]

# [The effects of odor on cortisol and testosterone in healthy adults.](http://www.ncbi.nlm.nih.gov/pubmed/17693981?ordinalpos=89&itool=EntrezSystem2.PEntrez.Pubmed.Pubmed_ResultsPanel.Pubmed_DefaultReportPanel.Pubmed_RVDocSum)

Fukui H, Komaki R, Okui M, Toyoshima K, Kuda K.

Neuro Endocrinol Lett. 2007 Aug;28(4):433-7.

PMID: 17693981 [PubMed - indexed for MEDLINE]

# [An IRF8-binding promoter variant and AIRE control CHRNA1 promiscuous expression in thymus.](http://www.ncbi.nlm.nih.gov/pubmed/17687331?ordinalpos=90&itool=EntrezSystem2.PEntrez.Pubmed.Pubmed_ResultsPanel.Pubmed_DefaultReportPanel.Pubmed_RVDocSum)

Giraud M, Taubert R, Vandiedonck C, Ke X, Lévi-Strauss M, Pagani F, Baralle FE, Eymard B, Tranchant C, Gajdos P, Vincent A, Willcox N, Beeson D, Kyewski B, Garchon HJ.

Nature. 2007 Aug 23;448(7156):934-7. Epub 2007 Aug 8.

PMID: 17687331 [PubMed - indexed for MEDLINE]

# [Extended rethymectomy in the treatment of refractory myasthenia gravis: original video-assisted technique of resternotomy and results of the treatment in 21 patients.](http://www.ncbi.nlm.nih.gov/pubmed/17670265?ordinalpos=91&itool=EntrezSystem2.PEntrez.Pubmed.Pubmed_ResultsPanel.Pubmed_DefaultReportPanel.Pubmed_RVDocSum)

Zieliński M, Kuzdzał J, Staniec B, Harazda M, Nabiałek T, Pankowski J, Szlubowski A, Narski M.

Interact Cardiovasc Thorac Surg. 2004 Jun;3(2):376-80.

PMID: 17670265 [PubMed]

# [Circular dichroism studies of extracellular domains of human nicotinic acetylcholine receptors provide an insight into their structure.](http://www.ncbi.nlm.nih.gov/pubmed/17659334?ordinalpos=92&itool=EntrezSystem2.PEntrez.Pubmed.Pubmed_ResultsPanel.Pubmed_DefaultReportPanel.Pubmed_RVDocSum)

Zouridakis M, Kostelidou K, Sotiriadis A, Stergiou C, Eliopoulos E, Poulas K, Tzartos SJ.

Int J Biol Macromol. 2007 Oct 1;41(4):423-9. Epub 2007 Jun 8.

PMID: 17659334 [PubMed - indexed for MEDLINE]

# [Diagnosis and treatment of myasthenia gravis.](http://www.ncbi.nlm.nih.gov/pubmed/17658970?ordinalpos=93&itool=EntrezSystem2.PEntrez.Pubmed.Pubmed_ResultsPanel.Pubmed_DefaultReportPanel.Pubmed_RVDocSum)

Matney SE, Huff DR.

Consult Pharm. 2007 Mar;22(3):239-48. Review.

PMID: 17658970 [PubMed - indexed for MEDLINE]

# [CRMP5 antibodies in patients with small-cell lung cancer or thymoma.](http://www.ncbi.nlm.nih.gov/pubmed/17657489?ordinalpos=94&itool=EntrezSystem2.PEntrez.Pubmed.Pubmed_ResultsPanel.Pubmed_DefaultReportPanel.Pubmed_RVDocSum)

Monstad SE, Drivsholm L, Skeie GO, Aarseth JH, Vedeler CA.

Cancer Immunol Immunother. 2008 Feb;57(2):227-32. Epub 2007 Jul 27.

PMID: 17657489 [PubMed - indexed for MEDLINE]

[Related Articles](http://www.ncbi.nlm.nih.gov/sites/entrez?db=pubmed&cmd=link&linkname=pubmed_pubmed&uid=17657489&ordinalpos=94:)

# [Nonthymoma early-onset- and late-onset-generalized myasthenia gravis--a retrospective hospital-based study.](http://www.ncbi.nlm.nih.gov/pubmed/17644246?ordinalpos=96&itool=EntrezSystem2.PEntrez.Pubmed.Pubmed_ResultsPanel.Pubmed_DefaultReportPanel.Pubmed_RVDocSum)

Chan KH, Cheung RT, Mak W, Ho SL.

Clin Neurol Neurosurg. 2007 Oct;109(8):686-91. Epub 2007 Jul 17.

PMID: 17644246 [PubMed - indexed for MEDLINE]

# [[Immunoregulative effect of NK1.1+ cells on the development of experimental autoimmune myasthenia gravis]](http://www.ncbi.nlm.nih.gov/pubmed/17618568?ordinalpos=97&itool=EntrezSystem2.PEntrez.Pubmed.Pubmed_ResultsPanel.Pubmed_DefaultReportPanel.Pubmed_RVDocSum)

Li HL, Wang GY, Li GZ, Sun B, Zhao JC, Kong QF, Jin LH, Wang DD.

Xi Bao Yu Fen Zi Mian Yi Xue Za Zhi. 2007 Aug;23(8):737-40. Chinese.

PMID: 17618568 [PubMed - in process]

# [Telithromycin: the perils of hasty adoption and persistence of off-label prescribing.](http://www.ncbi.nlm.nih.gov/pubmed/17605513?ordinalpos=98&itool=EntrezSystem2.PEntrez.Pubmed.Pubmed_ResultsPanel.Pubmed_DefaultReportPanel.Pubmed_RVDocSum)

Gleason PP, Walters C, Heaton AH, Schafer JA.

J Manag Care Pharm. 2007 Jun;13(5):420-5.

PMID: 17605513 [PubMed - indexed for MEDLINE]

# [[Seric 21-hydroxilase antibodies in patients with anti-microsomal fraction antibodies. Autoimmune polyendocrine syndrome]](http://www.ncbi.nlm.nih.gov/pubmed/17593598?ordinalpos=99&itool=EntrezSystem2.PEntrez.Pubmed.Pubmed_ResultsPanel.Pubmed_DefaultReportPanel.Pubmed_RVDocSum)

Botta S, Roveto S, Rimoldi D.

Medicina (B Aires). 2007;67(2):143-6. Spanish.

PMID: 17593598 [PubMed - indexed for MEDLINE]

# [[Myasthenia gravis: treatments and remissions]](http://www.ncbi.nlm.nih.gov/pubmed/17564346?ordinalpos=100&itool=EntrezSystem2.PEntrez.Pubmed.Pubmed_ResultsPanel.Pubmed_DefaultReportPanel.Pubmed_RVDocSum)

Dunand M, Lalive PH, Vokatch N, Kuntzer T.

Rev Med Suisse. 2007 May 9;3(110):1185-6, 1188-90. Review. French.

PMID: 17564346 [PubMed - indexed for MEDLINE]

[Related Articles](http://www.ncbi.nlm.nih.gov/sites/entrez?db=pubmed&cmd=link&linkname=pubmed_pubmed&uid=17564346&ordinalpos=100:)

# [Myotonic dystrophy in a female with myasthenia gravis.](http://www.ncbi.nlm.nih.gov/pubmed/17560508?ordinalpos=101&itool=EntrezSystem2.PEntrez.Pubmed.Pubmed_ResultsPanel.Pubmed_DefaultReportPanel.Pubmed_RVDocSum)

de los Angeles Avaria M, Kleinsteuber K, Novoa F, Faundez P, Carvallo P.

Pediatr Neurol. 2007 Jun;36(6):421-3.

PMID: 17560508 [PubMed - indexed for MEDLINE]

# [Autoimmune disease in families with multiple sclerosis: a population-based study.](http://www.ncbi.nlm.nih.gov/pubmed/17560172?ordinalpos=102&itool=EntrezSystem2.PEntrez.Pubmed.Pubmed_ResultsPanel.Pubmed_DefaultReportPanel.Pubmed_RVDocSum)

Ramagopalan SV, Dyment DA, Valdar W, Herrera BM, Criscuoli M, Yee IM, Sadovnick AD, Ebers GC; Canadian Collaborative Study Group.

Lancet Neurol. 2007 Jul;6(7):604-10.

PMID: 17560172 [PubMed - indexed for MEDLINE]

# [Treatment of refractory chronic GVHD with rituximab: a GITMO study.](http://www.ncbi.nlm.nih.gov/pubmed/17549053?ordinalpos=103&itool=EntrezSystem2.PEntrez.Pubmed.Pubmed_ResultsPanel.Pubmed_DefaultReportPanel.Pubmed_RVDocSum)

Zaja F, Bacigalupo A, Patriarca F, Stanzani M, Van Lint MT, Filì C, Scimè R, Milone G, Falda M, Vener C, Laszlo D, Alessandrino PE, Narni F, Sica S, Olivieri A, Sperotto A, Bosi A, Bonifazi F, Fanin R; GITMO (Gruppo Italiano Trapianto Midollo Osseo).

Bone Marrow Transplant. 2007 Aug;40(3):273-7. Epub 2007 Jun 4.

PMID: 17549053 [PubMed - indexed for MEDLINE]

# [Current management options in myasthenia gravis.](http://www.ncbi.nlm.nih.gov/pubmed/17547852?ordinalpos=104&itool=EntrezSystem2.PEntrez.Pubmed.Pubmed_ResultsPanel.Pubmed_DefaultReportPanel.Pubmed_RVDocSum)

Reddel S.

Curr Allergy Asthma Rep. 2007 Jul;7(4):293-300. Review.

PMID: 17547852 [PubMed - indexed for MEDLINE]

# [Therapeutic options in autoimmune myasthenia gravis.](http://www.ncbi.nlm.nih.gov/pubmed/17537383?ordinalpos=105&itool=EntrezSystem2.PEntrez.Pubmed.Pubmed_ResultsPanel.Pubmed_DefaultReportPanel.Pubmed_RVDocSum)

García-Carrasco M, Escárcega RO, Fuentes-Alexandro S, Riebeling C, Cervera R.

Autoimmun Rev. 2007 Jun;6(6):373-8. Epub 2007 Jan 30. Review.

PMID: 17537383 [PubMed - indexed for MEDLINE]

[Related Articles](http://www.ncbi.nlm.nih.gov/sites/entrez?db=pubmed&cmd=link&linkname=pubmed_pubmed&uid=17537383&ordinalpos=105:)

# [The frequency of ectopic thymic tissue in the necks of patients without any thymic disease.](http://www.ncbi.nlm.nih.gov/pubmed/17534235?ordinalpos=106&itool=EntrezSystem2.PEntrez.Pubmed.Pubmed_ResultsPanel.Pubmed_DefaultReportPanel.Pubmed_RVDocSum)

Tabatabaie SA, Hashemi SM, Sanei B, Sanei MH.

Med Sci Monit. 2007 Jun;13(6):CR283-85.

PMID: 17534235 [PubMed - indexed for MEDLINE]

[Related Articles](http://www.ncbi.nlm.nih.gov/sites/entrez?db=pubmed&cmd=link&linkname=pubmed_pubmed&uid=17534235&ordinalpos=106:)

# [Mood and anxiety disorders in patients with myasthenia gravis: aetiology, diagnosis and treatment.](http://www.ncbi.nlm.nih.gov/pubmed/17521227?ordinalpos=107&itool=EntrezSystem2.PEntrez.Pubmed.Pubmed_ResultsPanel.Pubmed_DefaultReportPanel.Pubmed_RVDocSum)

Kulaksizoglu IB.

CNS Drugs. 2007;21(6):473-81. Review.

PMID: 17521227 [PubMed - indexed for MEDLINE]

# [Fruit, vegetable, vitamin A intakes, and prostate cancer risk.](http://www.ncbi.nlm.nih.gov/pubmed/17519926?ordinalpos=108&itool=EntrezSystem2.PEntrez.Pubmed.Pubmed_ResultsPanel.Pubmed_DefaultReportPanel.Pubmed_RVDocSum)

Ambrosini GL, de Klerk NH, Fritschi L, Mackerras D, Musk B.

Prostate Cancer Prostatic Dis. 2008;11(1):61-6. Epub 2007 May 22.

PMID: 17519926 [PubMed - indexed for MEDLINE]

[Related Articles](http://www.ncbi.nlm.nih.gov/sites/entrez?db=pubmed&cmd=link&linkname=pubmed_pubmed&uid=17519926&ordinalpos=108:)

# [[Clinical-biological analysis of thrombotic thrombocytopenic purpura in the General University Hospital of Alicante during 1983-2006]](http://www.ncbi.nlm.nih.gov/pubmed/17504669?ordinalpos=110&itool=EntrezSystem2.PEntrez.Pubmed.Pubmed_ResultsPanel.Pubmed_DefaultReportPanel.Pubmed_RVDocSum)

Verdú J, Fernández P, Rivas C, Romero A, Soler S, Verdú JJ.

Rev Clin Esp. 2007 May;207(5):243-5. Spanish.

PMID: 17504669 [PubMed - indexed for MEDLINE]

[Related Articles](http://www.ncbi.nlm.nih.gov/sites/entrez?db=pubmed&cmd=link&linkname=pubmed_pubmed&uid=17504669&ordinalpos=110:)

# [[Myasthenia gravis and pregnancy--a case report and review of the literature]](http://www.ncbi.nlm.nih.gov/pubmed/17489171?ordinalpos=111&itool=EntrezSystem2.PEntrez.Pubmed.Pubmed_ResultsPanel.Pubmed_DefaultReportPanel.Pubmed_RVDocSum)

Batashki I, Markova D, Milchev N, Terzhumanov R, Uchikova E, Uchikov A, Kuzmanov B, Iozgiur N.

Akush Ginekol (Sofiia). 2006;45(7):59-61. Bulgarian.

PMID: 17489171 [PubMed - indexed for MEDLINE]

# [[Autoimmunological and allergic disorders with Hashimoto and Graves disease]](http://www.ncbi.nlm.nih.gov/pubmed/17479856?ordinalpos=112&itool=EntrezSystem2.PEntrez.Pubmed.Pubmed_ResultsPanel.Pubmed_DefaultReportPanel.Pubmed_RVDocSum)

Przybylik-Mazurek E, Kotlinowska B, Kasztelnik M, Stefańska A, Huszno B.

Przegl Lek. 2006;63(9):719-22. Polish.

PMID: 17479856 [PubMed - indexed for MEDLINE]

[Related Articles](http://www.ncbi.nlm.nih.gov/sites/entrez?db=pubmed&cmd=link&linkname=pubmed_pubmed&uid=17479856&ordinalpos=112:)

# [Pemphigus vulgaris: recent advances in our understanding of its pathogenesis.](http://www.ncbi.nlm.nih.gov/pubmed/17452959?ordinalpos=113&itool=EntrezSystem2.PEntrez.Pubmed.Pubmed_ResultsPanel.Pubmed_DefaultReportPanel.Pubmed_RVDocSum)

Femiano F.

Minerva Stomatol. 2007 Apr;56(4):215-23. Review. English, Italian.

PMID: 17452959 [PubMed - indexed for MEDLINE]

[Related Articles](http://www.ncbi.nlm.nih.gov/sites/entrez?db=pubmed&cmd=link&linkname=pubmed_pubmed&uid=17452959&ordinalpos=113:)

# [Efficacy of various intravenous immunoglobulin therapy protocols in autoimmune and chronic inflammatory disorders.](http://www.ncbi.nlm.nih.gov/pubmed/17440006?ordinalpos=114&itool=EntrezSystem2.PEntrez.Pubmed.Pubmed_ResultsPanel.Pubmed_DefaultReportPanel.Pubmed_RVDocSum)

Gürcan HM, Ahmed AR.

Ann Pharmacother. 2007 May;41(5):812-23. Epub 2007 Apr 17. Review.

PMID: 17440006 [PubMed - indexed for MEDLINE]

# [Microscopic polyangiitis and myasthenia gravis: the battle of Occam and Hickam.](http://www.ncbi.nlm.nih.gov/pubmed/17415507?ordinalpos=115&itool=EntrezSystem2.PEntrez.Pubmed.Pubmed_ResultsPanel.Pubmed_DefaultReportPanel.Pubmed_RVDocSum)

Holmes MV, Sen D.

Clin Rheumatol. 2007 Nov;26(11):1981-3. Epub 2007 Apr 6.

PMID: 17415507 [PubMed - indexed for MEDLINE]

[Related Articles](http://www.ncbi.nlm.nih.gov/sites/entrez?db=pubmed&cmd=link&linkname=pubmed_pubmed&uid=17415507&ordinalpos=115:)

# [Association between thyroid autoimmune dysfunction and non-thyroid autoimmune diseases.](http://www.ncbi.nlm.nih.gov/pubmed/17413622?ordinalpos=116&itool=EntrezSystem2.PEntrez.Pubmed.Pubmed_ResultsPanel.Pubmed_DefaultReportPanel.Pubmed_RVDocSum)

Cruz AA, Akaishi PM, Vargas MA, de Paula SA.

Ophthal Plast Reconstr Surg. 2007 Mar-Apr;23(2):104-8.

PMID: 17413622 [PubMed - indexed for MEDLINE]

[Related Articles](http://www.ncbi.nlm.nih.gov/sites/entrez?db=pubmed&cmd=link&linkname=pubmed_pubmed&uid=17413622&ordinalpos=116:)

# [[Neuromuscular complications in transplant recipients]](http://www.ncbi.nlm.nih.gov/pubmed/17404516?ordinalpos=117&itool=EntrezSystem2.PEntrez.Pubmed.Pubmed_ResultsPanel.Pubmed_DefaultReportPanel.Pubmed_RVDocSum)

Echaniz-Laguna A.

Rev Neurol (Paris). 2007 Mar;163(3):275-85. Review. French.

PMID: 17404516 [PubMed - indexed for MEDLINE]

[Related Articles](http://www.ncbi.nlm.nih.gov/sites/entrez?db=pubmed&cmd=link&linkname=pubmed_pubmed&uid=17404516&ordinalpos=117:)

# [IV immunoglobulin in patients with myasthenia gravis: a randomized controlled trial.](http://www.ncbi.nlm.nih.gov/pubmed/17353471?ordinalpos=118&itool=EntrezSystem2.PEntrez.Pubmed.Pubmed_ResultsPanel.Pubmed_DefaultReportPanel.Pubmed_RVDocSum)

Zinman L, Ng E, Bril V.

Neurology. 2007 Mar 13;68(11):837-41.

PMID: 17353471 [PubMed - indexed for MEDLINE]

[Related Articles](http://www.ncbi.nlm.nih.gov/sites/entrez?db=pubmed&cmd=link&linkname=pubmed_pubmed&uid=17353471&ordinalpos=118:)

# [IVIG in myasthenia gravis: getting enough "bang for the buck".](http://www.ncbi.nlm.nih.gov/pubmed/17353466?ordinalpos=119&itool=EntrezSystem2.PEntrez.Pubmed.Pubmed_ResultsPanel.Pubmed_DefaultReportPanel.Pubmed_RVDocSum)

Meriggioli MN.

Neurology. 2007 Mar 13;68(11):803-4. No abstract available.

PMID: 17353466 [PubMed - indexed for MEDLINE]

[Related Articles](http://www.ncbi.nlm.nih.gov/sites/entrez?db=pubmed&cmd=link&linkname=pubmed_pubmed&uid=17353466&ordinalpos=119:)

# [Myasthenia gravis: a long term follow-up study of Swedish patients with specific reference to thymic histology.](http://www.ncbi.nlm.nih.gov/pubmed/17353257?ordinalpos=120&itool=EntrezSystem2.PEntrez.Pubmed.Pubmed_ResultsPanel.Pubmed_DefaultReportPanel.Pubmed_RVDocSum)

Tsinzerling N, Lefvert AK, Matell G, Pirskanen-Matell R.

J Neurol Neurosurg Psychiatry. 2007 Oct;78(10):1109-12. Epub 2007 Mar 12.

PMID: 17353257 [PubMed - indexed for MEDLINE]

# [[Age at the onset of intractable disease: based on a clinical database for patients receiving financial aid for treatment]](http://www.ncbi.nlm.nih.gov/pubmed/17338189?ordinalpos=121&itool=EntrezSystem2.PEntrez.Pubmed.Pubmed_ResultsPanel.Pubmed_DefaultReportPanel.Pubmed_RVDocSum)

Ohta A, Nagai M, Nishina M, Shibazaki S, Ishijima H, Izumida M.

Nippon Koshu Eisei Zasshi. 2007 Jan;54(1):3-14. Japanese.

PMID: 17338189 [PubMed - indexed for MEDLINE]

[Related Articles](http://www.ncbi.nlm.nih.gov/sites/entrez?db=pubmed&cmd=link&linkname=pubmed_pubmed&uid=17338189&ordinalpos=121:)

**122:**

# [Paraneoplastic diseases associated with thymoma.](http://www.ncbi.nlm.nih.gov/pubmed/17325820?ordinalpos=122&itool=EntrezSystem2.PEntrez.Pubmed.Pubmed_ResultsPanel.Pubmed_DefaultReportPanel.Pubmed_RVDocSum)

Evoli A, Minicuci GM, Vitaliani R, Battaglia A, Della Marca G, Lauriola L, Fattorossi A.

J Neurol. 2007 Jun;254(6):756-62. Epub 2007 Feb 26.

PMID: 17325820 [PubMed - indexed for MEDLINE]

# [Thoracoscopic direct suture repair of thoracic duct injury after thoracoscopic mediastinal surgery.](http://www.ncbi.nlm.nih.gov/pubmed/17318061?ordinalpos=123&itool=EntrezSystem2.PEntrez.Pubmed.Pubmed_ResultsPanel.Pubmed_DefaultReportPanel.Pubmed_RVDocSum)

Cho DG, Cho KD, Jo MS.

Surg Laparosc Endosc Percutan Tech. 2007 Feb;17(1):60-1.

PMID: 17318061 [PubMed - indexed for MEDLINE]

# [Bilateral ocular paralysis: analysis of 31 inpatients.](http://www.ncbi.nlm.nih.gov/pubmed/17296832?ordinalpos=125&itool=EntrezSystem2.PEntrez.Pubmed.Pubmed_ResultsPanel.Pubmed_DefaultReportPanel.Pubmed_RVDocSum)

Keane JR.

Arch Neurol. 2007 Feb;64(2):178-80.

PMID: 17296832 [PubMed - indexed for MEDLINE]

# [Age and sex differences in malignant mesothelioma after residential exposure to blue asbestos (crocidolite).](http://www.ncbi.nlm.nih.gov/pubmed/17296636?ordinalpos=126&itool=EntrezSystem2.PEntrez.Pubmed.Pubmed_ResultsPanel.Pubmed_DefaultReportPanel.Pubmed_RVDocSum)

Reid A, Berry G, de Klerk N, Hansen J, Heyworth J, Ambrosini G, Fritschi L, Olsen N, Merler E, Musk AW.

Chest. 2007 Feb;131(2):376-82.

PMID: 17296636 [PubMed - indexed for MEDLINE]

# [Immune-related disease before and after vasectomy: an epidemiological database study.](http://www.ncbi.nlm.nih.gov/pubmed/17284511?ordinalpos=127&itool=EntrezSystem2.PEntrez.Pubmed.Pubmed_ResultsPanel.Pubmed_DefaultReportPanel.Pubmed_RVDocSum)

Goldacre MJ, Wotton CJ, Seagroatt V, Yeates D.

Hum Reprod. 2007 May;22(5):1273-8. Epub 2007 Feb 6.

PMID: 17284511 [PubMed - indexed for MEDLINE]

# [The distribution of parenchyma, follicles, and lymphocyte subsets in thymus of patients with myasthenia gravis, with special reference to remission after thymectomy.](http://www.ncbi.nlm.nih.gov/pubmed/17258565?ordinalpos=128&itool=EntrezSystem2.PEntrez.Pubmed.Pubmed_ResultsPanel.Pubmed_DefaultReportPanel.Pubmed_RVDocSum)

Mori T, Nomori H, Ikeda K, Kobayashi H, Iwatani K, Kobayashi T.

J Thorac Cardiovasc Surg. 2007 Feb;133(2):364-8.

PMID: 17258565 [PubMed - indexed for MEDLINE]

[Related Articles](http://www.ncbi.nlm.nih.gov/sites/entrez?db=pubmed&cmd=link&linkname=pubmed_pubmed&uid=17258565&ordinalpos=128:)

# [Risk of malignancy in myasthenia gravis patients exposed to azathioprine therapy for a median period of 3 years.](http://www.ncbi.nlm.nih.gov/pubmed/17252153?ordinalpos=130&itool=EntrezSystem2.PEntrez.Pubmed.Pubmed_ResultsPanel.Pubmed_DefaultReportPanel.Pubmed_RVDocSum)

Rawoot A, Little F, Heckmann JM.

S Afr Med J. 2006 Dec;96(12):1249-51. No abstract available.

PMID: 17252153 [PubMed - indexed for MEDLINE]

[Related Articles](http://www.ncbi.nlm.nih.gov/sites/entrez?db=pubmed&cmd=link&linkname=pubmed_pubmed&uid=17252153&ordinalpos=130:)

# [Therapeutic plasma exchange in the treatment of neuroimmunologic disorders: review of 50 cases.](http://www.ncbi.nlm.nih.gov/pubmed/17224307?ordinalpos=131&itool=EntrezSystem2.PEntrez.Pubmed.Pubmed_ResultsPanel.Pubmed_DefaultReportPanel.Pubmed_RVDocSum)

Yücesan C, Arslan O, Arat M, Yücemen N, Ayyildiz E, Ilhan O, Mutluer N.

Transfus Apher Sci. 2007 Feb;36(1):103-7. Epub 2007 Jan 16. Review.

PMID: 17224307 [PubMed - indexed for MEDLINE]

# [Myasthenia gravis in pregnancy and birth: identifying risk factors, optimising care.](http://www.ncbi.nlm.nih.gov/pubmed/17222111?ordinalpos=132&itool=EntrezSystem2.PEntrez.Pubmed.Pubmed_ResultsPanel.Pubmed_DefaultReportPanel.Pubmed_RVDocSum)

Hoff JM, Daltveit AK, Gilhus NE.

Eur J Neurol. 2007 Jan;14(1):38-43.

PMID: 17222111 [PubMed - indexed for MEDLINE]

# [[Myasthenia gravis and intestinal resection: is dehiscence likely to occur?]](http://www.ncbi.nlm.nih.gov/pubmed/17211359?ordinalpos=133&itool=EntrezSystem2.PEntrez.Pubmed.Pubmed_ResultsPanel.Pubmed_DefaultReportPanel.Pubmed_RVDocSum)

Li Destri G, Scilletta B, Latino R, Di Cataldo A.

Minerva Chir. 2006 Dec;61(6):525-8. Italian.

PMID: 17211359 [PubMed - indexed for MEDLINE]

# [Etiological pattern of blepharoptosis among patients presenting in teaching hospital.](http://www.ncbi.nlm.nih.gov/pubmed/17189964?ordinalpos=134&itool=EntrezSystem2.PEntrez.Pubmed.Pubmed_ResultsPanel.Pubmed_DefaultReportPanel.Pubmed_RVDocSum)

Thapa R, Karmacharya PC, Nepal BP.

JNMA J Nepal Med Assoc. 2006 Apr-Jun;45(162):218-22.

PMID: 17189964 [PubMed - indexed for MEDLINE]

# [The CHRNE 470del20 mutation causing congenital myasthenic syndrome in South African Brahman cattle: prevalence, origin, and association with performance traits.](http://www.ncbi.nlm.nih.gov/pubmed/17121978?ordinalpos=135&itool=EntrezSystem2.PEntrez.Pubmed.Pubmed_ResultsPanel.Pubmed_DefaultReportPanel.Pubmed_RVDocSum)

Thompson PN, van der Werf JH, Heesterbeek JA, van Arendonk JA.

J Anim Sci. 2007 Mar;85(3):604-9. Epub 2006 Nov 22.

PMID: 17121978 [PubMed - indexed for MEDLINE]

# [[The differential diagnostic criteria and clinical features of endocrine ophtalmopathy in patients suffering from myastenia with oculomotor disturbances]](http://www.ncbi.nlm.nih.gov/pubmed/17087196?ordinalpos=137&itool=EntrezSystem2.PEntrez.Pubmed.Pubmed_ResultsPanel.Pubmed_DefaultReportPanel.Pubmed_RVDocSum)

Zakutniaia VN, Ametov AS, Gekht BM, Sidnev DV, Sanadze AG, Ivanova AN.

Klin Med (Mosk). 2006;84(8):62-6. Russian.

PMID: 17087196 [PubMed - indexed for MEDLINE]

# [Successful treatment of disseminated scedosporium infection in 2 lung transplant recipients: review of the literature and recommendations for management.](http://www.ncbi.nlm.nih.gov/pubmed/17045941?ordinalpos=139&itool=EntrezSystem2.PEntrez.Pubmed.Pubmed_ResultsPanel.Pubmed_DefaultReportPanel.Pubmed_RVDocSum)

Musk M, Chambers D, Chin W, Murray R, Gabbay E.

J Heart Lung Transplant. 2006 Oct;25(10):1268-72. Review.

PMID: 17045941 [PubMed - indexed for MEDLINE]

[Related Articles](http://www.ncbi.nlm.nih.gov/sites/entrez?db=pubmed&cmd=link&linkname=pubmed_pubmed&uid=17045941&ordinalpos=139:)

# [Occurrence of CNS demyelinating disease in patients with myasthenia gravis.](http://www.ncbi.nlm.nih.gov/pubmed/16966558?ordinalpos=142&itool=EntrezSystem2.PEntrez.Pubmed.Pubmed_ResultsPanel.Pubmed_DefaultReportPanel.Pubmed_RVDocSum)

Gotkine M, Fellig Y, Abramsky O.

Neurology. 2006 Sep 12;67(5):881-3.

PMID: 16966558 [PubMed - indexed for MEDLINE]

# [Associations of a novel IL4RA polymorphism, Ala57Thr, in Greenlander Inuit.](http://www.ncbi.nlm.nih.gov/pubmed/16950281?ordinalpos=143&itool=EntrezSystem2.PEntrez.Pubmed.Pubmed_ResultsPanel.Pubmed_DefaultReportPanel.Pubmed_RVDocSum)

Khoo SK, Zhang G, Backer V, Porsbjerg C, Nepper-Christensen S, Creegan R, Baynam G, de Klerk N, Rossi GA, Hagel I, Di Prisco MC, Lynch N, Britton J, Hall I, Musk AW, Goldblatt J, Le Souëf PN, Greenlandic Population Study Group.

J Allergy Clin Immunol. 2006 Sep;118(3):627-34. Epub 2006 Jun 27.

PMID: 16950281 [PubMed - indexed for MEDLINE]

# [Toxicity of synthetic musks to early life stages of the freshwater mussel Lampsilis cardium.](http://www.ncbi.nlm.nih.gov/pubmed/16944041?ordinalpos=144&itool=EntrezSystem2.PEntrez.Pubmed.Pubmed_ResultsPanel.Pubmed_DefaultReportPanel.Pubmed_RVDocSum)

Gooding MP, Newton TJ, Bartsch MR, Hornbuckle KC.

Arch Environ Contam Toxicol. 2006 Nov;51(4):549-58. Epub 2006 Aug 30.

PMID: 16944041 [PubMed - indexed for MEDLINE]

# [SSR180711, a novel selective alpha7 nicotinic receptor partial agonist: (II) efficacy in experimental models predictive of activity against cognitive symptoms of schizophrenia.](http://www.ncbi.nlm.nih.gov/pubmed/16936709?ordinalpos=145&itool=EntrezSystem2.PEntrez.Pubmed.Pubmed_ResultsPanel.Pubmed_DefaultReportPanel.Pubmed_RVDocSum)

Pichat P, Bergis OE, Terranova JP, Urani A, Duarte C, Santucci V, Gueudet C, Voltz C, Steinberg R, Stemmelin J, Oury-Donat F, Avenet P, Griebel G, Scatton B.

Neuropsychopharmacology. 2007 Jan;32(1):17-34. Epub 2006 Aug 23.

PMID: 16936709 [PubMed - indexed for MEDLINE]

# [Neuromuscular disease causing acute respiratory failure.](http://www.ncbi.nlm.nih.gov/pubmed/16934165?ordinalpos=146&itool=EntrezSystem2.PEntrez.Pubmed.Pubmed_ResultsPanel.Pubmed_DefaultReportPanel.Pubmed_RVDocSum)

Mehta S.

Respir Care. 2006 Sep;51(9):1016-21; discussion 1021-3. Review.

PMID: 16934165 [PubMed - indexed for MEDLINE]

# [Impaired receptor clustering in congenital myasthenic syndrome with novel RAPSN mutations.](http://www.ncbi.nlm.nih.gov/pubmed/16931511?ordinalpos=147&itool=EntrezSystem2.PEntrez.Pubmed.Pubmed_ResultsPanel.Pubmed_DefaultReportPanel.Pubmed_RVDocSum)

Müller JS, Baumeister SK, Rasic VM, Krause S, Todorovic S, Kugler K, Müller-Felber W, Abicht A, Lochmüller H.

Neurology. 2006 Oct 10;67(7):1159-64. Epub 2006 Aug 23.

PMID: 16931511 [PubMed - indexed for MEDLINE]

# [Safety of long-term combined immunosuppressive treatment in myasthenia gravis--analysis of adverse effects of 163 patients.](http://www.ncbi.nlm.nih.gov/pubmed/16930359?ordinalpos=148&itool=EntrezSystem2.PEntrez.Pubmed.Pubmed_ResultsPanel.Pubmed_DefaultReportPanel.Pubmed_RVDocSum)

Rozsa C, Lovas G, Fornadi L, Szabo G, Komoly S.

Eur J Neurol. 2006 Sep;13(9):947-52.

PMID: 16930359 [PubMed - indexed for MEDLINE]

# [Thoracoscopic thymectomy mid-term results.](http://www.ncbi.nlm.nih.gov/pubmed/16928524?ordinalpos=149&itool=EntrezSystem2.PEntrez.Pubmed.Pubmed_ResultsPanel.Pubmed_DefaultReportPanel.Pubmed_RVDocSum)

Tomulescu V, Ion V, Kosa A, Sgarbura O, Popescu I.

Ann Thorac Surg. 2006 Sep;82(3):1003-7.

PMID: 16928524 [PubMed - indexed for MEDLINE]

# [[A case of myasthenia gravis appearing after thymomectomy: with histopathological investigation]](http://www.ncbi.nlm.nih.gov/pubmed/16910471?ordinalpos=150&itool=EntrezSystem2.PEntrez.Pubmed.Pubmed_ResultsPanel.Pubmed_DefaultReportPanel.Pubmed_RVDocSum)

Sunayama M, Miyamoto K, Shioyama M, Sato T, Kusunoki S.

No To Shinkei. 2006 Jul;58(7):615-9. Japanese.

PMID: 16910471 [PubMed - indexed for MEDLINE]

# [Plasma exchange in neuroimmunological disorders: part 2. Treatment of neuromuscular disorders.](http://www.ncbi.nlm.nih.gov/pubmed/16908731?ordinalpos=151&itool=EntrezSystem2.PEntrez.Pubmed.Pubmed_ResultsPanel.Pubmed_DefaultReportPanel.Pubmed_RVDocSum)

Lehmann HC, Hartung HP, Hetzel GR, Stüve O, Kieseier BC.

Arch Neurol. 2006 Aug;63(8):1066-71. Review.

PMID: 16908731 [PubMed - indexed for MEDLINE]

# [Familial risks for diseases of myoneural junction and muscle in siblings based on hospitalizations and deaths in sweden.](http://www.ncbi.nlm.nih.gov/pubmed/16899165?ordinalpos=152&itool=EntrezSystem2.PEntrez.Pubmed.Pubmed_ResultsPanel.Pubmed_DefaultReportPanel.Pubmed_RVDocSum)

Hemminki K, Li X, Sundquist K.

Twin Res Hum Genet. 2006 Aug;9(4):573-9.

PMID: 16899165 [PubMed - indexed for MEDLINE]

# [Acetylcholine receptor antibodies in myasthenia gravis are associated with greater risk of diabetes and thyroid disease.](http://www.ncbi.nlm.nih.gov/pubmed/16867036?ordinalpos=153&itool=EntrezSystem2.PEntrez.Pubmed.Pubmed_ResultsPanel.Pubmed_DefaultReportPanel.Pubmed_RVDocSum)

Toth C, McDonald D, Oger J, Brownell K.

Acta Neurol Scand. 2006 Aug;114(2):124-32.

PMID: 16867036 [PubMed - indexed for MEDLINE]

# [Myasthenia gravis in children: a longitudinal study.](http://www.ncbi.nlm.nih.gov/pubmed/16867035?ordinalpos=154&itool=EntrezSystem2.PEntrez.Pubmed.Pubmed_ResultsPanel.Pubmed_DefaultReportPanel.Pubmed_RVDocSum)

Ashraf VV, Taly AB, Veerendrakumar M, Rao S.

Acta Neurol Scand. 2006 Aug;114(2):119-23.

PMID: 16867035 [PubMed - indexed for MEDLINE]

[Related Articles](http://www.ncbi.nlm.nih.gov/sites/entrez?db=pubmed&cmd=link&linkname=pubmed_pubmed&uid=16867035&ordinalpos=154:)

# [The risk of lung cancer with increasing time since ceasing exposure to asbestos and quitting smoking.](http://www.ncbi.nlm.nih.gov/pubmed/16849527?ordinalpos=155&itool=EntrezSystem2.PEntrez.Pubmed.Pubmed_ResultsPanel.Pubmed_DefaultReportPanel.Pubmed_RVDocSum)

Reid A, de Klerk NH, Ambrosini GL, Berry G, Musk AW.

Occup Environ Med. 2006 Aug;63(8):509-12.

PMID: 16849527 [PubMed - indexed for MEDLINE]

# [Plasma vitamin concentrations and incidence of mesothelioma and lung cancer in individuals exposed to crocidolite at Wittenoom, Western Australia.](http://www.ncbi.nlm.nih.gov/pubmed/16835500?ordinalpos=156&itool=EntrezSystem2.PEntrez.Pubmed.Pubmed_ResultsPanel.Pubmed_DefaultReportPanel.Pubmed_RVDocSum)

Alfonso HS, Fritschi L, de Klerk NH, Ambrosini GL, Beilby J, Olsen N, Musk AW.

Eur J Cancer Prev. 2006 Aug;15(4):290-4.

PMID: 16835500 [PubMed - indexed for MEDLINE]

# [Sleep apnea in patients with myasthenia gravis.](http://www.ncbi.nlm.nih.gov/pubmed/16832094?ordinalpos=157&itool=EntrezSystem2.PEntrez.Pubmed.Pubmed_ResultsPanel.Pubmed_DefaultReportPanel.Pubmed_RVDocSum)

Nicolle MW, Rask S, Koopman WJ, George CF, Adams J, Wiebe S.

Neurology. 2006 Jul 11;67(1):140-2.

PMID: 16832094 [PubMed - indexed for MEDLINE]

# [Clinical outcomes following extended thymectomy for myasthenia gravis: report of 17 cases.](http://www.ncbi.nlm.nih.gov/pubmed/16823336?ordinalpos=158&itool=EntrezSystem2.PEntrez.Pubmed.Pubmed_ResultsPanel.Pubmed_DefaultReportPanel.Pubmed_RVDocSum)

Hase R, Sugiura H, Fukunaga A, Takahashi H, Simozawa E, Kondo S.

Ann Thorac Cardiovasc Surg. 2006 Jun;12(3):203-6.

PMID: 16823336 [PubMed - indexed for MEDLINE]

[Related Articles](http://www.ncbi.nlm.nih.gov/sites/entrez?db=pubmed&cmd=link&linkname=pubmed_pubmed&uid=16823336&ordinalpos=158:)

# [Is myasthenia gravis more benign in the Indian population?](http://www.ncbi.nlm.nih.gov/pubmed/16804279?ordinalpos=159&itool=EntrezSystem2.PEntrez.Pubmed.Pubmed_ResultsPanel.Pubmed_DefaultReportPanel.Pubmed_RVDocSum)

Menon A, Madhukar M, Sreedhar A.

Neurol India. 2006 Jun;54(2):217-8. No abstract available.

PMID: 16804279 [PubMed - indexed for MEDLINE]

# [Anti-interferon autoantibodies in autoimmune polyendocrinopathy syndrome type 1.](http://www.ncbi.nlm.nih.gov/pubmed/16784312?ordinalpos=160&itool=EntrezSystem2.PEntrez.Pubmed.Pubmed_ResultsPanel.Pubmed_DefaultReportPanel.Pubmed_RVDocSum)

Meager A, Visvalingam K, Peterson P, Möll K, Murumägi A, Krohn K, Eskelin P, Perheentupa J, Husebye E, Kadota Y, Willcox N.

PLoS Med. 2006 Jul;3(7):e289.

PMID: 16784312 [PubMed - indexed for MEDLINE]

[Related Articles](http://www.ncbi.nlm.nih.gov/sites/entrez?db=pubmed&cmd=link&linkname=pubmed_pubmed&uid=16784312&ordinalpos=160:) [Free article in PMC](http://www.ncbi.nlm.nih.gov/pubmed/16784312?ordinalpos=160&itool=EntrezSystem2.PEntrez.Pubmed.Pubmed_ResultsPanel.Pubmed_DefaultReportPanel.Pubmed_RVDocSum&log$=freepmc)

# [[Video-assisted, extended thymectomy for the treatment of myasthenia gravis. Our early experiences]](http://www.ncbi.nlm.nih.gov/pubmed/16784034?ordinalpos=161&itool=EntrezSystem2.PEntrez.Pubmed.Pubmed_ResultsPanel.Pubmed_DefaultReportPanel.Pubmed_RVDocSum)

Furák J, Troján I, Szoke T, Lázár G.

Magy Seb. 2006 Apr;59(2):112-6. Hungarian.

PMID: 16784034 [PubMed - indexed for MEDLINE]

# [Strong association of MuSK antibody-positive myasthenia gravis and HLA-DR14-DQ5.](http://www.ncbi.nlm.nih.gov/pubmed/16769963?ordinalpos=162&itool=EntrezSystem2.PEntrez.Pubmed.Pubmed_ResultsPanel.Pubmed_DefaultReportPanel.Pubmed_RVDocSum)

Niks EH, Kuks JB, Roep BO, Haasnoot GW, Verduijn W, Ballieux BE, De Baets MH, Vincent A, Verschuuren JJ.

Neurology. 2006 Jun 13;66(11):1772-4.

PMID: 16769963 [PubMed - indexed for MEDLINE]

# [Neuromyelitis optica in patients with myasthenia gravis who underwent thymectomy.](http://www.ncbi.nlm.nih.gov/pubmed/16769866?ordinalpos=163&itool=EntrezSystem2.PEntrez.Pubmed.Pubmed_ResultsPanel.Pubmed_DefaultReportPanel.Pubmed_RVDocSum)

Kister I, Gulati S, Boz C, Bergamaschi R, Piccolo G, Oger J, Swerdlow ML.

Arch Neurol. 2006 Jun;63(6):851-6. Erratum in: Arch Neurol. 2006 Sep;63(9):1342. Piccolo, Guiseppe [corrected to Piccolo, Giovanni].

PMID: 16769866 [PubMed - indexed for MEDLINE]

# [Anti-interferon auto-antibodies in autoimmune polyendocrinopathy syndrome type 1.](http://www.ncbi.nlm.nih.gov/pubmed/16756392?ordinalpos=165&itool=EntrezSystem2.PEntrez.Pubmed.Pubmed_ResultsPanel.Pubmed_DefaultReportPanel.Pubmed_RVDocSum)

Levin M.

PLoS Med. 2006 Jul;3(7):e292. No abstract available.

PMID: 16756392 [PubMed - indexed for MEDLINE]

# [[Is the microscopic morphology of thymoma of clinical significance?]](http://www.ncbi.nlm.nih.gov/pubmed/16756145?ordinalpos=166&itool=EntrezSystem2.PEntrez.Pubmed.Pubmed_ResultsPanel.Pubmed_DefaultReportPanel.Pubmed_RVDocSum)

Szołkowska M, Langfort R, Roszkowski-Sliz K.

Pneumonol Alergol Pol. 2005;73(2):153-9. Polish.

PMID: 16756145 [PubMed - indexed for MEDLINE]

# [HLA-DQ polymorphism in Turkish patients with myasthenia gravis.](http://www.ncbi.nlm.nih.gov/pubmed/16720217?ordinalpos=167&itool=EntrezSystem2.PEntrez.Pubmed.Pubmed_ResultsPanel.Pubmed_DefaultReportPanel.Pubmed_RVDocSum)

Saruhan-Direskeneli G, Kiliç A, Parman Y, Serdaroğlu P, Deymeer F.

Hum Immunol. 2006 Apr-May;67(4-5):352-8. Epub 2006 Apr 7.

PMID: 16720217 [PubMed - indexed for MEDLINE]

# [[Oral EBV-associated diffuse large B-cell lymphomas in HIV-negative immunocompromised patients]](http://www.ncbi.nlm.nih.gov/pubmed/16685566?ordinalpos=168&itool=EntrezSystem2.PEntrez.Pubmed.Pubmed_ResultsPanel.Pubmed_DefaultReportPanel.Pubmed_RVDocSum)

Rhinow K, Schirmer I, Loddenkemper C, Anagnostopoulos I, Stein H, Reichart PA.

Mund Kiefer Gesichtschir. 2006 May;10(3):155-61. German.

PMID: 16685566 [PubMed - indexed for MEDLINE]

# [Vomeronasal sensory neurons from Sternotherus odoratus (stinkpot/musk turtle) respond to chemosignals via the phospholipase C system.](http://www.ncbi.nlm.nih.gov/pubmed/16651557?ordinalpos=169&itool=EntrezSystem2.PEntrez.Pubmed.Pubmed_ResultsPanel.Pubmed_DefaultReportPanel.Pubmed_RVDocSum)

Brann JH, Fadool DA.

J Exp Biol. 2006 May;209(Pt 10):1914-27.

PMID: 16651557 [PubMed - indexed for MEDLINE]

# [[Oral EBV-associated diffuse large B-cell lymphomas in HIV-negative immunocompromised patients.]](http://www.ncbi.nlm.nih.gov/pubmed/16639575?ordinalpos=170&itool=EntrezSystem2.PEntrez.Pubmed.Pubmed_ResultsPanel.Pubmed_DefaultReportPanel.Pubmed_RVDocSum)

Rhinow K, Schirmer I, Loddenkemper C, Anagnostopoulos I, Stein H, Reichart PA.

Mund Kiefer Gesichtschir. 2006 Apr 26. [Epub ahead of print] German.

PMID: 16639575 [PubMed - as supplied by publisher]

# [Does myasthenia gravis provide protection against cancer?](http://www.ncbi.nlm.nih.gov/pubmed/16637926?ordinalpos=171&itool=EntrezSystem2.PEntrez.Pubmed.Pubmed_ResultsPanel.Pubmed_DefaultReportPanel.Pubmed_RVDocSum)

Owe JF, Daltveit AK, Gilhus NE.

Acta Neurol Scand Suppl. 2006;183:33-6.

PMID: 16637926 [PubMed - indexed for MEDLINE]

# [Artrogryposis multiplex congenita -- a rare fetal condition caused by maternal myasthenia gravis.](http://www.ncbi.nlm.nih.gov/pubmed/16637924?ordinalpos=172&itool=EntrezSystem2.PEntrez.Pubmed.Pubmed_ResultsPanel.Pubmed_DefaultReportPanel.Pubmed_RVDocSum)

Hoff JM, Daltveit AK, Gilhus NE.

Acta Neurol Scand Suppl. 2006;183:26-7.

PMID: 16637924 [PubMed - indexed for MEDLINE]

# [[Myasthenia gravis and pregnancy: clinical course and management of delivery and the postpartum phase]](http://www.ncbi.nlm.nih.gov/pubmed/16585888?ordinalpos=173&itool=EntrezSystem2.PEntrez.Pubmed.Pubmed_ResultsPanel.Pubmed_DefaultReportPanel.Pubmed_RVDocSum)

Ramirez C, de Seze J, Delrieu O, Stojkovic T, Delalande S, Fourrier F, Leys D, Defebvre L, Destée A, Vermersch P.

Rev Neurol (Paris). 2006 Mar;162(3):330-8. French.

PMID: 16585888 [PubMed - indexed for MEDLINE]

# [[The neurology of pregnancy]](http://www.ncbi.nlm.nih.gov/pubmed/16585883?ordinalpos=174&itool=EntrezSystem2.PEntrez.Pubmed.Pubmed_ResultsPanel.Pubmed_DefaultReportPanel.Pubmed_RVDocSum)

Honnorat J.

Rev Neurol (Paris). 2006 Mar;162(3):293-4. French. No abstract available.

PMID: 16585883 [PubMed - indexed for MEDLINE]

[Related Articles](http://www.ncbi.nlm.nih.gov/sites/entrez?db=pubmed&cmd=link&linkname=pubmed_pubmed&uid=16585883&ordinalpos=174:)

# [Persistent increased risk for thymoma in myasthenia gravis associated with myositis.](http://www.ncbi.nlm.nih.gov/pubmed/16583371?ordinalpos=176&itool=EntrezSystem2.PEntrez.Pubmed.Pubmed_ResultsPanel.Pubmed_DefaultReportPanel.Pubmed_RVDocSum)

Hengstman GJ, Drost G, Wagenaar M, van Engelen BG.

Muscle Nerve. 2006 Aug;34(2):251-2. No abstract available.

PMID: 16583371 [PubMed - indexed for MEDLINE]

# [Phosphorylation of the nicotinic acetylcholine receptor in myotube-cholinergic neuron cocultures.](http://www.ncbi.nlm.nih.gov/pubmed/16555299?ordinalpos=177&itool=EntrezSystem2.PEntrez.Pubmed.Pubmed_ResultsPanel.Pubmed_DefaultReportPanel.Pubmed_RVDocSum)

Lanuza MA, Gizaw R, Viloria A, González CM, Besalduch N, Dunlap V, Tomàs J, Nelson PG.

J Neurosci Res. 2006 Jun;83(8):1407-14.

PMID: 16555299 [PubMed - indexed for MEDLINE]

# [Mediastinal tumors: a clinicopathological analysis.](http://www.ncbi.nlm.nih.gov/pubmed/16551814?ordinalpos=178&itool=EntrezSystem2.PEntrez.Pubmed.Pubmed_ResultsPanel.Pubmed_DefaultReportPanel.Pubmed_RVDocSum)

Shrivastava CP, Devgarha S, Ahlawat V.

Asian Cardiovasc Thorac Ann. 2006 Apr;14(2):102-4.

PMID: 16551814 [PubMed - indexed for MEDLINE]

[Related Articles](http://www.ncbi.nlm.nih.gov/sites/entrez?db=pubmed&cmd=link&linkname=pubmed_pubmed&uid=16551814&ordinalpos=178:)

# [Use of repetitive nerve stimulation in the evaluation of neuromuscular junction disorders.](http://www.ncbi.nlm.nih.gov/pubmed/16457051?ordinalpos=180&itool=EntrezSystem2.PEntrez.Pubmed.Pubmed_ResultsPanel.Pubmed_DefaultReportPanel.Pubmed_RVDocSum)

Zivković SA, Shipe C.

Am J Electroneurodiagnostic Technol. 2005 Dec;45(4):248-61. Review.

PMID: 16457051 [PubMed - indexed for MEDLINE]

# [New ruminant hosts and wider geographic range identified for Babesia odocoilei (Emerson and Wright 1970).](http://www.ncbi.nlm.nih.gov/pubmed/16456156?ordinalpos=181&itool=EntrezSystem2.PEntrez.Pubmed.Pubmed_ResultsPanel.Pubmed_DefaultReportPanel.Pubmed_RVDocSum)

Schoelkopf L, Hutchinson CE, Bendele KG, Goff WL, Willette M, Rasmussen JM, Holman PJ.

J Wildl Dis. 2005 Oct;41(4):683-90.

PMID: 16456156 [PubMed - indexed for MEDLINE]

# [[Muscular complications of human immunodeficiency virus (HIV) infection in the era of effective anti-retroviral therapy]](http://www.ncbi.nlm.nih.gov/pubmed/16446625?ordinalpos=182&itool=EntrezSystem2.PEntrez.Pubmed.Pubmed_ResultsPanel.Pubmed_DefaultReportPanel.Pubmed_RVDocSum)

Authier FJ, Gherardi RK.

Rev Neurol (Paris). 2006 Jan;162(1):71-81. Review. French.

PMID: 16446625 [PubMed - indexed for MEDLINE]

# [Experience with the "da Vinci" robotic system for thymectomy in patients with myasthenia gravis: report of 33 cases.](http://www.ncbi.nlm.nih.gov/pubmed/16427830?ordinalpos=183&itool=EntrezSystem2.PEntrez.Pubmed.Pubmed_ResultsPanel.Pubmed_DefaultReportPanel.Pubmed_RVDocSum)

Rea F, Marulli G, Bortolotti L, Feltracco P, Zuin A, Sartori F.

Ann Thorac Surg. 2006 Feb;81(2):455-9.

PMID: 16427830 [PubMed - indexed for MEDLINE]

[Related Articles](http://www.ncbi.nlm.nih.gov/sites/entrez?db=pubmed&cmd=link&linkname=pubmed_pubmed&uid=16427830&ordinalpos=183:)

# [Prevalence of antibodies to Neospora caninum in wild animals.](http://www.ncbi.nlm.nih.gov/pubmed/16419771?ordinalpos=185&itool=EntrezSystem2.PEntrez.Pubmed.Pubmed_ResultsPanel.Pubmed_DefaultReportPanel.Pubmed_RVDocSum)

Dubey JP, Thulliez P.

J Parasitol. 2005 Oct;91(5):1217-8.

PMID: 16419771 [PubMed - indexed for MEDLINE]

# [High prevalence of systemic lupus erythematosus in 78 myasthenia gravis patients: a clinical and serologic study.](http://www.ncbi.nlm.nih.gov/pubmed/16415656?ordinalpos=186&itool=EntrezSystem2.PEntrez.Pubmed.Pubmed_ResultsPanel.Pubmed_DefaultReportPanel.Pubmed_RVDocSum)

Sthoeger Z, Neiman A, Elbirt D, Zinger H, Magen E, Burstein R, Eitan S, Abarbanel J, Mozes E.

Am J Med Sci. 2006 Jan;331(1):4-9.

PMID: 16415656 [PubMed - indexed for MEDLINE]

# [[Striated muscular injury and myocardiac injury caused by acute carbon monoxide poisoning]](http://www.ncbi.nlm.nih.gov/pubmed/16405777?ordinalpos=187&itool=EntrezSystem2.PEntrez.Pubmed.Pubmed_ResultsPanel.Pubmed_DefaultReportPanel.Pubmed_RVDocSum)

Wang HT, Xu XX, Li YP.

Zhonghua Lao Dong Wei Sheng Zhi Ye Bing Za Zhi. 2005 Dec;23(6):435-7. Chinese.

PMID: 16405777 [PubMed - indexed for MEDLINE]

# [Risk of extubation failure in patients with myasthenic crisis.](http://www.ncbi.nlm.nih.gov/pubmed/16377831?ordinalpos=188&itool=EntrezSystem2.PEntrez.Pubmed.Pubmed_ResultsPanel.Pubmed_DefaultReportPanel.Pubmed_RVDocSum)

Rabinstein AA, Mueller-Kronast N.

Neurocrit Care. 2005;3(3):213-5.

PMID: 16377831 [PubMed - indexed for MEDLINE]

# [Myasthenic crisis.](http://www.ncbi.nlm.nih.gov/pubmed/16377829?ordinalpos=189&itool=EntrezSystem2.PEntrez.Pubmed.Pubmed_ResultsPanel.Pubmed_DefaultReportPanel.Pubmed_RVDocSum)

Lacomis D.

Neurocrit Care. 2005;3(3):189-94. Review.

PMID: 16377829 [PubMed - indexed for MEDLINE]

# [Transsternal thymectomy for myasthenia gravis: surgical outcome.](http://www.ncbi.nlm.nih.gov/pubmed/16368387?ordinalpos=190&itool=EntrezSystem2.PEntrez.Pubmed.Pubmed_ResultsPanel.Pubmed_DefaultReportPanel.Pubmed_RVDocSum)

Kattach H, Anastasiadis K, Cleuziou J, Buckley C, Shine B, Pillai R, Ratnatunga C.

Ann Thorac Surg. 2006 Jan;81(1):305-8.

PMID: 16368387 [PubMed - indexed for MEDLINE]

# [Idiopathic vocal cord palsies and associated neurological conditions.](http://www.ncbi.nlm.nih.gov/pubmed/16365222?ordinalpos=191&itool=EntrezSystem2.PEntrez.Pubmed.Pubmed_ResultsPanel.Pubmed_DefaultReportPanel.Pubmed_RVDocSum)

Urquhart AC, St Louis EK.

Arch Otolaryngol Head Neck Surg. 2005 Dec;131(12):1086-9. Erratum in: Arch Otolaryngol Head Neck Surg. 2006 Jun;132(6):594. St Louis, Erik [corrected to St Louis, Erik K].

PMID: 16365222 [PubMed - indexed for MEDLINE]

# [Target-controlled infusion of propofol in dogs--evaluation of four targets for induction of anaesthesia.](http://www.ncbi.nlm.nih.gov/pubmed/16339979?ordinalpos=192&itool=EntrezSystem2.PEntrez.Pubmed.Pubmed_ResultsPanel.Pubmed_DefaultReportPanel.Pubmed_RVDocSum)

Musk GC, Pang DS, Beths T, Flaherty DA.

Vet Rec. 2005 Dec 10;157(24):766-70.

PMID: 16339979 [PubMed - indexed for MEDLINE]

# [Surgical management of thymic epithelial tumors: a retrospective review of 204 cases.](http://www.ncbi.nlm.nih.gov/pubmed/16305833?ordinalpos=193&itool=EntrezSystem2.PEntrez.Pubmed.Pubmed_ResultsPanel.Pubmed_DefaultReportPanel.Pubmed_RVDocSum)

Fang W, Chen W, Chen G, Jiang Y.

Ann Thorac Surg. 2005 Dec;80(6):2002-7.

PMID: 16305833 [PubMed - indexed for MEDLINE]

# [Video-assisted thoracic surgery thymectomy for nonthymomatous myasthenia gravis.](http://www.ncbi.nlm.nih.gov/pubmed/16304299?ordinalpos=194&itool=EntrezSystem2.PEntrez.Pubmed.Pubmed_ResultsPanel.Pubmed_DefaultReportPanel.Pubmed_RVDocSum)

Manlulu A, Lee TW, Wan I, Law CY, Chang C, Garzon JC, Yim A.

Chest. 2005 Nov;128(5):3454-60.

PMID: 16304299 [PubMed - indexed for MEDLINE]

# [Effect of plasma exchange on carbamazepine levels in a patient with myasthenia gravis and epilepsy.](http://www.ncbi.nlm.nih.gov/pubmed/16302866?ordinalpos=195&itool=EntrezSystem2.PEntrez.Pubmed.Pubmed_ResultsPanel.Pubmed_DefaultReportPanel.Pubmed_RVDocSum)

Siddiqi ZA, Holt A, Ahmed SN.

Epilepsia. 2005 Nov;46(11):1841-2.

PMID: 16302866 [PubMed - indexed for MEDLINE]

# [The effect of asbestosis on lung cancer risk beyond the dose related effect of asbestos alone.](http://www.ncbi.nlm.nih.gov/pubmed/16299098?ordinalpos=196&itool=EntrezSystem2.PEntrez.Pubmed.Pubmed_ResultsPanel.Pubmed_DefaultReportPanel.Pubmed_RVDocSum)

Reid A, de Klerk N, Ambrosini GL, Olsen N, Pang SC, Berry G, Musk AW.

Occup Environ Med. 2005 Dec;62(12):885-9.

PMID: 16299098 [PubMed - indexed for MEDLINE]

# [Weakness in the ICU: Guillain-Barré syndrome, myasthenia gravis, and critical illness polyneuropathy/myopathy.](http://www.ncbi.nlm.nih.gov/pubmed/16286877?ordinalpos=197&itool=EntrezSystem2.PEntrez.Pubmed.Pubmed_ResultsPanel.Pubmed_DefaultReportPanel.Pubmed_RVDocSum)

Green DM.

Neurologist. 2005 Nov;11(6):338-47. Review.

PMID: 16286877 [PubMed - indexed for MEDLINE]

# [Infections and autoimmune diseases.](http://www.ncbi.nlm.nih.gov/pubmed/16278064?ordinalpos=198&itool=EntrezSystem2.PEntrez.Pubmed.Pubmed_ResultsPanel.Pubmed_DefaultReportPanel.Pubmed_RVDocSum)

Bach JF.

J Autoimmun. 2005;25 Suppl:74-80. Epub 2005 Nov 8. Review.

PMID: 16278064 [PubMed - indexed for MEDLINE]

[Related Articles](http://www.ncbi.nlm.nih.gov/sites/entrez?db=pubmed&cmd=link&linkname=pubmed_pubmed&uid=16278064&ordinalpos=198:)

# [[Use of fragrances. What about the side effects?]](http://www.ncbi.nlm.nih.gov/pubmed/16270184?ordinalpos=199&itool=EntrezSystem2.PEntrez.Pubmed.Pubmed_ResultsPanel.Pubmed_DefaultReportPanel.Pubmed_RVDocSum)

Straff W.

Bundesgesundheitsblatt Gesundheitsforschung Gesundheitsschutz. 2005 Dec;48(12):1400-5. Review. German.

PMID: 16270184 [PubMed - indexed for MEDLINE]

# [Pregnancy complicated by Myasthenia gravis - twelve years experience.](http://www.ncbi.nlm.nih.gov/pubmed/16264394?ordinalpos=200&itool=EntrezSystem2.PEntrez.Pubmed.Pubmed_ResultsPanel.Pubmed_DefaultReportPanel.Pubmed_RVDocSum)

Podciechowski L, Brocka-Nitecka U, Dabrowska K, Bielak A, Hadacz B, Wilczynski J.

Neuro Endocrinol Lett. 2005 Oct;26(5):603-8.

PMID: 16264394 [PubMed - indexed for MEDLINE]

[Related Articles](http://www.ncbi.nlm.nih.gov/sites/entrez?db=pubmed&cmd=link&linkname=pubmed_pubmed&uid=16264394&ordinalpos=200:)

# [Bilateral video-assisted thoracoscopic thymectomy vs. extended transsternal thymectomy in myasthenia gravis: a prospective study.](http://www.ncbi.nlm.nih.gov/pubmed/16260868?ordinalpos=201&itool=EntrezSystem2.PEntrez.Pubmed.Pubmed_ResultsPanel.Pubmed_DefaultReportPanel.Pubmed_RVDocSum)

Chang PC, Chou SH, Kao EL, Cheng YJ, Chuang HY, Liu CK, Lai CL, Huang MF.

Eur Surg Res. 2005 Jul-Aug;37(4):199-203.

PMID: 16260868 [PubMed - indexed for MEDLINE]

# [Sleep and neuromuscular disorders.](http://www.ncbi.nlm.nih.gov/pubmed/16243623?ordinalpos=202&itool=EntrezSystem2.PEntrez.Pubmed.Pubmed_ResultsPanel.Pubmed_DefaultReportPanel.Pubmed_RVDocSum)

Culebras A.

Neurol Clin. 2005 Nov;23(4):1209-23, ix. Review.

PMID: 16243623 [PubMed - indexed for MEDLINE]

# [[Myasthenia in elderly patients: a series of 23 cases]](http://www.ncbi.nlm.nih.gov/pubmed/16229927?ordinalpos=203&itool=EntrezSystem2.PEntrez.Pubmed.Pubmed_ResultsPanel.Pubmed_DefaultReportPanel.Pubmed_RVDocSum)

Durand F, Camdessanché JP, Jomir L, Antoine JC, Cathébras P.

Rev Med Interne. 2005 Dec;26(12):924-30. Epub 2005 Sep 29. French.

PMID: 16229927 [PubMed - indexed for MEDLINE]

# [Thymus alterations and systemic sclerosis.](http://www.ncbi.nlm.nih.gov/pubmed/16188948?ordinalpos=205&itool=EntrezSystem2.PEntrez.Pubmed.Pubmed_ResultsPanel.Pubmed_DefaultReportPanel.Pubmed_RVDocSum)

Ferri C, Colaci M, Battolla L, Giuggioli D, Sebastiani M.

Rheumatology (Oxford). 2006 Jan;45(1):72-5. Epub 2005 Sep 27.

PMID: 16188948 [PubMed - indexed for MEDLINE]

# [Use of intravenous immunoglobulin for treatment of neurologic conditions: a systematic review.](http://www.ncbi.nlm.nih.gov/pubmed/16181216?ordinalpos=207&itool=EntrezSystem2.PEntrez.Pubmed.Pubmed_ResultsPanel.Pubmed_DefaultReportPanel.Pubmed_RVDocSum)

Fergusson D, Hutton B, Sharma M, Tinmouth A, Wilson K, Cameron DW, Hebert PC.

Transfusion. 2005 Oct;45(10):1640-57. Review.

PMID: 16181216 [PubMed - indexed for MEDLINE]

# [A CTLA4high genotype is associated with myasthenia gravis in thymoma patients.](http://www.ncbi.nlm.nih.gov/pubmed/16178018?ordinalpos=208&itool=EntrezSystem2.PEntrez.Pubmed.Pubmed_ResultsPanel.Pubmed_DefaultReportPanel.Pubmed_RVDocSum)

Chuang WY, Ströbel P, Gold R, Nix W, Schalke B, Kiefer R, Opitz A, Klinker E, Müller-Hermelink HK, Marx A.

Ann Neurol. 2005 Oct;58(4):644-8.

PMID: 16178018 [PubMed - indexed for MEDLINE]

# [The role of the thymus in the pathogenesis of myasthenia gravis.](http://www.ncbi.nlm.nih.gov/pubmed/16141677?ordinalpos=209&itool=EntrezSystem2.PEntrez.Pubmed.Pubmed_ResultsPanel.Pubmed_DefaultReportPanel.Pubmed_RVDocSum)

Onodera H.

Tohoku J Exp Med. 2005 Oct;207(2):87-98. Review.

PMID: 16141677 [PubMed - indexed for MEDLINE]

# [Prognostic factors in thymic epithelial tumors undergoing complete resection.](http://www.ncbi.nlm.nih.gov/pubmed/16122486?ordinalpos=210&itool=EntrezSystem2.PEntrez.Pubmed.Pubmed_ResultsPanel.Pubmed_DefaultReportPanel.Pubmed_RVDocSum)

Zisis C, Rontogianni D, Tzavara C, Stefanaki K, Chatzimichalis A, Loutsidis A, Iliadis K, Kontaxis A, Dosios T, Bellenis I.

Ann Thorac Surg. 2005 Sep;80(3):1056-62.

PMID: 16122486 [PubMed - indexed for MEDLINE]

# [Sudden death due to asphyxia by esophageal polyp: two case reports and review of asphyxial deaths.](http://www.ncbi.nlm.nih.gov/pubmed/16121086?ordinalpos=211&itool=EntrezSystem2.PEntrez.Pubmed.Pubmed_ResultsPanel.Pubmed_DefaultReportPanel.Pubmed_RVDocSum)

Carrick C, Collins KA, Lee CJ, Prahlow JA, Barnard JJ.

Am J Forensic Med Pathol. 2005 Sep;26(3):275-81. Review.

PMID: 16121086 [PubMed - indexed for MEDLINE]

# [[Thoracoscopic thymectomy in the treatment of myasthenia gravis]](http://www.ncbi.nlm.nih.gov/pubmed/16106927?ordinalpos=212&itool=EntrezSystem2.PEntrez.Pubmed.Pubmed_ResultsPanel.Pubmed_DefaultReportPanel.Pubmed_RVDocSum)

Tomulescu V, Ion V, Kosa A, Popescu I.

Chirurgia (Bucur). 2005 May-Jun;100(3):215-22. Romanian.

PMID: 16106927 [PubMed - indexed for MEDLINE]

# [Surgical treatment of thymic tumors.](http://www.ncbi.nlm.nih.gov/pubmed/16104357?ordinalpos=213&itool=EntrezSystem2.PEntrez.Pubmed.Pubmed_ResultsPanel.Pubmed_DefaultReportPanel.Pubmed_RVDocSum)

Wright CD, Kessler KA.

Semin Thorac Cardiovasc Surg. 2005 Spring;17(1):20-6. Review.

PMID: 16104357 [PubMed - indexed for MEDLINE]

# [Effects of asbestos and smoking on gas diffusion in people exposed to crocidolite.](http://www.ncbi.nlm.nih.gov/pubmed/16097914?ordinalpos=214&itool=EntrezSystem2.PEntrez.Pubmed.Pubmed_ResultsPanel.Pubmed_DefaultReportPanel.Pubmed_RVDocSum)

Alfonso HS, Fritschi L, de Klerk NH, Olsen N, Sleith J, Musk AB.

Med J Aust. 2005 Aug 15;183(4):184-7.

PMID: 16097914 [PubMed - indexed for MEDLINE]

[Related Articles](http://www.ncbi.nlm.nih.gov/sites/entrez?db=pubmed&cmd=link&linkname=pubmed_pubmed&uid=16097914&ordinalpos=214:)

# [[Thymoma in elderly patients]](http://www.ncbi.nlm.nih.gov/pubmed/16097629?ordinalpos=215&itool=EntrezSystem2.PEntrez.Pubmed.Pubmed_ResultsPanel.Pubmed_DefaultReportPanel.Pubmed_RVDocSum)

Yano M, Fujii Y.

Kyobu Geka. 2005 Jul;58(8 Suppl):739-44. Review. Japanese.

PMID: 16097629 [PubMed - indexed for MEDLINE]

# [Respiratory complications of rapidly progressive neuromuscular syndromes: Guillain-Barré syndrome and myasthenia gravis.](http://www.ncbi.nlm.nih.gov/pubmed/16088614?ordinalpos=216&itool=EntrezSystem2.PEntrez.Pubmed.Pubmed_ResultsPanel.Pubmed_DefaultReportPanel.Pubmed_RVDocSum)

Yavagal DR, Mayer SA.

Semin Respir Crit Care Med. 2002 Jun;23(3):221-9.

PMID: 16088614 [PubMed]

# [IL-1 receptor antagonist-mediated therapeutic effect in murine myasthenia gravis is associated with suppressed serum proinflammatory cytokines, C3, and anti-acetylcholine receptor IgG1.](http://www.ncbi.nlm.nih.gov/pubmed/16034147?ordinalpos=217&itool=EntrezSystem2.PEntrez.Pubmed.Pubmed_ResultsPanel.Pubmed_DefaultReportPanel.Pubmed_RVDocSum)

Yang H, Tüzün E, Alagappan D, Yu X, Scott BG, Ischenko A, Christadoss P.

J Immunol. 2005 Aug 1;175(3):2018-25.

PMID: 16034147 [PubMed - indexed for MEDLINE]

# [[Video-assisted thoracoscopic extended thymectomy for myasthenia gravis: analysis of 107 cases]](http://www.ncbi.nlm.nih.gov/pubmed/16008926?ordinalpos=220&itool=EntrezSystem2.PEntrez.Pubmed.Pubmed_ResultsPanel.Pubmed_DefaultReportPanel.Pubmed_RVDocSum)

Liu HP, Li JF, Wu YC, Xie MR, Liu YH, Jiang GC, Liu J, Wang J.

Zhonghua Wai Ke Za Zhi. 2005 May 15;43(10):625-7. Chinese.

PMID: 16008926 [PubMed - indexed for MEDLINE]

# [Factors influencing the outcome of transsternal thymectomy for myasthenia gravis.](http://www.ncbi.nlm.nih.gov/pubmed/16008537?ordinalpos=221&itool=EntrezSystem2.PEntrez.Pubmed.Pubmed_ResultsPanel.Pubmed_DefaultReportPanel.Pubmed_RVDocSum)

Huang CS, Hsu HS, Huang BS, Lee HC, Kao KP, Hsu WH, Huang MH.

Acta Neurol Scand. 2005 Aug;112(2):108-14.

PMID: 16008537 [PubMed - indexed for MEDLINE]

# [Myasthenia gravis and hypothyroidism in a dog with meningomyelitis.](http://www.ncbi.nlm.nih.gov/pubmed/15995162?ordinalpos=222&itool=EntrezSystem2.PEntrez.Pubmed.Pubmed_ResultsPanel.Pubmed_DefaultReportPanel.Pubmed_RVDocSum)

Levine JM, Bergman RL, Coates JR, Shelton GD.

J Am Anim Hosp Assoc. 2005 Jul-Aug;41(4):247-51.

PMID: 15995162 [PubMed - indexed for MEDLINE]

# [What can Busselton population health surveys tell us about asthma in older people?](http://www.ncbi.nlm.nih.gov/pubmed/15992314?ordinalpos=223&itool=EntrezSystem2.PEntrez.Pubmed.Pubmed_ResultsPanel.Pubmed_DefaultReportPanel.Pubmed_RVDocSum)

James AL, Knuiman MW, Bartholomew HC, Musk AB.

Med J Aust. 2005 Jul 4;183(1 Suppl):S17-9.

PMID: 15992314 [PubMed - indexed for MEDLINE]

# [Extrathymic malignancies in patients with myasthenia gravis.](http://www.ncbi.nlm.nih.gov/pubmed/15990114?ordinalpos=224&itool=EntrezSystem2.PEntrez.Pubmed.Pubmed_ResultsPanel.Pubmed_DefaultReportPanel.Pubmed_RVDocSum)

Levin N, Abramsky O, Lossos A, Karussis D, Siegal T, Argov Z, Ben Hur T.

J Neurol Sci. 2005 Oct 15;237(1-2):39-43.

# [["Seronegative" myasthenia gravis and antiMuSK positive antibodies: description of Spanish series]](http://www.ncbi.nlm.nih.gov/pubmed/15989843?ordinalpos=225&itool=EntrezSystem2.PEntrez.Pubmed.Pubmed_ResultsPanel.Pubmed_DefaultReportPanel.Pubmed_RVDocSum)

Illa I, Díaz-Manera JA, Juárez C, Rojas-García R, Molina-Porcel L, Aleu A, Pradas J, Gallardo E.

Med Clin (Barc). 2005 Jun 18;125(3):100-2. Spanish.

PMID: 15989843 [PubMed - indexed for MEDLINE]

# [[Neurological appearances of primary antiphospholipid syndrome]](http://www.ncbi.nlm.nih.gov/pubmed/15986822?ordinalpos=226&itool=EntrezSystem2.PEntrez.Pubmed.Pubmed_ResultsPanel.Pubmed_DefaultReportPanel.Pubmed_RVDocSum)

Kalashnikova LA, Dobrynina LA, Aleksandrova EN, Novikov AA.

Zh Nevrol Psikhiatr Im S S Korsakova. 2005;(Suppl 13):19-24. Russian.

PMID: 15986822 [PubMed - indexed for MEDLINE]

# [Unusual reactions to insect stings.](http://www.ncbi.nlm.nih.gov/pubmed/15985819?ordinalpos=227&itool=EntrezSystem2.PEntrez.Pubmed.Pubmed_ResultsPanel.Pubmed_DefaultReportPanel.Pubmed_RVDocSum)

Reisman RE.

Curr Opin Allergy Clin Immunol. 2005 Aug;5(4):355-8. Review.

PMID: 15985819 [PubMed - indexed for MEDLINE]

# [[Neurological diseases and pregnancy: what must the family physician heed?]](http://www.ncbi.nlm.nih.gov/pubmed/15968871?ordinalpos=228&itool=EntrezSystem2.PEntrez.Pubmed.Pubmed_ResultsPanel.Pubmed_DefaultReportPanel.Pubmed_RVDocSum)

Block F.

MMW Fortschr Med. 2005 May 17;147 Spec No 2:40-3. German.

PMID: 15968871 [PubMed - indexed for MEDLINE]

# [Myasthenia gravis accompanied by alopecia areata: clinical and immunogenetic aspects.](http://www.ncbi.nlm.nih.gov/pubmed/15958099?ordinalpos=229&itool=EntrezSystem2.PEntrez.Pubmed.Pubmed_ResultsPanel.Pubmed_DefaultReportPanel.Pubmed_RVDocSum)

Suzuki S, Shimoda M, Kawamura M, Sato H, Nogawa S, Tanaka K, Suzuki N, Kuwana M.

Eur J Neurol. 2005 Jul;12(7):566-70.

PMID: 15958099 [PubMed - indexed for MEDLINE]

# [[Primary antiphospholipid syndrome and cerebrovascular disturbances]](http://www.ncbi.nlm.nih.gov/pubmed/15952534?ordinalpos=230&itool=EntrezSystem2.PEntrez.Pubmed.Pubmed_ResultsPanel.Pubmed_DefaultReportPanel.Pubmed_RVDocSum)

Kalashnikova LA.

Zh Nevrol Psikhiatr Im S S Korsakova. 2005;105(5):11-6. Russian.

# [Plasma concentrations of retinol, carotene, and vitamin E and mortality in subjects with asbestosis in a cohort exposed to crocidolite in Wittenoom, Western Australia.](http://www.ncbi.nlm.nih.gov/pubmed/15951717?ordinalpos=231&itool=EntrezSystem2.PEntrez.Pubmed.Pubmed_ResultsPanel.Pubmed_DefaultReportPanel.Pubmed_RVDocSum)

Alfonso HS, Fritschi L, de Klerk NH, Ambrosini G, Beilby J, Olsen N, Musk AW.

J Occup Environ Med. 2005 Jun;47(6):573-9. Erratum in: J Occup Environ Med. 2005 Sep;47(9):972.

PMID: 15951717 [PubMed - indexed for MEDLINE]

# [[Autoimmune diseases and multiple sclerosis]](http://www.ncbi.nlm.nih.gov/pubmed/15926131?ordinalpos=233&itool=EntrezSystem2.PEntrez.Pubmed.Pubmed_ResultsPanel.Pubmed_DefaultReportPanel.Pubmed_RVDocSum)

Alemany-Rodríguez MJ, Aladro Y, Amela-Peris R, Pérez-Viéitez MC, Reyes-Yáñez MP, Déniz-Naranjo MC, Sánchez-Garcia F.

Rev Neurol. 2005 May 16-31;40(10):594-7. Spanish.

PMID: 15926131 [PubMed - indexed for MEDLINE]

# [Comparison between video-assisted thoracoscopic thymectomy and transternal thymectomy for myasthenia gravis (analysis of 82 cases).](http://www.ncbi.nlm.nih.gov/pubmed/15912898?ordinalpos=234&itool=EntrezSystem2.PEntrez.Pubmed.Pubmed_ResultsPanel.Pubmed_DefaultReportPanel.Pubmed_RVDocSum)

Lin TS, Tzao C, Lee SC, Wu CY, Shy CJ, Lee CY, Chou MC.

Int Surg. 2005 Jan-Mar;90(1):36-41.

PMID: 15912898 [PubMed - indexed for MEDLINE]

# [Clinical utility of videofluorography with concomitant Tensilon administration in the diagnosis of bulbar myasthenia gravis.](http://www.ncbi.nlm.nih.gov/pubmed/15906757?ordinalpos=235&itool=EntrezSystem2.PEntrez.Pubmed.Pubmed_ResultsPanel.Pubmed_DefaultReportPanel.Pubmed_RVDocSum)

Schwartz DC, Waclawik AJ, Ringwala SN, Robbins J.

Dig Dis Sci. 2005 May;50(5):858-61.

PMID: 15906757 [PubMed - indexed for MEDLINE]

# [Simultaneous congener-specific determination of selected organochlorine compounds and nitro musks in human whole blood samples by solid-phase extraction and capillary gas chromatography with electron capture detection.](http://www.ncbi.nlm.nih.gov/pubmed/15902979?ordinalpos=236&itool=EntrezSystem2.PEntrez.Pubmed.Pubmed_ResultsPanel.Pubmed_DefaultReportPanel.Pubmed_RVDocSum)

Bauer M, Hilpert F, Arnold N, Pfisterer J, Jonat W, Kruse H.

J Anal Toxicol. 2005 Mar;29(2):110-7.

PMID: 15902979 [PubMed - indexed for MEDLINE]

# [Seronegative myasthenia gravis: disease severity and prognosis.](http://www.ncbi.nlm.nih.gov/pubmed/15885043?ordinalpos=237&itool=EntrezSystem2.PEntrez.Pubmed.Pubmed_ResultsPanel.Pubmed_DefaultReportPanel.Pubmed_RVDocSum)

Romi F, Aarli JA, Gilhus NE.

Eur J Neurol. 2005 Jun;12(6):413-8.

PMID: 15885043 [PubMed - indexed for MEDLINE]

# [[Myasthenia gravis and pregnancy]](http://www.ncbi.nlm.nih.gov/pubmed/15847079?ordinalpos=238&itool=EntrezSystem2.PEntrez.Pubmed.Pubmed_ResultsPanel.Pubmed_DefaultReportPanel.Pubmed_RVDocSum)

Kostera-Pruszczyk A, Emeryk-Szajewska B.

Ginekol Pol. 2005 Feb;76(2):122-6. Polish.

PMID: 15847079 [PubMed - indexed for MEDLINE]

# [Does this patient have myasthenia gravis?](http://www.ncbi.nlm.nih.gov/pubmed/15840866?ordinalpos=239&itool=EntrezSystem2.PEntrez.Pubmed.Pubmed_ResultsPanel.Pubmed_DefaultReportPanel.Pubmed_RVDocSum)

Scherer K, Bedlack RS, Simel DL.

JAMA. 2005 Apr 20;293(15):1906-14. Review.

PMID: 15840866 [PubMed - indexed for MEDLINE]

# [Outcome after transsternal radical thymectomy for myasthenia gravis: 14-year review at Ratchaburi Hospital.](http://www.ncbi.nlm.nih.gov/pubmed/15825704?ordinalpos=240&itool=EntrezSystem2.PEntrez.Pubmed.Pubmed_ResultsPanel.Pubmed_DefaultReportPanel.Pubmed_RVDocSum)

Glinjongol C, Paiboonpol S.

J Med Assoc Thai. 2004 Nov;87(11):1304-10.

PMID: 15825704 [PubMed - indexed for MEDLINE]

# [Myasthenic crisis: clinical features, complications and mortality.](http://www.ncbi.nlm.nih.gov/pubmed/15805653?ordinalpos=241&itool=EntrezSystem2.PEntrez.Pubmed.Pubmed_ResultsPanel.Pubmed_DefaultReportPanel.Pubmed_RVDocSum)

Murthy JM, Meena AK, Chowdary GV, Naryanan JT.

Neurol India. 2005 Mar;53(1):37-40; discussion 40.

PMID: 15805653 [PubMed - indexed for MEDLINE]

# [[Surgery of the thymus]](http://www.ncbi.nlm.nih.gov/pubmed/15803873?ordinalpos=242&itool=EntrezSystem2.PEntrez.Pubmed.Pubmed_ResultsPanel.Pubmed_DefaultReportPanel.Pubmed_RVDocSum)

Kas J, Besznyák I, Kocsis A, Major L, Saortay S, Svastics E.

Magy Seb. 2004 Dec;57(6):311-9. Hungarian.

PMID: 15803873 [PubMed - indexed for MEDLINE]

# [beta2-adrenergic receptor gene single-nucleotide polymorphisms are associated with rheumatoid arthritis in northern Sweden.](http://www.ncbi.nlm.nih.gov/pubmed/15794198?ordinalpos=243&itool=EntrezSystem2.PEntrez.Pubmed.Pubmed_ResultsPanel.Pubmed_DefaultReportPanel.Pubmed_RVDocSum)

Xu B, Arlehag L, Rantapää-Dahlquist SB, Lefvert AK.

Scand J Rheumatol. 2004;33(6):395-8.

PMID: 15794198 [PubMed - indexed for MEDLINE]

# [Video-assisted transcervical thymectomy.](http://www.ncbi.nlm.nih.gov/pubmed/15791384?ordinalpos=244&itool=EntrezSystem2.PEntrez.Pubmed.Pubmed_ResultsPanel.Pubmed_DefaultReportPanel.Pubmed_RVDocSum)

Bramis J, Diamantis T, Tsigris C, Pikoulis E, Papaconstantinou I, Nikolaou A, Leonardou P, Bastounis E.

Surg Endosc. 2004 Oct;18(10):1535-8. Epub 2004 Aug 26.

PMID: 15791384 [PubMed - indexed for MEDLINE]

# [Renal and thymic pathology in thymoma-associated nephropathy: report of 21 cases and review of the literature.](http://www.ncbi.nlm.nih.gov/pubmed/15788438?ordinalpos=245&itool=EntrezSystem2.PEntrez.Pubmed.Pubmed_ResultsPanel.Pubmed_DefaultReportPanel.Pubmed_RVDocSum)

Karras A, de Montpreville V, Fakhouri F, Grünfeld JP, Lesavre P; Groupe d'Etudes des Néphropathies Associées aux Thymomes.

Nephrol Dial Transplant. 2005 Jun;20(6):1075-82. Epub 2005 Mar 23. Review.

PMID: 15788438 [PubMed - indexed for MEDLINE]

# [Manubriotomy versus median sternotomy in thymectomy for myasthenia gravis. Evaluation of the pulmonary status.](http://www.ncbi.nlm.nih.gov/pubmed/15740939?ordinalpos=246&itool=EntrezSystem2.PEntrez.Pubmed.Pubmed_ResultsPanel.Pubmed_DefaultReportPanel.Pubmed_RVDocSum)

Granetzny A, Hatem A, Shalaby A, Boseila A.

Eur J Cardiothorac Surg. 2005 Mar;27(3):361-6. Epub 2004 Dec 30.

PMID: 15740939 [PubMed - indexed for MEDLINE]

# [Beneficial effects of plasmapheresis before thymectomy on the outcome in myasthenia gravis.](http://www.ncbi.nlm.nih.gov/pubmed/15724495?ordinalpos=247&itool=EntrezSystem2.PEntrez.Pubmed.Pubmed_ResultsPanel.Pubmed_DefaultReportPanel.Pubmed_RVDocSum)

Nagayasu T, Yamayoshi T, Matsumoto K, Ide N, Hashizume S, Nomura M, Muraoka M, Tagawa T, Akamine S, Oka T.

Jpn J Thorac Cardiovasc Surg. 2005 Jan;53(1):2-7.

PMID: 15724495 [PubMed - indexed for MEDLINE]

# [Autoantibodies to a NR2A peptide of the glutamate/NMDA receptor in sera of patients with systemic lupus erythematosus.](http://www.ncbi.nlm.nih.gov/pubmed/15708887?ordinalpos=248&itool=EntrezSystem2.PEntrez.Pubmed.Pubmed_ResultsPanel.Pubmed_DefaultReportPanel.Pubmed_RVDocSum)

Husebye ES, Sthoeger ZM, Dayan M, Zinger H, Elbirt D, Levite M, Mozes E.

Ann Rheum Dis. 2005 Aug;64(8):1210-3. Epub 2005 Feb 11.

PMID: 15708887 [PubMed - indexed for MEDLINE]

# [[Thymectomy for myastenia gravis: 25-year experience]](http://www.ncbi.nlm.nih.gov/pubmed/15702874?ordinalpos=249&itool=EntrezSystem2.PEntrez.Pubmed.Pubmed_ResultsPanel.Pubmed_DefaultReportPanel.Pubmed_RVDocSum)

Petkov R, Kŭtev N, Mladenovski V, Todorov G, Gavrilov N, Atanasov A, Goranov N.

Khirurgiia (Sofiia). 2004;60(3):27-9. Bulgarian.

PMID: 15702874 [PubMed - indexed for MEDLINE]

# [Is "seronegative" MG explained by autoantibodies to MuSK?](http://www.ncbi.nlm.nih.gov/pubmed/15668461?ordinalpos=250&itool=EntrezSystem2.PEntrez.Pubmed.Pubmed_ResultsPanel.Pubmed_DefaultReportPanel.Pubmed_RVDocSum)

Vincent AC, McConville J, Newsom-Davis J.

Neurology. 2005 Jan 25;64(2):399; author reply 399. No abstract available.

PMID: 15668461 [PubMed - indexed for MEDLINE]

# [Myasthenic crisis: a retrospective study.](http://www.ncbi.nlm.nih.gov/pubmed/15626832?ordinalpos=251&itool=EntrezSystem2.PEntrez.Pubmed.Pubmed_ResultsPanel.Pubmed_DefaultReportPanel.Pubmed_RVDocSum)

Panda S, Goyal V, Behari M, Singh S, Srivastava T.

Neurol India. 2004 Dec;52(4):453-6.

PMID: 15626832 [PubMed - indexed for MEDLINE]

# [Thymoma and myasthenia gravis: a clinical study of 1,089 patients from Japan.](http://www.ncbi.nlm.nih.gov/pubmed/15620947?ordinalpos=252&itool=EntrezSystem2.PEntrez.Pubmed.Pubmed_ResultsPanel.Pubmed_DefaultReportPanel.Pubmed_RVDocSum)

Kondo K, Monden Y.

Ann Thorac Surg. 2005 Jan;79(1):219-24.

PMID: 15620947 [PubMed - indexed for MEDLINE]

# [[Disease associations in 250 patients with temporal (giant cell) arteritis]](http://www.ncbi.nlm.nih.gov/pubmed/15615235?ordinalpos=253&itool=EntrezSystem2.PEntrez.Pubmed.Pubmed_ResultsPanel.Pubmed_DefaultReportPanel.Pubmed_RVDocSum)

Liozon E, Loustaud-Ratti V, Soria P, Bezanahary H, Fauchais AL, Nadalon S, Rhaiem K, Ly K, Vidal E.

Presse Med. 2004 Nov 6;33(19 Pt 1):1304-12. French.

PMID: 15615235 [PubMed - indexed for MEDLINE]

# [Neuromuscular Manifestations of Human West Nile Virus Infection.](http://www.ncbi.nlm.nih.gov/pubmed/15610703?ordinalpos=254&itool=EntrezSystem2.PEntrez.Pubmed.Pubmed_ResultsPanel.Pubmed_DefaultReportPanel.Pubmed_RVDocSum)

Leis AA, Stokic DS.

Curr Treat Options Neurol. 2005 Jan;7(1):15-22.

PMID: 15610703 [PubMed - as supplied by publisher]

# [Update on myasthenia gravis.](http://www.ncbi.nlm.nih.gov/pubmed/15579606?ordinalpos=255&itool=EntrezSystem2.PEntrez.Pubmed.Pubmed_ResultsPanel.Pubmed_DefaultReportPanel.Pubmed_RVDocSum)

Thanvi BR, Lo TC.

Postgrad Med J. 2004 Dec;80(950):690-700. Review.

PMID: 15579606 [PubMed - indexed for MEDLINE]

# [Respiratory failure as a first presentation of myasthenia gravis.](http://www.ncbi.nlm.nih.gov/pubmed/15567987?ordinalpos=256&itool=EntrezSystem2.PEntrez.Pubmed.Pubmed_ResultsPanel.Pubmed_DefaultReportPanel.Pubmed_RVDocSum)

Qureshi AI, Choundry MA, Mohammad Y, Chua HC, Yahia AM, Ulatowski JA, Krendel DA, Leshner RT.

Med Sci Monit. 2004 Dec;10(12):CR684-9.

PMID: 15567987 [PubMed - indexed for MEDLINE]

# [Effects of asbestos and smoking on the levels and rates of change of lung function in a crocidolite exposed cohort in Western Australia.](http://www.ncbi.nlm.nih.gov/pubmed/15563704?ordinalpos=257&itool=EntrezSystem2.PEntrez.Pubmed.Pubmed_ResultsPanel.Pubmed_DefaultReportPanel.Pubmed_RVDocSum)

Alfonso HS, Fritschi L, de Klerk NH, Olsen N, Sleith J, Musk AW.

Thorax. 2004 Dec;59(12):1052-6.

PMID: 15563704 [PubMed - indexed for MEDLINE]

# [Autoantibody profiles and neurological correlations of thymoma.](http://www.ncbi.nlm.nih.gov/pubmed/15534101?ordinalpos=258&itool=EntrezSystem2.PEntrez.Pubmed.Pubmed_ResultsPanel.Pubmed_DefaultReportPanel.Pubmed_RVDocSum)

Vernino S, Lennon VA.

Clin Cancer Res. 2004 Nov 1;10(21):7270-5.

PMID: 15534101 [PubMed - indexed for MEDLINE]

# [Minimally invasive gastric bypass in a morbidly obese patient with myasthenia gravis.](http://www.ncbi.nlm.nih.gov/pubmed/15527647?ordinalpos=259&itool=EntrezSystem2.PEntrez.Pubmed.Pubmed_ResultsPanel.Pubmed_DefaultReportPanel.Pubmed_RVDocSum)

Schumann R, Tarnoff M, Siddiqui ZI.

Obes Surg. 2004 Oct;14(9):1273-6.

PMID: 15527647 [PubMed - indexed for MEDLINE]

# [Radiotherapy and prognostic factors for thymoma: a retrospective study of 175 patients.](http://www.ncbi.nlm.nih.gov/pubmed/15519782?ordinalpos=260&itool=EntrezSystem2.PEntrez.Pubmed.Pubmed_ResultsPanel.Pubmed_DefaultReportPanel.Pubmed_RVDocSum)

Zhu G, He S, Fu X, Jiang G, Liu T.

Int J Radiat Oncol Biol Phys. 2004 Nov 15;60(4):1113-9.

PMID: 15519782 [PubMed - indexed for MEDLINE]
